# The evolution of function within the Nudix homology clan

**DOI:** 10.1002/prot.25223

**Published:** 2017-03-16

**Authors:** John R. Srouji, Anting Xu, Annsea Park, Jack F. Kirsch, Steven E. Brenner

**Affiliations:** ^1^ Plant and Microbial Biology DepartmentUniversity of CaliforniaBerkeleyCalifornia94720; ^2^ Molecular and Cell Biology DepartmentUniversity of CaliforniaBerkeleyCalifornia94720; ^3^ Graduate Study in Comparative Biochemistry, University of CaliforniaBerkeleyCalifornia94720; ^4^ Present address: Molecular and Cellular Biology DepartmentHarvard UniversityCambridgeMassachusetts02138

**Keywords:** hydrolase, homoplasy, Nudix homology clan, sequence alignment, structural alignment, Nudix

## Abstract

The Nudix homology clan encompasses over 80,000 protein domains from all three domains of life, defined by homology to each other. Proteins with a domain from this clan fall into four general functional classes: pyrophosphohydrolases, isopentenyl diphosphate isomerases (IDIs), adenine/guanine mismatch‐specific adenine glycosylases (A/G‐specific adenine glycosylases), and nonenzymatic activities such as protein/protein interaction and transcriptional regulation. The largest group, pyrophosphohydrolases, encompasses more than 100 distinct hydrolase specificities. To understand the evolution of this vast number of activities, we assembled and analyzed experimental and structural data for 205 Nudix proteins collected from the literature. We corrected erroneous functions or provided more appropriate descriptions for 53 annotations described in the Gene Ontology Annotation database in this family, and propose 275 new experimentally‐based annotations. We manually constructed a structure‐guided sequence alignment of 78 Nudix proteins. Using the structural alignment as a seed, we then made an alignment of 347 “select” Nudix homology domains, curated from structurally determined, functionally characterized, or phylogenetically important Nudix domains. Based on our review of Nudix pyrophosphohydrolase structures and specificities, we further analyzed a loop region downstream of the Nudix hydrolase motif previously shown to contact the substrate molecule and possess known functional motifs. This loop region provides a potential structural basis for the functional radiation and evolution of substrate specificity within the hydrolase family. Finally, phylogenetic analyses of the 347 select protein domains and of the complete Nudix homology clan revealed general monophyly with regard to function and a few instances of probable homoplasy. Proteins 2017; 85:775–811. © 2016 Wiley Periodicals, Inc.

## INTRODUCTION

**Figure 1:**
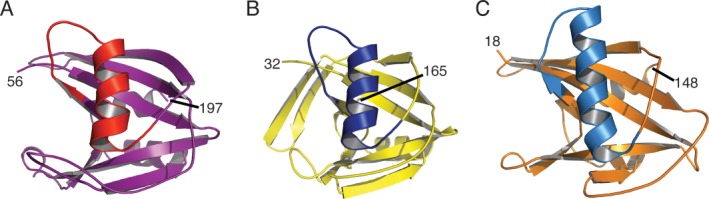
Structural conservation within the Nudix homology clan. The overall architecture of the Nudix homology domain (∼130 amino acids) is preserved across three enzyme families (hydrolase, isopentenyl diphosphate isomerase, and adenine/guanine‐specific adenine glycosylase) as well as Nudix homology proteins having nonenzymatic functions. Despite the structural similarity, sequence identity within the Nudix homology domain between these proteins is low: *E. coli* ADP‐ribose diphosphatase shares 11.4% and 15.3% amino acid sequence identity with human A/G‐specific adenine glycosylase and *E. coli* isopentenyl diphosphate isomerase, respectively. And human A/G‐specific adenine glycosylase shares 13.0% identity with *E. coli* isopentenyl diphosphate. Representative structures (N and C‐terminal residue numbers indicated) from the (**A**) Nudix hydrolase family (represented by *Escherichia coli* ADP‐ribose diphosphatase—PDB^92^ ID: 1KHZ).^105^ The characteristic 23 amino acid Nudix pyrophosphohydrolase motif (residues 97–119; GX_5_EX_7_REUXEEXGU, where U is a hydrophobic residue and X is any amino acid) constitutes a loop‐helix‐loop structure that is conserved across the Nudix hydrolases and is shown in red. (**B**) The isopentenyl diphosphate isomerase family (represented by *E. coli* isopentenyl diphosphate isomerase—PDB ID: 1NFS),^97^ with GX_5_EX_7_RRAXEEXGI in blue (residues 68–90), and (**C**) A/G‐specific adenine glycosylase family (represented by human A/G‐specific adenine glycosylase—PDB ID: 1X51),^94^ with VX_2_EX_11_QELXRWXG in light blue (residues 56–78). The structures were visualized with PyMOL v0.99^67^ and graphics processed with Adobe Illustrator CS4^114^.

The Nudix homology clan is a large, evolutionarily related group of proteins found in organisms from all three domains of cellular life and in viruses. In Pfam (v27.0, March 2013),^1^ five Pfam protein families were classified under the clan (CL0261) named “Nudix Superfamily”: NUDIX (PF00293), DBC1 (PF14443), NUDIX‐like (PF09296), NUDIX_2 (PF14815), and NUDIX_4 (PF13869). These proteins fall into four general functional classes: pyrophosphohydrolases, adenine/guanine mismatch‐specific adenine glycosylases (A/G‐specific adenine glycosylases), isopentenyl diphosphate isomerases (IDIs), and proteins with nonenzymatic activities such as protein interaction and transcriptional regulation. Despite this degree of functional divergence across the clan, all of the 78 structurally characterized clan members (see Materials and Methods) contain a characteristic ∼130 amino acid beta‐grasp domain architecture^2^ (Fig. 1) classified as the Nudix fold (SCOPe v2.03 sunid 55810, sccsid d.113).^3^, ^4^ The clan's name highlights the fact that initially characterized members are pyrophosphohydrolases that cleave substrates of the general structure “*nu*cleoside *di*phosphate linked to a variable moiety **X**” (see Table I for all abbreviations). Clan members that are not pyrophosphohydrolases still share an evolutionary relationship as evidenced by sequence and structural conservation.^3^, ^4^, ^5^, ^6^, ^7^, ^8^, ^9^, ^10^ In the Nudix literature, sometimes the term “Nudix superfamily” is used narrowly to encompass only proteins with this pyrophosphohydrolase activity and specificity. For clarity, we refer to such proteins as “Nudix hydrolases.” Most evolutionary classifications and terminology^3^, ^4^, ^8^, ^9^, ^10^ use the term Nudix superfamily to designate all homologous domains regardless of activity or substrate; this is by analogy with the immunoglobulin and globin superfamilies, whose members also take on a diversity of functional roles other than in the immune system and oxygen binding. It is precisely the Pfam clan CL0261 named “Nudix Superfamily” that we are primarily analyzing herein. However, in the Nudix literature, members beyond those with Nudix hydrolase activity have sometimes been termed the Nudix suprafamily, reviewed in McLennan A. (2006). To bridge these disparate nomenclatures, we term these proteins the “Nudix homology clan.” Similarly, Nudix homology domains designate any that are related to others in the Nudix homology clan, and Nudix homology proteins designate those with a Nudix homology domain, regardless of specificity or activity.

Most of the experimentally characterized Nudix hydrolases contain a characteristic *ca*. 23 amino acid Nudix box motif: generally GX_5_EX_7_REUXEEXGU where U is a bulky alphatic residue (such as leucine, isoleucine, or valine) and X is any amino acid.^11^, ^12^ This sequence motif forms a loop‐helix‐loop structure primarily involved in binding one or more metal cations that in turn, orient the diphosphate moiety present in all Nudix hydrolase substrates.^5^, ^11^, ^13^ While isopentenyl diphosphate isomerases and A/G‐specific adenine glycosylases differ in sequence within this motif (they lack the conserved residues and sequence length exhibited by Nudix hydrolases), the overall loop‐helix‐loop architecture persists [Fig. 1(b,c)]. For example, instead of the Nudix hydrolase motif sequence (GX_5_EX_7_REUXEEXGU), the human isopentenyl diphosphate isomerase 1 enzyme (UniProt (The UniProt Consortium 2012) Entry Name: IDI1_HUMAN) possesses SX_7_EX_14_RRLXAEXGI and the human A/G‐specific adenine glycosylase (UniProt Entry Name: MUTYH_HUMAN) contains VX_2_EX_11_QELXRWAGP, yet both sequences still form a loop‐helix‐loop structure [Fig. 1(b,c)].

*Escherichia coli* MutT, the prototypical Nudix pyrophosphohydrolase, was originally identified as vital in preventing the incorporation of 8‐oxo‐2′‐deoxyguanosine 5′‐triphosphate (8‐oxo‐dGTP) into synthesizing DNA strands. Because this mutagenic nucleotide can basepair with either adenine or cytidine, its incorporation can induce A:T → C:G transversions when basepaired with dA, or G:C → T:A transversions when basepaired with dC during the first round of DNA synthesis and the incorporated 8‐oxo‐dG subsequently basepairs with dA during the next round of DNA replication.^14^ The *k*_cat_/*K*_m_ value for the MutT‐catalyzed hydrolysis of 8‐oxo‐dGTP is 1000‐fold greater than that for dGTP. This enzyme cleaves the α‐β phosphoanhydride bond of 8‐oxo‐dGTP to yield pyrophosphate and 8‐oxo‐dGMP.^15^ This prevents the incorporation of the oxidized, mutagenic nucleotide into the genome, thus “sanitizing” the nucleotide pool.

**Figure 2:**
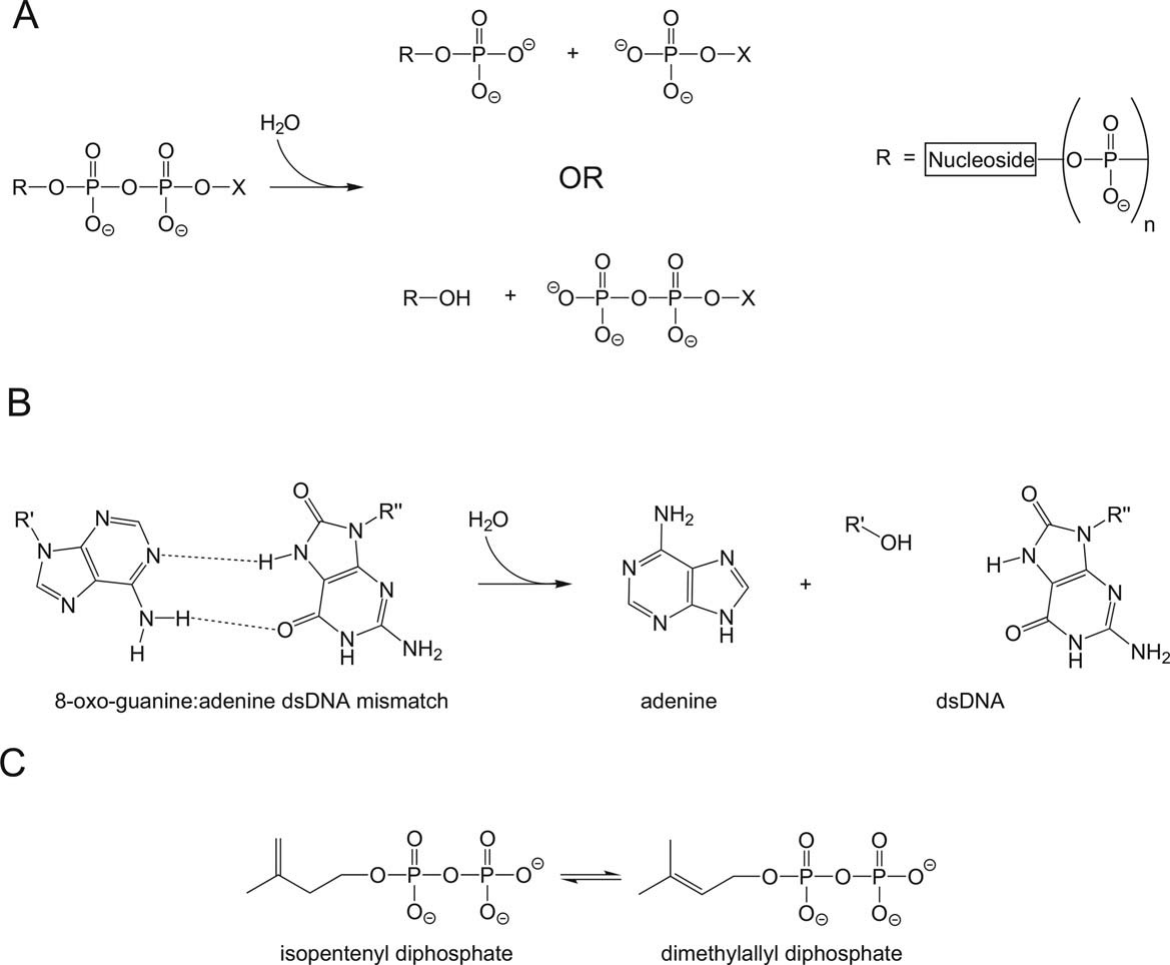
Reactions catalyzed by members of the Nudix homology clan. (**A**) Nudix hydrolase catalyzed reaction (predominantly EC 3.6.1.–). Canonical Nudix hydrolase substrates are nucleoside diphosphates linked to a variable moiety X. The nucleoside component (R) may include a variable number (*n*) of phosphate groups beyond the diphosphate, *n* = 0–4. Note that *n* may be zero, resulting in one phosphorylated product (bottom; e.g., the *E. coli* GDP‐glycosyl hydrolase NudD cleaves GDP‐glucose to yield GDP and glucose—EC 3.2.1.17).^53^ Hydrolysis of the diphosphate linkage may also result in two phosphoryl‐containing products (top; e.g., human diadenosine tetraphosphate hydrolase *NUDT2* hydrolyzes Ap_4_A to yield AMP and ATP—EC 3.6.1.17).^51^ (**B**) Adenine glycosylase catalyzed reaction (EC 3.2.2.–). A/G‐specific adenine glycosylases specifically catalyze the hydrolysis of the adenine‐deoxyribose glycosidic bond from a DNA base‐pair mismatch between 8‐oxo‐guanine and adenine, thereby excising the base from dsDNA. (**C**) Isopentenyl diphosphate isomerase (IDI) catalyzed reaction (EC 5.3.3.2). IDIs promote the interconversion between isopentenyl diphosphate and dimethylallyl diphosphate.

While additional Nudix hydrolases perform a “sanitizing” effect on cellular nucleotide pools,^16^ many additional Nudix hydrolase activities^5^ have been described since the discovery of MutT, broadening the potential cellular roles of this enzyme family.^6^ For example, pyrophosphohydrolases are now known to cleave ADP‐ribose (yielding adenosine 5′‐monophosphate and ribose 5‐phosphate).^17^ Recent studies indicate a broad swath of cellular roles for ADP‐ribose, including chromatin remodeling,^18^ membrane protein ion channel gating,^19^, ^20^ and a host of processes dependent upon ADP‐ribosylation;^17^, ^21^, ^22^ this suggests that Nudix ADP‐ribose pyrophosphohydrolases may play roles in many physiological contexts. Other hydrolases are involved in eukaryotic and bacterial mRNA decapping by recognizing either the 5′‐7‐methylguanosine or the NAD mRNA cap, respectively, initiating the process of mRNA degradation.^23^, ^24^, ^25^ Diadenosine polyphosphates (Ap_n_As) are structurally related hydrolase substrates implicated in modulating an alarmone response upon pathogen infection or other cellular stress events.^26^, ^27^ Hydrolyzing these metabolites potentially diminishes the effect of a host stress‐response, a feature for which pathogenic bacteria use Nudix hydrolases to their advantage.^28^, ^29^, ^30^ On the other hand, some plants employ Nudix hydrolases to mitigate pathogen infection and boost host immunity via activity on a variety of substrates.^31^, ^32^, ^33^, ^34^, ^35^ Utilization of Nudix hydrolases by invasive species is not limited to plants: the animal parasite *Trichinella spiralis* was recently found to be critically dependent upon the broad‐specificity pyrophosphohydrolase TsNd.^36^ Diphosphoinositol polyphosphates, another substrate set hydrolyzed by Nudix proteins, are known effectors of cell signaling, supporting a role for Nudix hydrolases in regulating the traffic of information within and between cells.^37^ Enzyme‐catalyzed hydrolysis among Nudix hydrolases usually occurs at a phosphorus atom participating in a pyrophosphate linkage, although there are enzymes that perform nucleophilic substitution at the carbon atom of some sugars (e.g., GDP‐sugar glycosyl hydrolases).^38^ Furthermore, some Nudix hydrolases contain additional and distinct protein domains that perform other enzymatic functions.^39^ Nudix protein hydrolase activity thus results in either one or two phosphorylated products [Fig. 2(a)].

In addition to those studies discussed above, other investigations into the propagation of A:T → C:G transversions led to the identification of *E. coli* MutY, the prototypical member of the Nudix homology protein family of A/G‐specific adenine glycosylases.^40^ MutY was characterized as a base‐excision repair enzyme that specifically recognizes dA/8‐oxo‐dG DNA base pair mismatches and removes the adenine base^41^ [Fig. 2(b)]. MutY activity does not directly correct the source of mutagenesis, but quarantines its mutagenic effect, thus ensuring DNA fidelity. In *E. coli*, the direct correction of incorporated 8‐oxo‐dG is mediated through the non‐Nudix protein MutM, which specifically excises the oxidatively‐damaged base.^14^ It is intriguing that two very different approaches (NTP hydrolysis and base excision) to suppress mutagenesis have evolved and both solutions depend upon members of the Nudix homology clan.

The third major category of Nudix homology proteins is the group of isopentenyl diphosphate isomerases (IDI). Rather than contributing to cellular sanitation, these enzymes play an important role in sterol metabolism by mediating the interconversion of isopentenyl diphosphate and dimethylallyl diphosphate [Fig. 2(c)]. The latter substrate is subsequently processed during *de novo* steroid biosynthesis.^42^ Superficially, the IDI‐catalyzed reaction seems unrelated to those catalyzed by Nudix hydrolases, but there are common mechanistic features between them. Both enzymes associate with the pyrophosphate moiety of their respective substrate through divalent metal ligation and subsequent catalysis involves general base mediated abstraction of a proton from either the substrate (isomerases) or from a nucleophilic water molecule (hydrolases).^5^

Finally, several Nudix homology proteins perform nonenzymatic activities. For example, the DBC1 protein family (PF14443) contains noncatalytic Nudix homology domains and is predicted to bind nicotinamide adenine dinucleotide (NAD) metabolites and regulate the activity of SIRT1 or related deacetylases.^43^, ^44^, ^45^ Transcriptional regulation^46^ and calcium channel gating^47^ activities were also reported for Nudix homology proteins. Noncatalytic Nudix homology domains are typically part of a multidomain protein and bind small molecules or interact with other protein domains.^20^, ^39^

It is challenging to assign specific function to an uncharacterized member of this clan. Nudix pyrophosphohydrolases have been characterized for an enormous diversity of functions, including activity on capped RNA,^23^, ^48^ (deoxy)nucleoside di‐ and triphosphates,^49^, ^50^ Ap_n_As,^29^, ^51^ NDP‐sugars,^52^, ^53^ and coenzymes such as thiamin pyrophosphate, CoA, and NADH.^54^, ^55^, ^56^ Furthermore, amino acid identity below 20% between most Nudix homology domains confounds the ability of traditional automated methods of function annotation.

In this article, we present an extensive functional assignment and phylogenetic analysis of the Nudix homology clan. First, through extensive manual curation of the literature, we gathered experimental and structural information for a total of 205 Nudix homology proteins. Our literature search led to a reevaluation of the current GO hierarchy related to the annotations of Nudix homology proteins. Here we propose the creation of new GO terms and rearrangement of the current hierarchy to more accurately reflect published Nudix homology protein characterizations. Second, due to the large degree of sequence divergence across the entire clan, alignment of enzymes with Nudix homology domains is significantly improved when guided by structural alignments because structure evolves far more slowly than sequence.^57^, ^58^ From this structural investigation, we present a loop region bordering the active site as a potential basis for the evolution of function among members of the Nudix homology clan with hydrolase activity. Finally, the few investigations to date into the evolution of function among the Nudix homology proteins typically focused on a single function or subfamily (such as Ap_n_A, diphosphoinositol polyphosphate, and ADP‐ribose hydrolases) and relied upon alignments generated via conventional automated algorithms.^59^, ^60^, ^61^, ^62^ Here we present the first cross‐functional phylogenetic analysis of the entire Nudix homology clan, an evolutionary investigation rooted in a manual structural alignment and deepened with extensive functional annotation of the clan members.

## MATERIALS AND METHODS

### Nudix data collection

We collected 192 publications characterizing Nudix homology proteins by searching PubMed with the keyword “Nudix” (as of July 2013; 51 more were published by August 2015). Each protein described in a publication was mapped to its UniProt Entry Name for a more precise identification. We built a MySQL database (supporting information Table S1, Resources 1 and 2) to store the experimental data (such as kinetic constants, relative activities, and descriptive results of genetic experiments), and bibliographical reference (in DOI or PubMed ID format). The experimental data are linked to proposed functions that are defined by current, modified, and proposed Gene Ontology terms (supporting information Fig. S2 and Tables 2 and 3).'

**Table 1:** List of Acronyms

Acronym	Name
2‐OH‐dATP	2‐Hydroxy‐deoxyadenosine‐5′‐triphosphate
2′‐O‐Me‐ATP	2′‐O‐methyladenosine‐5′‐triphosphate
2′‐O‐Me‐CTP	2′‐O‐methylcytidine‐5′‐triphosphate
2′‐O‐Me‐GTP	2′‐O‐methylguanosine‐5′‐triphosphate
2′‐O‐Me‐UTP	2′‐O‐methyluridine‐5′‐triphosphate
5‐Me‐CTP	5‐Methylcytidine‐5′‐triphosphate
5‐Me‐dCTP	5‐Methyl‐2′deoxycytidine‐5′‐triphosphate
5‐MeOH‐dCTP	5‐Hydroxymethyl‐2′‐deoxycytidine‐5′‐triphosphate
5‐MeOH‐dUTP	5‐Hydroxymethyl‐2′‐deoxyuridine‐5′‐triphosphate
5‐Me‐UTP	5‐Methyluridine‐5′‐triphosphate
5‐OH‐dCTP	5‐Hydroxy‐2′‐deoxycytidine‐5′‐triphosphate
8‐oxo‐dATP	8‐Oxo‐2′‐deoxyadenosine‐5′‐triphosphate
8‐oxo‐dGTP	8‐Oxo‐2′‐deoxyguanosine‐5′‐triphosphate
8‐oxo‐GTP	8‐Oxoguanosine‐5′‐triphosphate
ADP‐glucose	Adenosine‐5′‐diphosphoglucose
ADP‐ribose	Adenosine 5′‐diphosphoribose
Ap_3_A	P^1^,P^3^‐Di(adenosine‐5′) triphosphate
Ap_4_A	P^1^,P^4^‐Di(adenosine‐5′) tetraphosphate
Ap_4_dT	P^1^(−5′‐Adenosyl)‐P^4^‐[5′‐(2′‐deoxy‐thymidyl)]‐tetraphosphate
Ap_4_G	P^1^(−5′‐Adenosyl)‐P^4^‐(5′‐guanosyl)‐tetrataphosphate
Ap_4_U	P^1^(−5′‐Adenosyl)‐P^4^‐(5′‐uridyl)‐tetraphosphate
Ap_5_A	P^1^,P^5^‐Di(adenosine‐5′) pentaphosphate
Ap_5_dT	P^1^(−5′‐Adenosyl)‐P^5^‐[5′‐(2′‐deoxy‐thymidyl)]‐pentaphosphate
Ap_5_G	P^1^(−5′‐Adenosyl)‐P^5^‐(5′‐guanosyl)‐pentaphosphate
Ap_5_U	P^1^(−5′‐Adenosyl)‐P^5^‐(5′‐uridyl)‐pentaphosphate
Ap_6_A	P^1^(−5′‐Adenosyl)‐P^6^‐(5′‐adenosyl)‐hexaphosphate
Ap_n_A	Diadenosine polyphosphate
CDP‐choline	Cytidine 5′‐diphosphocholine
CDP‐glycerol	Cytidine 5′‐diphosphoglycerol
CoA	Coenzyme A
Deamino‐NAD^+^	Nicotinamide hypoxanthine dinucleotide
DHNTP	Dihydroneopterin triphosphate
DHUTP	5,6‐Dihydrouridine‐5′‐triphosphate
DIPP	Diphosphoinositol polyphosphate pyrophosphohydrolases
dITP	2′‐Deoxyinosine‐5′‐triphosphate
GDP‐fucose	Guanosine 5′‐diphospho‐β‐l‐fucose
GDP‐glucose	Guanosine 5′‐diphosphoglucose
GDP‐mannose	Guanosine 5′‐diphospho‐d‐mannose
GO	Gene Ontology
GOA	Gene Ontology Annotation
Gp_2_G	P^1^(−5′‐guanosyl)‐P^2^‐(5′‐guanosyl)‐diphosphate
Gp_3_G	P^1^(−5′‐guanosyl)‐P^3^‐(5′‐guanosyl)‐triphosphate
Gp_4_G	P^1^(−5′‐guanosyl)‐P^4^‐(5′‐guanosyl)‐tetraphosphate
Gp_5_G	P^1^,P^5^‐di(guanosine‐5′) pentaphosphate
HMM	Hidden Markov model
IDI	Isopentenyl diphosphate isomerases
ITP	Inosine‐5′‐triphosphate
LUCA	Last Universal Common Ancestor
m^7^Gp_3_C	P^1^‐(5′‐7‐methyl‐guanosyl)‐P^3^‐(5′‐cytidyl)‐triphosphate
m^7^GP_5_G	P^1^‐(5′‐7‐methyl‐guanosyl)‐P^5^‐(5′‐guanosyl)‐pentaphosphate
mRNA	messenger ribonucleic acid
N^1^‐Me‐ATP	N^1^‐Methyladenosine‐5′‐triphosphate
N^1^‐Me‐GTP	N^1^‐Methylguanosine‐5′‐triphosphate
N^4^‐Me‐dCTP	N^4^‐Methyl‐2′‐deoxycytidine‐5′‐triphosphate
N^6^‐Me‐ATP	N^6^‐methyladenosine‐5′‐triphosphate
NAD^+^	Nicotinamide adenine dinucleotide (oxidized)
NADH	Nicotinamide adenine dinucleotide (reduced)
NADP^+^	Nicotinamide adenine dinucleotide phosphate (oxidized)
NADPH	Nicotinamide adenine dinucleotide phosphate (reduced)
NAADP^+^	Nicotinic acid adenine dinucleotide phosphate
NDP	Nucleoside diphosphate
NTP	Nucleoside triphosphate
Nudix	Nucleoside diphosphate linked to a variable moiety X
oxidized‐CoA	Coenzyme A, oxidized (CoA‐S‐S‐CoA)
p_4_G	Guanosine 5′‐tetraphosphate
PDB	Protein Data Bank
P_i_	Phosphate
ppGpp	Guanosine‐3′,5′‐Bisdiphosphate
PP_i_	Pyrophosphate
PRPP	5‐Phospho‐d‐ribose 1‐diphosphate
snoRNA	Small nucleolar ribonucleic acid
TDP‐glucose	Thymidine‐5′‐diphospho‐α‐d‐glucose
UDP‐acetylgalactosamine	Uridine 5′‐diphospho‐*N*‐acetylgalactosamine
UDP‐acetylglucosamine	Uridine 5′‐diphospho‐*N*‐acetylglucosamine
UDP‐galactose	Uridine 5′‐diphosphogalactose
UDP‐glucose	Uridine 5′‐diphosphoglucose
UDP‐glucuronic acid	Uridine 5′‐diphosphoglucuronic acid
XTP	Xanthosine‐5′‐triphosphate
HMP‐pp	4‐Amino‐2‐methyl‐5‐hydroxymethylpyrimidine pyrophosphate
DHNTP	7,8‐Dihydroneopterin triphosphate

**Table 2:** Proposed New GO Terms

ID^a^	Term name	Definition	Parent 1	Parent 2
A001	2‐Hydroxy‐adenine DNA N‐glycoslyase activity	Remove 2‐hydroxy‐adenine bases by cleaving the N‐C1′ glycosidic bond between the oxidized purine and the deoxyribose sugar	GO:0008534	
A002	2‐Hydroxy‐deoxyadenosine diphosphatase activity	2‐OH‐Dadp + H_2_O = 2‐OH‐dAMP + phosphate	GO:0097382	
A004	2‐Hydroxy‐deoxyadenosine triphosphate phosphatase activity (product undefined)	2‐OH‐dATP + H_2_O = undefined + undefined	AP001	
A005	8‐Oxo‐guanosine triphosphate phosphatase activity (product undefined)	8‐Oxo‐GTP + H_2_O = undefined + undefined	AP001	
A006	8‐Oxo‐guanosine triphosphatase activity (Pi yielding)	8‐Oxo‐GTP + H_2_O = 8‐oxo‐GDP + phosphate	GO:0017111	A005
A007	5‐Methyl‐deoxycytidine triphosphate pyrophosphatase activity (PPi yielding)	5‐Methyl‐dCTP + H_2_O = 5‐methyl‐dCMP + pyrophosphate	GO:0047429	A008
A008	5‐Methyl‐deoxycytidine triphosphate phosphatase activity (product undefined)	5‐Methyl‐dCTP + H_2_O = undefined + undefined	AP001	
A009	5‐Methyl‐uridine triphosphate phosphatase activity (product undefined)	5‐Methyl‐UTP + H_2_O = undefined + undefined	AP001	
A010	5‐Hydroxy‐cytidine triphosphate phosphatase activity (product undefined) (product undefined)	5‐OH‐CTP + H_2_O = undefined + undefined	AP001	
A011	5‐Hydroxy‐deoxycytidine triphosphate phosphatase activity (product undefined)	5‐OH‐dCTP + H_2_O = undefined + undefined	AP001	
A012	8‐Hydroxy‐adenosine triphosphate pyrophophatase activity (PPi yielding)	8‐OH‐ATP + H_2_O = 8‐OH‐AMP + pyrophosphate	GO:0047429	
A013	8‐Hydroxy‐deoxyadenosine triphosphate pyrophosphatase activity (PPi yielding)	8‐OH‐dATP + H_2_O = 8‐OH‐dAMP + pyrophosphate	GO:0047429	A014
A014	8‐Hydroxy‐deoxyadenosine triphosphate phosphatase activity (product undefined)	8‐OH‐dATP + H_2_O = undefined + undefined	AP001	
A015	8‐Oxo‐deoxyguanosine triphosphatase activity (Pi yielding)	8‐Oxo‐dGTP + H_2_O = 8‐oxo‐dGDP + phosphate	GO:0017111	A016
A016	8‐Oxo‐deoxyguanosine triphosphate phosphatase activity (product undefined)	8‐Oxo‐dGTP + H_2_O = undefined + undefined	AP001	
A017	Bis(5′‐adenosyl)‐diphosphatase activity	Ap_2_A + H_2_O = AMP + AMP	AP002	
A018	Bis(5′‐adenosyl)‐tetraphosphate phosphatase activity (product undefined)	Ap_4_A + H_2_O = undefined + undefined	GO:0008796	
A019	P_1_‐(5′‐Adenosyl)P_4_‐(5′‐cytidyl) tetraphosphate phosphatase activity (product undefined)	AP_4_C + H_2_O = undefined + undefined	GO:0008796	
A020	P_1_‐(5′‐Adenosyl)P_4_‐(5′‐guanosyl) tetraphosphatase activity (ADP yielding)	AP_4_G + H_2_O = ADP + GDP	A021	
A021	P_1_‐(5′‐Adenosyl)P_4_‐(5′‐guanosyl) tetraphosphate phosphatase activity (product undefined)	AP_4_G + H_2_O = undefined + undefined	GO:0008796	
A022	P_1_‐(5′‐Adenosyl)P_4_‐(5′‐uridyl) tetraphosphate phosphatase activity (product undefined)	AP_4_U + H_2_O = undefined + undefined	GO:0008796	
A023	Bis(5′‐denosyl)‐pentaphosphatase activity (ADP yielding)	Ap_5_A + H_2_O = ATP + ADP	AP005	
A024	Bis(5′‐Adenosyl)‐pentaphosphate phosphatase activity (product undefined)	Ap_5_A + H_2_O = undefined + undefined	AP005	
A025	P_1_‐(5′‐Adenosyl)P_5_‐(5′‐guanosyl) pentaphosphate phosphatase activity (product undefined)	AP_5_G + H_2_O = undefined + undefined	AP005	
A026	Bis(5′‐denosyl)‐hexaphosphate phosphatase activity (product undefined)	Ap_6_A + H_2_O = undefined + undefined	AP006	
A027	Bis(5′‐adenosyl)‐hexaphosphatase activity (ADP yielding)	Ap_6_A + H_2_O = p_4_A + ADP	A026	
A028	P_1_‐(5′‐Adenosyl)P_6_‐(5′‐guanosyl) hexaphosphate phosphatase activity (product undefined)	AP_6_G + H_2_O = undefined + undefined	AP006	
A029	Arabinofuranosylcytosine triphosphate phosphatase activity (product undefined)	ara‐CTP + H_2_O = undefined + undefined	AP001	
A030	Adenosine triphosphate phosphatase activity (product undefined)	ATP + H_2_O = undefined + undefined	AP001	
A031	CDP‐glucose diphosphatase activity	CDP‐glucose + H_2_O = CMP + glucose 1‐phosphate	AP007	
A032	CDP‐ribose diphosphatase activity	CDP‐ribose + H_2_O = CMP + ribose 5‐phosphate	AP007	
A034	Cytidine triphosphate phosphatase activity (product undefined)	CTP + H_2_O = undefined + undefined	AP001	
A035	Deoxyadenosine diphosphatase activity	dADP + H_2_O = dAMP + phosphate	GO:0097382	
A036	Fatty‐acid‐acyl‐CoA diphosphatase activity	Fatty acid acyl coenzyme A + H_2_O = 3′,5′‐ADP + CoA remainder	AP011	
A054	Choloyl‐CoA diphosphatase activity	Choloyl‐CoA + H_2_O = 3′,5′‐ADP + CoA remainder	AP011	
A055	Deoxyadenosine triphosphate phosphatase activity (product undefined)	dATP + H_2_O = undefined + undefined	AP001	
A056	Deoxycytidine diphosphatase activity	dCDP + H_2_O = dCMP + phosphate	GO:0097382	
A057	Deoxycytidine triphosphate phosphatase activity (product undefined)	dCTP + H_2_O = undefined + undefined	AP001	
A058	Deoxycytidine triphosphatase activity (stepwise)	dCTP + H_2_O = dCMP + phosphate	GO:0017111	A057
A059	Deoxyguanosine diphosphatase activity	dGDP + H_2_O = dGMP + phosphate	GO:0097382	
A060	Deoxyguanosine triphosphate phosphatase activity (product undefined)	dGTP + H_2_O = undefined + undefined	AP001	
A061	CTP pyrophosphatase activity (PPi yielding)	CTP + H_2_O = CMP + pyrophosphate	GO:0047429	A034
A062	Uridine triphosphate phosphatase activity (product undefined)	UTP + H_2_O = undefined + undefined	AP001	
A063	Deoxyuridine triphosphate phosphatase activity (product undefined)	dUTP + H_2_O = undefined + undefined	AP001	
A064	Deoxyuridine triphosphatase activity (stepwise)	dUTP + H_2_O = dUMP + phosphate	GO:0017111	A063
A065	m^7^G(5′)ppp‐mRNA diphosphatase activity (m^7^GDP yielding)	m^7^G‐ppp‐mRNA + H_2_O = 7‐methyl‐GDP + p‐mRNA	AP013	
A066	G(5′)ppp‐mRNA triphosphatase activity (GMP yielding)	G‐ppp‐mRNA + H_2_O = GMP + p‐mRNA	AP013	
A067	G(5′)ppp‐mRNA triphosphatase activity (GDP yielding)	G‐ppp‐mRNA + H_2_O = GDP + p‐mRNA	AP013	
A068	GDP‐beta‐fucose diphosphatase activity	GDP‐beta‐fucose + H_2_O = GMP + fucose 1‐phosphate	AP008	
A069	GDP‐fructose diphosphatase activity	GDP‐fructose + H_2_O = GMP + fructose 1‐phosphate	AP008	
A070	GDP‐glucose diphosphatase activity	GDP‐glucose + H_2_O = GMP + glucose 1‐phosphate	AP008	
A071	GDP‐ribose diphosphatase activity	GDP‐ribose + H_2_O = GMP + ribose 5‐phosphate	AP008	
A072	Bis(5′‐guanosyl)‐diphosphatase activity	Gp_2_G + H_2_O = GMP + GMP	AP002	
A073	Bis(5′‐guanosyl)‐triphosphatase activity	Gp_3_G + H_2_O = GDP + GMP	AP003	
A074	Bis(5′‐guanosyl)‐tetraphosphate phosphatase activity (product undefined)	GP_4_G + H_2_O = undefined + undefined	GO:0008796	
A075	Bis(5′‐guanosyl)‐pentaphosphate phosphatase activity (product undefined)	GP_5_G + H_2_O = undefined + undefined	AP005	
A076	Guanosine triphosphate phosphatase activity (product undefined)	GTP + H_2_O = undefined + undefined	AP001	
A077	Guanosine triphosphatase activity (stepwise)	GTP + H_2_O = GMP + phosphate	GO:0017111	A076
A079	IDP‐ribose diphosphatase activity	IDP‐ribose + H_2_O = IMP + ribose 5‐phosphate	AP014	
A080	Inosine triphosphate phosphatase activity (product undefined)	ITP + H_2_O = undefined + undefined	AP001	
A081	m^7^G(5′)ppp‐snoRNA triphosphatase activity (m^7^GDP yielding)	m^7^G‐ppp‐snoRNA + H_2_O = 7‐methyl‐GDP + p‐snoRNA	AP013	
A082	N^4^‐Methyl‐deoxycytidine triphosphate phosphatase activity (product undefined)	N^4^‐methyl‐dCTP + H_2_O = undefined + undefined	AP001	
A084	O‐Acetyl‐ADP‐ribose diphosphatase activity	O‐acetyl‐ADP‐ribose + H_2_O = AMP + acetyl‐ribose 5‐phosphate	GO:0019144	
A085	Oxothiamine‐diphosphatase activity	OxoThDP + H_2_O = oxoThMP + phosphate	GO:0016462	
A086	Oxythiamine‐diphosphatase activity	OxyThDP + H_2_O = oxyThMP + phosphate	GO:0016462	
A087	Thiamine triphosphate phosphatase activity (product undefined)	ThTP + H_2_O = undefined + undefined	GO:0016462	
A088	Adenosine tetraphosphate phosphatase activity (product undefined)	P_4_A + H_2_O = undefined + undefined	AP001	
A089	Adenosine tetraphosphatase activity (AMP yielding)	P_4_A + H_2_O = AMP + triphosphate	A088	
A090	Guanosine tetraphosphate phosphatase activity (product undefined)	P_4_G + H_2_O = undefined + undefined	AP001	
A091	Adenosine pentaphosphate phosphatase activity (product undefined)	P_5_A + H_2_O = undefined + undefined	AP001	
A094	Guanosine 3′,5′‐bis(diphosphate) diphosphate phosphatase activity (product undefined)	ppGpp + H_2_O = undefined + undefined	GO:0017110	
A095	Guanosine 3′,5′‐bis(diphosphate) diphosphatase activity (stepwise)	ppGpp + H_2_O = pGp + phosphate	A094	
A096	Malonyl‐CoA diphosphatase activity	malonyl‐CoA + H_2_O = 3′,5′‐ADP + CoA remainder	AP011	
A098	Trihydroxycoprostanoyl‐CoA diphosphatase activity	THCA‐CoA + H_2_O = 3′,5′‐ADP + CoA remainder	AP011	
A099	Thymidine triphosphate phosphatase activity (product undefined)	TTP + H_2_O = undefined + UNDEFINED	AP001	
A100	Thymidine triphosphatase activity (stepwise)	TTP + H_2_O = TMP + phosphate	GO:0017111	A099
A101	UDP‐galactosamine diphosphatase activity	UDP‐galactosamine + H_2_O = UMP + galactoasmine phosphate	GO:0008768	
A102	UDP‐galactose diphosphatase activity	UDP‐galactose + H_2_O = UMP + galactose 1‐phosphate	GO:0008768	
A103	UDP‐galacturonic acid diphosphatase activity	UDP‐galacturonic acid + H_2_O = UMP + phosphogalacturonic acid	GO:0008768	
A104	UDP‐glucosamine diphosphatase activity	UDP‐glucosamine + H_2_O = UMP + glucoasmine phosphate	GO:0008768	
A105	UDP‐glucose diphosphatase activity	UDP‐glucose + H_2_O = UMP + glucose 1‐phosphate	GO:0008768	
A106	UDP‐glucuronic acid diphosphatase activity	UDP‐glucuronic acid + H_2_O = UMP + phosphoglucuronic acid	GO:0008768	
A107	UDP‐hexanolamine diphosphatase activity	UDP‐hexanolamine + H_2_O = UMP + hexanolamine phosphate	GO:0016462	
A108	UDP‐mannose diphosphatase activity	UDP‐mannose + H_2_O = UMP + mannose 1‐phosphate	GO:0008768	
A109	UDP‐*N*‐acetyl‐galactosamine diphosphatase activity	UDP‐*N*‐acetyl‐galactosamine + H_2_O = UMP + *N*‐acetyl‐galactosamine phosphate	GO:0008768	
A110	UDP‐*N*‐acetyl‐glucosamine diphosphatase activity	UDP‐*N*‐acetyl‐glucosamine + H_2_O = UMP + *N*‐acetyl‐glucosamine phosphate	GO:0008768	
A111	UDP‐*N*‐acetyl‐muramic acid diphosphatase activity	UDP‐*N*‐acetyl‐muramic acid + H_2_O = UMP + phospho‐*N*‐acetyl‐muramic acid	GO:0008768	
A112	UDP‐*N*‐acetyl‐muramoyl‐l‐alanine diphosphatase activity	UDP‐*N*‐acetyl‐muramoyl‐l‐Ala + H_2_O = UMP + *N*‐acetyl‐muramoyl‐l‐Ala phosphate	GO:0016462	
A113	Uridine triphosphatase activity (stepwise)	UTP + H_2_O = UMP + phosphate	GO:0017111	A062
A114	3‐Methyl‐3‐hydroxyglutaryl‐CoA diphosphatase activity	3‐Hydroxymethylglutaryl‐CoA + H_2_O = 3′,5′‐ADP + 3‐hydroxymethylglutarylpantetheine 4′‐phosphate	AP011	
A115	Deamino‐NAD^+^ diphosphatase activity	Deamino‐NAD^+^ + H_2_O = AMP + deamino‐NMN^+^	AP012	
A116	TDP‐glucose diphosphatase activity	TDP‐glucose + H_2_O = TMP + glucose 1‐phosphate	AP009	
A117	CDP‐ethanolamine diphosphatase activity	CDP‐ethanolamine + H_2_O = CMP + phosphoethanolamine	GO:0016462	
A118	CoA‐disulfide diphosphatase activity	CoASSCoA + H_2_O = 3′,5′‐ADP + 4′‐phosphopantetheine CoA disulfide	AP011	
A119	2′‐Phospho‐ADP‐ribose diphosphatase activity	2′‐Phospho‐ADP‐ribose + H_2_O = 2′,5′‐ADP + ribose 5‐phosphate	GO:0019144	
A120	3′‐Dephospho‐CoA diphosphatase activity	3′‐Dephospho‐CoA + H_2_O = AMP + 4′‐phosphopantetheine	AP011	
A121	ADP‐mannose diphosphatase activity	ADP‐mannose + H_2_O = AMP + mannose 1‐phosphate	GO:0019144	
A122	CoA‐glutathione diphosphatase activity	CoA‐glutathione + H_2_O = 3′,5′‐ADP + glutathionylpantetheine 4′‐phosphate	AP011	
A125	Acetyl‐CoA diphosphatase activity	Acetyl‐CoA + H_2_O = 3′,5′‐ADP + acetylpantetheine 4′‐phosphate	AP011	
A126	NAADP^+^ diphosphatase activity	NAADP^+^ + H_2_O = 2′,5′‐ADP + deamino‐NMN^+^	AP012	
A127	CDP‐choline diphosphatase activity	CDP‐choline + H_2_O = CMP + phosphocoline	GO:0016462	
A128	Succinyl‐CoA diphosphatase activity	Succinyl‐CoAvH_2_O = 3′,5′‐ADP + succinylpantetheine 4′‐phosphate	AP011	
A129	Deamino‐NADH diphosphatase activity	deamino‐NADH + H_2_O = AMP + deamino‐NMNH	AP012	
A130	P_1_‐(5′‐Adenosyl)P_3_‐(5′‐guanosyl) triphosphate phosphatase activity (product undefined)	Ap_3_G + H_2_O = undefined + undefined	AP003	
A131	3′‐Amino‐3′‐dATP pyrophosphatase activity (PPi yielding)	3′‐Amino‐3′‐dATP + H_2_O = 3′‐amino‐3′‐dAMP + pyrophosphate	GO:0047429	
A132	3′‐Amino‐3′‐TTP pyrophosphatase activity (PPi yielding)	3′‐Amino‐3′‐dTTP + H_2_O = 3′‐amino‐3′‐dTMP + pyrophosphate	GO:0047429	
A133	Thymidine‐diphosphatase activity	TDP + H_2_O = TMP + phosphate	GO:0017110	
A134	NADP^+^ diphosphatase activity	NADP^+^ + H_2_O = 2′,5′‐ADP + NMN^+^	AP012	
A135	Bis(5′‐adenosyl)‐tetraphosphate phosphatase activity (AMP yielding)	Ap_4_A + H_2_O = AMP + ATP	A018	
A136	adenosine pentaphosphate phosphatase activity (ATP yielding)	P_5_A + H_2_O = ATP + PPi	A091	
A137	Bis(5′‐adenosyl)‐hexaphosphatase activity (ATP yielding)	Ap_6_A + H_2_O = ATP + ATP	A026	
AP001	Nucleoside‐polyphosphate phosphatase activity	Hydrolysis of nucleoside polyphosphate at one pyrophosphate bound	GO:0016462	
AP002	Dinucleoside‐diphosphate phosphatase activity	Hydrolysis of dinucleoside diphosphate into two nucleotide	GO:0004551	
AP003	Dinucleoside‐triphosphate phosphatase activity	Hydrolysis of dinucleoside triphosphate into two nucleotide	GO:0004551	
AP005	Dinucleoside‐pentaphosphate phosphatase activity	Hydrolysis of dinucleoside pentaphosphate into two nucleotide	GO:0004551	
AP006	Dinucleoside‐hexaphosphate phosphatase activity	Hydrolysis of dinucleoside hexaphosphate into two nucleotide	GO:0004551	
AP007	CDP‐sugar diphosphatase activity	CDP‐sugar + H_2_O = CMP + sugar 1‐phosphate	GO:0016462	
AP008	GDP‐sugar diphosphatase activity	GDP‐sugar + H_2_O = GMP + sugar 1‐phosphate	GO:0016462	
AP009	TDP‐sugar diphosphatase activity	TDP‐sugar + H_2_O = TMP + sugar 1‐phosphate	GO:0016462	
AP011	General coenzyme A diphosphatase activity	Hydrolysis of coenzyme A or its derivatives	GO:0016462	
AP012	General NAD diphosphatase activity	Hydrolysis of NAD^+^ or its derivatives	AP002	
AP013	RNA decapping activity	Hydrolysis of capped RNA into an uncapped RNA and a small molecule	GO:0016462	
AP014	IDP‐sugar diphosphatase activity	Hydrolysis of IDP‐sugar derivatives	GO:0016462	

a Newly proposed children terms or parent terms associated with experimental data begin with A (e.g., A001), while parent terms without direct experimental data begin with AP (e.g., AP001).

**Table 3:** Proposed Modification of Gene Ontology

Reason for proposal	GO ID	Current name^a^	Definition^a^	Proposed name/definition	Current parent^b^	Proposed parent	Additional parent
Misleading or imprecise name	GO:0004551	Nucleotide diphosphatase activity	Dinucleotide + H_2_O = 2 mononucleotides	Dinucleotide‐polyphosphate phosphatase activity			
Misleading or imprecise name, change parent	GO:0050072	m^7^G(5′)pppN diphosphatase activity	m^7^‐G‐ppp‐polynucleotide + H_2_O = m^7^GMP + polynucleotide	m^7^G(5′)ppp‐mRNA diphosphatase activity (m7GMP yielding)	Pyrophosphatase activity (GO:0016462)	RNA decapping activity (AP013)	
	GO:0052751	GDP‐mannose hydrolase activity	GDP‐mannose + H_2_O = GMP + mannose‐1‐P	GDP‐mannose diphosphatase activity	Pyrophosphatase activity (GO:0016462)	GDP‐sugar diphosphatase activity (AP008)	
More specific name (add product description)	GO:0004170	dUTP diphosphatase activity	dUTP + H_2_O = dUMP + PPi	dUTP diphosphatase activity (PPi yielding)			
	GO:0019177	Dihydroneopterin triphosphate pyrophosphohydrolase activity	Dihydroneopterin triphosphate + H_2_O = dihydroneopterin phosphate + PPi	Dihydroneopterin triphosphate pyrophosphohydrolase activity (PPi yielding)			
	GO:0035870	dITP diphosphatase activity	dITP + H_2_O = dIMP + PPi	dITP diphosphatase activity (PPi yielding)			
	GO:0036222	XTP diphosphatase activity	XTP + H_2_O = XMP + PPi	XTP diphosphatase activity (PPi yielding)			
	GO:0044713	2‐OH‐ATP pyrophosphatase activity	2‐OH‐ATP + H_2_O = 2‐OH‐AMP + PPi	2‐OH‐ATP pyrophosphatase activity (PPi yielding)			
More specific name and definition (different from GO:0044713)	GO:0044714	2‐OH‐(d)ATP pyroposphatase activity	2‐OH‐(d)ATP + H_2_O = 2‐OH‐(d)AMP + PPi	2‐OH‐dATP pyrophosphatase activity (PPi yielding); 2‐OH‐dATP + H_2_O = 2‐OH‐dAMP + PPi			
More specific name (add product description), change parent	GO:0004636	Phosphoribosyl‐ATP diphosphatase activity	1‐(5‐Phospho‐d‐ribosyl)‐ATP + H_2_O = 1‐(5‐phosphonatoribosyl)−5′‐AMP + PPi	Phosphoribosyl‐ATP diphosphatase activity (PPi yielding)	Pyrophosphatase activity (GO:0016462)	Nucleoside‐triphosphate diphosphatase activity (PPi yielding) (GO:0047429)	
	GO:0008894	Guanosine‐5′‐triphosphate,3′‐diphosphate diphosphatase activity	Guanosine 5′‐triphosphate,3′‐diphosphate + H_2_O = guanosine 5′‐diphosphate,3′‐diphosphate + Pi	Guanosine‐5′‐triphosphate,3′‐diphosphate diphosphatase activity (Pi yielding)	Pyrophosphatase activity (GO:0016462)	Nucleoside‐triphosphatase activity (Pi yielding) (GO:0017111)	
	GO:0017111	Nucleoside‐triphosphatase activity	A nucleoside triphosphate + H_2_O = nucleoside diphosphate + Pi	Nucleoside‐triphosphatase activity (Pi yielding)	Pyrophosphatase activity (GO:0016462)	Nucleoside‐polyphosphate hydrolase activity (AP001)	
	GO:0034431	Bis(5′‐adenosyl)‐hexaphosphatase activity	P_1_‐P_6_‐bis(5′‐adenosyl) hexaphosphate + H_2_O = AMP + p_4_A	Bis(5′‐adenosyl)‐hexaphosphatase activity (AMP yielding)	Dinucleotide diphosphatase activity (GO:0004551)	Bis(5′‐adenosyl)‐hexaphosphatase activity(A026)	
	GO:0034432	Bis(5′‐adenosyl)‐pentaphosphatase activity	P_1_‐P_6_‐bis(5′‐adenosyl) pentaphosphate + H_2_O = AMP + p_4_A	Bis(5′‐adenosyl)‐pentaphosphatase activity (AMP yielding)	Dinucleotide diphosphatase activity (GO:0004551)	Bis(5′‐adenosyl)‐pentaphosphatase activity(A024)	
	GO:0047429	Nucleoside‐triphosphate diphosphatase activity	A nucleoside triphosphate + H_2_O = a nucleotide + PPi	Nucleoside‐triphosphate diphosphatase activity (PPi yielding)	Pyrophosphatase activity (GO:0016462)	Nucleoside‐polyphosphate hydrolase activity (AP001)	
	GO:0047624	Adenosine‐tetraphosphatase activity	p_4_A + H_2_O = ATP + Pi	Adenosine tetraphosphatase activity (ATP yielding)	Pyrophosphatase activity (GO:0016462)	Adenosine 5′‐tetraphosphatase activity (A088)	
	GO:0050333	Tthiamine‐triphosphatase activity	Thiamine triphosphate + H_2_O = thiamine diphosphate + Pi	Thiamine‐triphosphatase activity (Pi yielding)	Nucleoside‐triphosphatase activity (Pi yielding) (GO:0017111)	Thiamine triphosphate hydrolase activity (A087)	
More specific name (add product description), add parent	GO:0003924	GTPase activity	GTP + H_2_O = GDP + Pi	GTPase activity (Pi yielding)	Nucleoside‐triphosphatase activity (Pi yielding) (GO:0017111)		GTP phosphatase activity (product undefined) (A076)
	GO:0008413	8‐Oxo‐GTP pyrophosphatase activity	8‐oxo‐GTP + H_2_O = 8‐oxo‐GMP + PPi	8‐Oxo‐GTP pyrophosphatase activity (PPi yielding)	Nucleoside‐triphosphate diphosphatase activity (PPi yielding) (GO:0047429)		8‐oxo‐GTP phosphatase activity (product undefined) (A005)
	GO:0008828	dATP pyrophosphohydrolase activity	dATP + H_2_O = dAMP + PPi	dATP pyrophosphohydrolase activity (PPi yielding)	Nucleoside‐triphosphate diphosphatase activity (PPi yielding) (GO:0047429)		dATP phosphatase activity (product undefined) (A055)
	GO:0016887	ATPase activity	ATP + H_2_O = ADP + Pi	ATPase activity (Pi yielding)	Nucleoside‐triphosphatase activity (Pi yielding) (GO:0017111)		ATP phosphatase activity (product undefined) (A030)
	GO:0035539	8‐Oxo‐dGTP pyrophosphatase activity	8‐Oxo‐dGTP + H_2_O = 8‐oxo‐dGMP + PPi	8‐Oxo‐dGTP pyrophosphatase activity (PPi yielding)	Nucleoside‐triphosphate diphosphatase activity (PPi yielding) (GO:0047429)		8‐oxo‐dGTP phosphatase activity (product undefined) (A016)
	GO:0036217	dGTP diphosphatase activity	dGTP + H_2_O = dGMP + PPi	dGTP diphosphatase activity (PPi yielding)	Nucleoside‐triphosphate diphosphatase activity (PPi yielding) (GO:0047429)		dGTP phosphatase activity (product undefined) (A060)
	GO:0036218	dTTP diphosphatase activity	dTTP + H_2_O = dTMP + PPi	TTP diphosphatase activity (PPi yielding)	Nucleoside‐triphosphate diphosphatase activity (PPi yielding) (GO:0047429)		TTP phosphatase activity (product undefined) (A099)
	GO:0036219	GTP diphosphatase activity	GTP + H_2_O = GMP + PPi	GTP diphosphatase activity (PPi yielding)	Nucleoside‐triphosphate diphosphatase activity (PPi yielding) (GO:0047429)		GTP phosphatase activity (product undefined) (A076)
	GO:0036220	ITP diphosphatase activity	ITP + H_2_O = IMP + PPi	ITP diphosphatase activity (PPi yielding)	Nucleoside‐triphosphate diphosphatase activity (PPi yielding) (GO:0047429)		ITP phosphatase activity (product undefined) (A080)
	GO:0036221	UTP diphosphatase activity	UTP + H_2_O = UMP + PPi	UTP diphosphatase activity (PPi yielding)	Nucleoside‐triphosphate diphosphatase activity (PPi yielding) (GO:0047429)		UTP phosphatase activity (product undefined) (A062)
	GO:0043273	CTPase activity	CTP + H_2_O = CDP + Pi	CTPase activity (Pi yielding)	Nucleoside‐triphosphatase activity (Pi yielding) (GO:0017111)		CTP phosphatase activity (product undefined) (A034)
	GO:0047693	ATP diphosphatase activity	ATP + H_2_O = AMP + PPi	ATP diphosphatase activity (PPi yielding)	Nucleoside‐triphosphate diphosphatase activity (PPi yielding) (GO:0047429)		ATP phosphatase activity (product undefined) (A030)
	GO:0047840	dCTP diphosphatase activity	dCTP + H_2_O = dCMP + PPi	dCTP diphosphatase activity (PPi yielding)	Nucleoside‐triphosphate diphosphatase activity (PPi yielding) (GO:0047429)		dCTP phosphatase activity (product undefined) (A057)
	GO:0050339	Thymidine‐triphosphatase activity	TTP + H_2_O = TDP + Pi	TTPase activity (Pi yielding)	Nucleoside‐triphosphatase activity (Pi yielding) (GO:0017111)		TTP phosphatase activity (product undefined) (A099)
Change definition, change parent	GO:0004787	Thiamine‐pyrophosphatase activity	TDP + H_2_O = TMP + Pi	Thiamine‐diphosphate + H_2_O = thiamine‐phosphate + Pi	Nucleoside‐diphosphatase activity (GO:0017110)	Pyrophosphatase activity (GO:0016462)	
Change parent	GO:0000210	NAD^+^ diphosphatase activity	NAD^+^ + H_2_O = AMP + NMN^+^		Dinucleotide diphosphatase activity (GO:0004551)	general NAD diphosphatase activity (AP012)	
	GO:0008758	UDP‐2,3‐diacylglucosamine hydrolase activity	H_2_O + UDP‐2,3‐bis(3‐hydroxymyristoyl)glucosamine = 2,3‐bis(3‐hydroxymyristoyl)‐beta‐d‐glucosaminyl 1‐phosphate + UMP		Pyrophosphatase activity (GO:0016462)	UDP‐sugar diphosphatase activity (GO:0008768)	
	GO:0010943	NADPH pyrophosphatase activity	NADPH + H_2_O = NMNH + ADP		Dinucleotide diphosphatase activity (GO:0004551)	General NAD diphosphatase activity (AP012)	
	GO:0010945	CoA pyrophosphatase activity	Coenzyme A + H_2_O = 3′,5′‐ADP + 4′‐phosphopantetheine		Pyrophosphatase activity (GO:0016462)	General coenzyme A diphosphatase activity (AP011)	
	GO:0017110	Nucleoside‐diphosphatase activity	A nucleoside diphosphate + H_2_O = a nucleotide + Pi		Pyrophosphatase activity (GO:0016462)	Nucleoside‐polyphosphate hydrolase activity (AP001)	
	GO:0034353	RNA pyrophosphohydrolase activity	Catalysis of the removal of a 5′ terminal pyrophosphate from the 5′‐triphosphate end of an RNA, leaving a 5′‐monophosphate end.		Pyrophosphatase activity (GO:0016462)	RNA decapping activity (AP013)	
	GO:0035529	NADH pyrophosphatase activity	NADH + H_2_O = AMP + NMNH + 2 H^+^		Dinucleotide diphosphatase activity (GO:0004551)	General NAD diphosphatase activity (AP012)	
	GO:0044715	8‐Oxo‐dGDP phosphatase activity	8‐Oxo‐dGDP + H_2_O = 8‐oxo‐dGMP + Pi		Nucleoside‐diphosphatase activity (GO:0017110)	Deoxynucleoside‐diphosphatase activity (GO:0097382)	
	GO:0044717	8‐Hydroxy‐dADP phosphatase activity	8‐OH‐dADP + H_2_O = 8‐OH‐dAMP + Pi		Nucleoside‐diphosphatase activity (GO:0017110)	Deoxynucleoside‐diphosphatase activity (GO:0097382)	
	GO:0047884	FAD diphosphatase activity	FAD + H_2_O = AMP + FMN		Dinucleotide diphosphatase activity (GO:0004551)	Dinucleoside diphosphatase activity (AP002)	
	GO:0052840	Inositol diphosphate tetrakisphosphate diphosphatase activity	Inositol diphosphate tetrakisphosphate + H_2_O = inositol 1,3,4,5,6‐pentakisphosphate + Pi		Pyrophosphatase activity (GO:0016462)	Diphosphoinositol‐polyphosphate diphosphatase activity (GO:0008486)	
	GO:0052841	Inositol bisdiphosphate tetrakisphosphate diphosphatase activity	Inositol bisdiphosphate tetrakisphosphate + H_2_O = inositol diphosphate pentakisphosphate + Pi		Pyrophosphatase activity (GO:0016462)	Diphosphoinositol‐polyphosphate diphosphatase activity (GO:0008486)	
	GO:0052842	Inositol diphosphate pentakisphosphate diphosphatase activity	Inositol diphosphate pentakisphosphate + H_2_O = inositol hexakisphosphate + Pi		Pyrophosphatase activity (GO:0016462)	Diphosphoinositol‐polyphosphate diphosphatase activity (GO:0008486)	
	GO:0097382	Deoxynucleoside‐diphosphatase activity	A deoxynucleoside diphosphate + H_2_O = a deoxynucleotide + Pi		Pyrophosphatase activity (GO:0016462)	Nucleoside‐polyphosphate hydrolase activity (AP001)	
Remove (merge with GO:0080041)	GO:0047631	ADP‐ribose diphosphatase activity	ADP‐ribose + H_2_O = AMP + d‐ribose 5‐phosphate.				

a Names and definitions defined by Gene Ontology version 2014.01.01 are shown for all GO terms in the table.

b Parent of GO terms are omitted when the hierarchy of the term is not changed.

### Assigning confidence scores for nudix functions

We evaluated the reliability of a protein‐function assignment using confidence scores (*S*_final_) from zero to one. To compute *S*_final_, we first segregated experimental data associated with a protein‐function assignment into genetic data and biochemical data. This reflects the independence between biochemical and physiological measurements. From this, we can calculate a reasonable approximation of an overall confidence score for a specific activity:

$$S_{\text{overall}}=1-\left(1-S_{\text{genetic}}\right)\times \left(1-S_{\text{biochem}}\right)$$

Within each data category, genetic or biochemical, we further subcategorized the data types (see below) and assigned scores to each of them, and then took the maximum as the score of this category (*S*_genetic_ and *S*_biochem_) (Table 4). Finally, we adjusted the overall scores (*S*_overall_) based on the abundance of annotations for a given enzyme to obtain the final confidence score *S*_final_: if an enzyme has been assayed with a large number of substrates, the scores of the most active substrates would be tuned higher. A total of 2612 biochemical data elements and 63 genetic data were used to assign 939 protein‐functions pairs, each with a *S*_final_ value. All data collected from the literature as well as the temporary files used to generate the scores are provided as supplementary resources (supporting information Table S1, Resources 3–12).

**Table 4:** Confidence Score Assignment for Different Types of Experimental Evidence

Category		Evidence	Confidence score
Genetic		Phenotype shows the predicted physiology; in same species	0.99
		Phenotype relates to the predicted physiology; in same species	0.7
		Phenotype is inexplicable; in same species	0.1
		Complementation in different species	0.5
Biochemical	*k*_cat_/*K*_m_	10^7^ M^−1^ s^−1^	0.99
		10^6^ M^−1^ s^−1^	0.85
		10^5^ M^−1^ s^−1^	0.5
		10^4^ M^−1^ s^−1^	0.2
		10^3^ M^−1^ s^−1^	0.1
		0	0.01
	Pseudo *k*_cat_/*K*_m_	10^7^ M^−1^ s^−1^	0.7
		10^6^ M^−1^ s^−1^	0.5
		10^5^ M^−1^ s^−1^	0.25
		10^4^ M^−1^ s^−1^	0.1
		10^3^ M^−1^ s^−1^	0.05
		0	0.01
	Other Biochemical Data	100% Relative activity	0.1
		Gel electrophoresis for rare substrates	0.5
		Gel electrophoresis, HPLC for common substrates	0.05
		X‐ray structure with substrate for binding reaction	0.5
		X‐ray crystal structure with substrate	0.01
		Positive activity (*k*_cat_, *K*_m_, and first‐order rate constants only)	0.01

We subcategorized two types of data under the “genetic” category: knockout/knockdown and rescue (complementation tests). Knockout/knockdown data measure the physiologically relevant phenotypic change within the original species of the target protein, while rescue data are for such a change in a different species. For a phenotype of a knockout/knockdown that reflected the predicted physiology, related to the predicted physiology, or was inexplicable, *S*_knockout/knockdown_ were assigned values of 0.99, 0.7, and 0.1, respectively. An example of a predicted phenotype from a knockout experiment is that deletion of *nudB* from *E. coli*, which encodes for an enzyme that hydrolyzes DHNTP, the substrate of the committing step in folic acid synthesis, led to a marked reduction in folate synthesis, which was completely restored by a plasmid carrying the same gene.^63^ Lower confidence scores were given when the phenotype could only be considered as a related or inexplicable phenotype, but not as a direct effect of the knockout/knockdown. *S*_rescue_ was assigned to 0.7, as these experiments are often compelling. An example of a predicted phenotype from a rescue experiment is the expression of *mutT1* from *Mycobacterium tuberculosis* in *mutT* deficient *E. coli*. The *mutT1* rescued *E. coli* by reducing the A:T → C:G mutation rate.^64^ Finally, the maximum between *S*_knockout/knockdown_ and *S*_rescue_ was taken as the value of *S*_genetic_.

We subcategorized three types of data under the “biochemical” category: *k*_cat_/*K*_m_ values, substrate screening, and qualitative biochemical assays. The maximum score yielded from these biochemical characterization data was taken as the value of *S*_biochem_. Within this category, *k*_cat_/*K*_m_ values, when available, usually provided the most informative conclusion, and thus were assigned with the highest confidence scores. High *k*_cat_/*K*_m_ values (e.g., >10^6^ M^−1^ s^−1^) serve as a sufficient condition to indicate likely physiological substrates, while observation of low *k*_cat_/*K*_m_ values for Nudix hydrolases often means the investigated chemical is not likely the physiological substrate for the enzyme.^65^ Hence, we assigned scores of 0.99, 0.85, 0.5, 0.2, and 0.1, corresponding to *k*_cat_/*K*_m_ values 10^7^, 10^6^, 10^5^, 10^4^, 10^3^ M^−1^ s^−1^, respectively. The *k*_cat_/*K*_m_ values in between these intervals were given scores based on a log scale, so for example, a *k*_cat_/*K*_m_ value of 5 × 10^6^ M^−1^ s^−1^ was assigned with the confidence score of:

$$S_{k}{}_{\text{cat}/Km}=(({\text{log}}_{10}(5\;\times 10^{6})-{\text{log}}_{10}10^{6})\times \;\left(0.99-0.85\right))+0.85=0.948$$

Substrate screening data were all converted to relative activities, where the most active substrate was assigned to have 100% activity. Within the same substrate screening, if the *k*_cat_/*K*_m_ of one substrate, B, was determined elsewhere (possibly by other investigators), the “pseudo *k*_cat_/*K*_m_” of substrate A was calculated as follows:

$${\left(\text{pseudo}\;\;k_{\text{cat}}/K_{m}\right)}_{A}={\left[{\left(\text{relative}\;\text{activity}\right)}_{A}/{\left(\text{relative}\;\text{activity}\right)}_{B}\right]}^{2}\times {\left(k_{\text{cat}}/K_{m}\right)}_{B}$$

The square of the ratio of relative activities reflects our belief that *k*_cat_/*K*_m_ changes nonlinearly with relative activity, but is a crude approximation. Such pseudo *k*_cat_/*K*_m_ values, when available, were assigned with confidence scores in a similar way as the normal *k*_cat_/*K*_m_ values were assigned, but generally with lower scores (Table 4).

The remainder of screening data without any associated *k*_cat_/*K*_m_ data was given confidence scores linearly from 0 (0% relative activity) to 0.1 (100% relative activity). Other biochemical evidence, such as electrophoresis imaging, HPLC analysis of hydrolysis products, X‐ray crystallography structures with substrate‐analog binding, and positive activity data (*k*_cat_, *K*_m_, first‐order rate constants only) were considered as evidence to merely show that an enzyme is reactive to a compound, and thus were assigned a score of 0.01 (for HPLC data) or 0.05 (for X‐ray data). The only exception is such qualitative data with a substrate that is rarely shown to be active in literature, for example, electrophoresis imaging of RNA, which was given a score of 0.5, to reflect our belief that such activity is less likely to be a false positive, compared to the activity of a commonly screened substrate like 8‐oxo‐dGTP.

The overall confidence score for a specific activity, *S*_overall_ was adjusted to yield *S*_final_ for that activity so that when an enzyme has been annotated with many functions with low *S*_overall_ values, and with one “outlier” function with high *S*_overall_, the final confidence score for this outlier would be tuned even higher. For example, our confidence that a substrate with *k*_cat_/*K*_m_ = 10^5^ M^−1^ s^−1^ is a physiological substrate of an enzyme would be higher, if we know that this enzyme reacts moderately with numerous chemicals with *k*_cat_/*K*_m_ values in the range of 10^3^ M^−1^ s^−1^. To accomplish this, we first computed the *Z*‐scores of an enzyme, incorporating the distribution of *S*_overall_ values of all experimentally characterized functions assigned to this enzyme. The *Z*‐score of a given *S*_overall_, *Z*_s_, is computed as:

$$Z_{s}=\left(S_{\text{overall}}-〈S_{\text{overall}}〉\right)/{\text{SD}}_{\text{Soverall}}$$

where $〈S_{\text{overall}}〉$ is the average of *S*_overall_ values of all functions assigned to an enzyme, and $SD_{S_{\mathrm{overall}}}$ is the standard deviation of those values.

Next we adjusted the *S*_overall_ value of an enzyme‐function pair computed as:

$$S_{\text{final}}=1-(1-S_{\text{overall}})/(1+\left|Z_{s}\right|)\;[Z_{s}>0]$$

$$S_{\text{overall}}/(1+\left|Z_{s}\right|)\;\;\;\;\;\;\;\;[Z_{s}<0]$$

$$S_{\text{overall}}\;\;\;\;\;\;\;\;\;\;\;\;[Z_{s}=0]$$

### Revision of the gene ontology (GO) directed acyclic graph

We compared descendent GO terms from pyrophosphatase activity (GO:0016462) in the current GO database (release 2013–12‐07)^66^ to activities documented from the manual literature search. All relevant terms that were already in GO were reevaluated on the basis of position relative to other terms in the hierarchy, clarity in nomenclature and definition, and the ability to accurately describe published functions in the MySQL database. We created new terms for published functions with no corresponding GO term; an accurate term name, ontology, set of synonyms, and definition were assigned to these terms in the same manner as those already in GO (see Tables 2 and 3). Each new term was assigned an arbitrary number that started with “A” to distinguish it from terms currently in the database, or “AP” if it is a pure parent term without any direct experimental data.

### Aligning the structurally characterized nudix homology proteins

We searched UniProt release 2013‐04 for Nudix homology proteins that are in one of the Pfam families (PF00293, PF14443, PF09296, PF14815, PF13869) under the Pfam v27.0 (Mar 2013) Nudix clan (CL0261). We then retrieved PDB IDs for these proteins using the ID match function UniProt provides. The structures of 78 proteins were found in PDB release 2013‐02‐01. For proteins with multiple structures, or multiple chains (monomers) per structure, the selection criteria were (prioritized): (1) with substrate or substrate analog, (2) has higher resolution, and (3) has fewer missed residues. The selected structures (chains) were then trimmed to have only the Nudix homology domains in single chains, as indicated by Pfam (Table 5 and supporting information Fig. S1A). Structural alignments were visualized with PyMOL v0.99,^67^ Rasmol v2.7.1.1,^68^ and Chimera v1.6.2.^69^ Sequence alignments were visualized and edited using Jalview v2.8.^70^

**Table 5:** Nudix Homology Domain Structures in the 78‐PDB Structural and Sequence Alignments

Molecular Function	UniProt AC	PDB	Chain	Species	Reference
2‐OH‐dATP pyrophosphatase	P36639	3ZR0	A	*Homo sapiens*	116
3′‐5′ exonuclease	Q81EE8	3Q4I	A	*Bacillus cereus*	117
5‐Methyl‐dCTP pyrophosphatase	P77788	2RRK	A	*Escherichia coli*	(Kawasaki PDB ID: 2RRK)
8‐Oxo‐GDP pyrophosphatase	Q6ZVK8	3GG6	A	*Homo sapiens*	(Tresaugues PDB ID: 3GG6)
8‐Oxo‐GTP pyrophosphatase	P08337	3A6T	A	*Escherichia coli*	118
A:OxoG glycosylase	P83847	1RRS	A	*Geobacillus stearothermophilus*	93
A:OxoG glycosylase	Q9UIF7	1X51	A	*Homo sapiens*	(Tomizawa PDB ID: 1X51)
ADP‐ribose pyrophosphatase	O33199	1MQW	A	*Mycobacterium tuberculosis*	99
ADP‐ribose pyrophosphatase	O95848	3Q91	A	*Homo sapiens*	(Tresaugues PDB ID: 3Q91)
ADP‐ribose pyrophosphatase	Q55928	2QJO	A	*Synechocystis sp*.	108
ADP‐ribose pyrophosphatase	Q5NHR1	2QJT	B	*Francisella tularensis*	108
ADP‐ribose pyrophosphatase	Q5SJY9	2YVP	A	*Thermus thermophilus*	119
ADP‐ribose pyrophosphatase	Q84CU3	1V8M	A	*Thermus thermophilus*	120
ADP‐ribose pyrophosphatase	Q93K97	1KHZ	A	*Escherichia coli*	105
ADP‐ribose pyrophosphatase	Q9BW91	1QVJ	A	*Homo sapiens*	121
ADP‐ribose pyrophosphatase	Q9UKK9	2DSC	A	*Homo sapiens*	122
Ap3A pyrophosphatase	P45799	1VHZ	A	*Escherichia coli*	98
Ap4A pyrophosphatase	O04841	1JKN	A	*Lupinus angustifolius*	123
Ap4A pyrophosphatase	P50583	3U53	A	*Homo sapiens*	124
Ap4A pyrophosphatase	Q9U2M7	1KTG	A	*Caenorhabditis elegans*	125
Ap6A pyrophosphatase	Q75UV1	1VC8	A	*Thermus thermophilus*	(Iwai PDB ID: 1VC8)
CDP pyrophosphatase	Q9RY71	2O5F	A	*Deinococcus radiodurans*	49
CoA pyrophosphatase	Q9RV46	1NQZ	A	*Deinococcus radiodurans*	99
dGTP pyrophosphatase	Q9RVK2	1SU2	A	*Deinococcus radiodurans*	126
DHNTP pyrophosphatase	P0AFC0	2O1C	A	*Escherichia coli*	63
FAD pyrophosphatase	Q9RSC1	2W4E	A	*Deinococcus radiodurans*	127
GDP‐mannose mannosyl hydrolase	P32056	1RYA	A	*Escherichia coli*	38
GDP‐mannose pyrophosphatase	P37128	1VIU	B	*Escherichia coli*	98
GDP‐mannose pyrophosphatase	Q6XQ58	2I8T	A	*Escherichia coli*	100
HMP‐pp pyrophosphatase	P0AEI6	3SHD	A	*Escherichia coli*	(Hong PDB ID: 3SHD)
IPP isomerase	Q13907	2ICK	A	*Homo sapiens*	95
IPP isomerase	Q46822	1PPV	A	*Escherichia coli*	97
IPP isomerase	Q9BXS1	2PNY	A	*Homo sapiens*	(Walker PDB ID: 2PNY)
mRNA binding	O43809	2J8Q	A	*Homo sapiens*	103
mRNA decapping enzyme	O13828	2QKM	B	*Schizosaccharomyces pombe*	128
mRNA decapping enzyme	P53550	2JVB	A	*Saccharomyces cerevisiae*	129
mRNA decapping enzyme	Q96DE0	2XSQ	A	*Homo sapiens*	(Tresaugues PDB ID: 2XSQ)
NADH pyrophosphatase	P32664	1VK6	A	*Escherichia coli*	(JCSG PDB ID: 1VK6)
PP‐InsP5 pyrophosphatase	O95989	2FVV	A	*Homo sapiens*	130
PP‐InsP5 pyrophosphatase	Q8NFP7	3MCF	A	*Homo sapiens*	(Tresaugues PDB ID: 3MCF)
RNA pyrophosphohydrolase	P0A776	2KDV	A	*Escherichia coli*	(Bi PDB ID: 2KDV)
RNA pyrophosphohydrolase	Q6MPX4	3FFU	A	*Bdellovibrio bacteriovorus*	131
snoRNA decapping enzyme	Q6TEC1	2A8T	A	*Xenopus laevis*	101
transcription factor	Q8EFJ3	3GZ8	A	*Shewanella oneidensis*	46
(Undetermined)	A0REX4	3SMD	A	*Bacillus thuringiensis*	(Palani PDB ID: 3SMD)
(Undetermined)	A0ZZM4	3FJY	A	*Bifidobacterium adolescentis*	(Palani PDB ID: 3FJY)
(Undetermined)	B9WTJ0	3O8S	A	*Streptococcus suis*	(JCSG PDB ID: 3O8S)
(Undetermined)	C3H476	3ID9	A	*Bacillus thuringiensis*	(Palani PDB ID: 3ID9)
(Undetermined)	C8WVE1	3QSJ	A	*Alicyclobacillus acidocaldarius*	(Michalska PDB ID: 3QSJ)
(Undetermined)	D4Q002	3SON	A	*Listeria monocytogenes*	(JCSG PDB ID: 3SON)
(Undetermined)	O66548	3I7V	A	*Aquifex aeolicus*	120
(Undetermined)	O67435	2YYH	A	*Aquifex aeolicus*	(Nakakaga PDB ID: 2YYH)
(Undetermined)	P53370	3H95	A	*Homo sapiens*	(Tresaugues PDB ID: 3H95)
(Undetermined)	P65556	2FKB	A	*Escherichia coli*	(Nocek PDB ID: 2FKB)
(Undetermined)	Q03S37	3EXQ	A	*Lactobacillus brevis*	(Palani PDB ID: 3EXQ)
(Undetermined)	Q0TS82	3F6A	A	*Clostridium perfringens*	(Palani PDB ID: 3F6A)
(Undetermined)	Q0TTC5	3FCM	A	*Clostridium perfringens*	(Palani PDB ID: 3FCM)
(Undetermined)	Q2RXE7	3R03	A	*Rhodospirillum rubrum*	(Zhang PDB ID: 3R03)
(Undetermined)	Q2RXX6	3DUP	A	*Rhodospirillum rubrum*	(Patskovsky PDB ID: 3DUP)
(Undetermined)	Q3JWU2	4DYW	A	*Burkholderia pseudomallei*	(Edwards PDB ID: 4DYW)
(Undetermined)	Q5LBB1	3GWY	A	*Bacteroides fragili*	(Patskovsky PDB ID: 3GWY)
(Undetermined)	Q6G5F4	3HHJ	A	*Bartonella henselae*	132
(Undetermined)	Q7NWQ3	3F13	A	*Chromobacterium violaceum*	(Bonanno PDB ID: 3F13)
(Undetermined)	Q82VD6	3CNG	A	*Nitrosomonas europaea*	(Osipiuk PDB ID: 3CNG)
(Undetermined)	Q82XR9	2B0V	A	*Nitrosomonas europaea*	(Osipiuk PDB ID: 2B0V)
(Undetermined)	Q830S2	2FML	A	*Enterococcus faecalis*	(Chang PDB ID: 2FML)
(Undetermined)	Q836H1	2AZW	A	*Enterococcus faecalis*	(Zhang PDB ID: 2AZW)
(Undetermined)	Q8AAV8	2FB1	A	*Bacteroides thetaiotaomicron*	(Chang PDB ID: 2FB1)
(Undetermined)	Q8PYE2	3GRN	A	*Methanosarcina mazei*	(Patskovsky PDB ID: 3GRN)
(Undetermined)	Q8R2U6	2DUK	A	*Mus musculus*	(Hosaka PDB ID: 2DUK)
(Undetermined)	Q8ZM82	3HYQ	A	*Salmonella typhimurium*	(Kim PDB ID: 3HYQ)
(Undetermined)	Q8ZNF5	3N77	A	*Salmonella typhimurium*	(Frydrysiak PDB ID: 3N77)
(Undetermined)	Q8ZTD8	1K2E	A	*Pyrobaculum aerophilum*	133
(Undetermined)	Q92EH0	3I9X	A	*Listeria innocua*	(Bonanno PDB ID: 3I9X)
(Undetermined)	Q97QH6	2B06	A	*Streptococcus pneumoniae*	(Zhang PDB ID: 2B06)
(Undetermined)	Q97T37	2PQV	A	*Streptococcus pneumoniae*	(Chang PDB ID: 2PQV)
(Undetermined)	Q9K704	3FK9	A	*Bacillus halodurans*	(Bonanno PDB ID: 3FK9)
(Undetermined)	Q9X1A2	3E57	A	*Thermotoga maritima*	(Choi PDB ID: 3E57)

For historical reasons, we first selected 46 out of 78 PDB structures and aligned them with five structural alignment programs: CE (version last modified July 16, 2008),^71^ DaliLite v3.1,^72^ MultiProt/STACCATO v1.0,^73^, ^74^ SSAP (accessed March 2008),^75^ and Structal (accessed March 2008).^76^ CE, DaliLite, and MultiProt/STACCATO were run locally. Alignments generated via SSAP and Structal were run on their servers, accessed at http://www.cathdb.info/cgi-bin/SsapServer.pl and http://molmovdb.mbb.yale.edu/align/, respectively. We then manually combined the results from these programs to generate a structure‐guided sequence alignment for 46 PDB structures. The resulting sequence alignment is denoted as the “46‐PDB alignment,” and was used for quality control in the downstream procedure.

Next, we used 3DCOMB v1.06^77^ to structurally align the 78 proteins with PDB entries, and to convert the resulting structural alignment to a preliminary sequence alignment (we denote this as the “3DCOMB alignment”). Based on the corresponding structurally aligned regions, we separated the sequence alignment into two parts: the well conserved portion where most sequences are aligned and the structures are well superimposed, and the less conserved portion where many gaps were present and the structures are not clearly superimposable. The 46‐PDB alignment was used to facilitate the definition of the well conserved portion, as well as to validate the alignment quality of the well‐conserved portion of the 3DCOMB alignment.

To curate the well‐conserved portion of the 3DCOMB alignment, we inspected the quality of side‐chain superimposition of protein residues in this portion, and adjusted the sequence alignment accordingly. Specifically, the C‐termini of the Nudix homology domains required substantial manual intervention to optimize the alignment, as they are structurally superimposable but not well conserved in sequence. To curate the less conserved part, we first clustered the structures into 19 subgroups based on DALI‐score similarity (by clustering PDB pairs with DALI scores ≥ 16), and then edited the alignment only within these DALI clusters. A DALI score threshold of 16 was chosen based on operational considerations (for example, to ensure structures were sufficiently similar for manual manipulation, and to adjust the sizes of the clusters to make them manually editable). The clustering was intended only to facilitate construction of the structure‐induced sequence alignment, rather than produce groups of proteins whose similarity has functional significance. During the alignment editing process, we used RAxML 7.3.8^78^ to build trees (see below) iteratively, and sorted the sequences based on their positions in the tree, to better visualize the alignment for manual editing. The 46‐PDB alignment was used in this step to validate and improve the quality of 3DCOMB alignment iteratively (supporting information Fig. S1A and Table S1, Resources 15 and 16). We denote the final curated alignment as the “78‐PDB alignment” (supporting information Table S1, Resource 17), which was used as a guide to align more sequences (see below).

### Aligning the 347 select nudix homology domains

We selected 340 Nudix homology proteins that match at least one of the following criteria: have a determined structure, have an experimentally characterized activity, or are included in the seed alignment of the Pfam Nudix family (v27.0; Pfam ID: PF00293) (supporting information Table S1, Resource 18). Seven proteins in this collection contain two Nudix homology domains in their sequence, thus in total we had 347 select Nudix homology domains to align. Seventy‐eight of these 347 Nudix homology domains were aligned in the 78‐PDB alignment described above. Of the remaining 269 Nudix homology domains (denoted as “246‐query domains” in supporting information Fig. S1B), we used the 78‐PDB alignment as a guide to align 246 sequences, resulting in a curated alignment of 324 (= 78 + 246) Nudix homology domains (see the next paragraph). We denote this alignment as the “324‐core alignment.” The last 23 (= 269–246) Nudix homology domains were collected after the construction of the 324‐core alignment; these 23 domains were aligned using the same procedure as all the other Nudix homology domains in the Nudix homology clan as described below; therefore, the alignment of the select 347 Nudix homology domains (denoted as the “347‐select alignment”) is a subset of the Nudix homology clan alignment (see the next section; supporting information Table S1, Resources 19–21).

We used the 78‐PDB alignment as a guide to align the 246‐query domains and curated the alignment iteratively. First, the potential domain regions of these proteins were mapped by running the hmmsearch function of HMMER 3.0^79^ on the HMM model of Pfam Nudix family (PF00293). We then used three different strategies to align these additional 246 domains:

1. The hmmalign function of HMMER 3.0. We used an HMM model built from the 78‐PDB alignment with the hmmbuild function of HMMER 3.0. All settings in the hmmbuild and hmmalign functions were set to default.
2. MAFFT v7.122^80^ with the “‐‐seed” option. Also we set the algorithm to be “‐‐localpair,” and end‐gap penalty to be “‐‐ep 0.9.” These two settings were remained the same for all MAFFT runs mentioned below.
3. BLAST on every of these 246 domains against the 78 structures. We used the top hits to classify these 246 sequences into the 19 DALI subgroups. We then ran MAFFT with the “‐‐seed” option for every subgroup.

The results from these three methods were combined together manually by: (1) comparing the alignment of the well conserved parts between the HMMER and the global MAFFT results; (2) comparing the alignment of the less conserved parts between the global MAFFT and subgroup MAFFT results. During the process, we iteratively built trees from the alignment and sorted the sequences for better visualizing and editing. We denote the resulting 324‐sequence alignment as the “324‐core alignment” (supporting information Table S1, Resource 19).

### Aligning the protein domains from the complete nudix homology clan

We aimed to create a full alignment of the complete Nudix homology clan in UniProt release 2013–04 (supporting information Fig. S1C). We used the 324‐core alignment as a template for the full alignment of the Nudix homology clan. To start, we ran the hmmsearch function of HMMER 3.0 with the HMM models of all five Pfam families under the Nudix homology clan, resulting in a collection of 80,616 domain sequences. By removing identical sequences and 119 proteins from undefined species, we identified 38,950 domain sequences. We then used the 324‐core alignment as a guide to align these 38,950 sequences. We could not use the “seed” option of MAFFT as it required too much memory. Instead, we applied a “divide‐and‐conquer” approach:

1. We built a rough alignment using the “‐‐alga ‐‐dpparttree ‐‐retree 2 ‐‐partsize 1000” options of MAFFT.
2. We used FastTree v2.1.7^81^ to build a guide tree out of this alignment.
3. We grouped the leaves (domain sequences) of the tree into 16 subgroups using a method derived from Prosperi's algorithm (see below);^82^ each subgroup has 583–5891 sequences.
4. For each subgroup, we used the “‐‐add” option of MAFFT to add the subgroup sequences to the 324‐core alignment. This resulted in 16 alignments, all of which had 324‐core sequences in common.
5. We combined these 16 subgroup alignments together, using their common 324‐core alignment as the guide (supporting information Fig. S1D). The regions unique to each subgroups were not aligned between subgroups.
6. We ran FastTree with the above combined alignment with the “‐pseudo 1.0 ‐gamma ‐spr 4 ‐mlacc 3 ‐slownni” options to build an accurate phylogenetic tree.
7. Finally, we attempted to cluster the leaves of the above tree, again using the same method as in Step 3, and iterated through Steps 4–7. We ran 29 iterations but did not see the iterations converge, as the topological similarity between trees from later iterations was comparable to the similarity between the trees of the first and last iterations. The phylogenetic analysis was thus performed under the tree from the first iteration, to minimize any potential errors introduced in later iterations. We denote the alignment result from the first iteration as the “Nudix‐clan alignment”.

A key step in the above procedure is to cluster the leaves of a tree into subgroups. Prosperi proposed an algorithm (2011) to partition a tree based on the distribution of all patristic distances between pairs of leaves (whole‐tree distribution) and the distribution of all pairwise patristic distances within any sub‐tree (sub‐tree distribution). A subtree is classified as a cluster if its mean distance is below a percentile threshold of the whole‐tree distribution. This method, despite its simplicity in implementation, has two drawbacks. First, the whole‐tree distribution consumes a large amount of memory and CPU time (O(N^2^)), which makes it difficult to apply to the Nudix phylogeny (38,950 leaves). Second, the method tends to generate small and fragmented clades, which, while capturing the characteristics of the tree, does not serve our purpose of building an accurate multiple sequence alignment.

We modified the method in two ways to overcome the above limitations. First, we approximated the patristic‐distance distribution by assuming all branches under a node are equal in length. Therefore, we were able to use mean distance and the number of leaves under a node to calculate the approximate contribution of this particular clade to the whole‐tree distribution. Accordingly, we used the mean instead of the median distance for the percentile threshold cutoff (which we set to 0.05). When applied to the Nudix phylogeny, this approximation partitioned the tree in seconds on a laptop, usually resulting in around 30 clades.

Second, we limited the clade size to be between 400 and 4000 leaves to reduce both the degree of fragmentation of the Nudix homology clan and the CPU time required to run MAFFT. We broke any large clade (those with >4000 leaves) produced from the first step above into two smaller clades by separating them from the root of the original clade. Next, we combined any small clade (those with fewer than 400 leaves) with its adjacent clade to form a larger clade, but only if the resulting clade would have 4000 leaves or fewer. If a small clade could not be combined with its adjacent clade, all the leaves within the clade would be marked as not clustered. This procedure usually resulted in around 15 clades and *ca*. 1000 leaves that were not clustered in an iteration. We grouped these 1000 leaves together and treated them as one clade in the downstream alignment procedure (supporting information Table S1, Resource 21).

### Phylogenetic reconstruction

The 347‐select alignment was used as the input for RAxML 7.3.8,^78^ with the settings “‐f d ‐p 870119 ‐m PROTGAMMALGF ‐N 100” for tree building (i.e., starting from 10 random initial trees), and “‐f d ‐x 840907 ‐p 870119 ‐m PROTGAMMALGF ‐N 1000” for bootstrapping (i.e., doing 1000 bootstrapping iterations).

The phylogeny of the complete Nudix homology clan alignment was built from FastTree v2.1.7,^81^ as a direct result of the pipeline described previously. Both trees were midpoint rooted using Dendroscope v3.2.8.^83^ We also attempted to root the tree using outgroup rooting with either A/G‐specific adenine glycosylase (Pfam ID: PF14815) or DBC1 (Pfam ID: PF14443), as both belong to different Pfam families from the Pfam Nudix family (Pfam ID: PF00293), but the same clan with Nudix in Pfam (Pfam ID: CL0261). The outgroup rooting generated the same or more duplication events compared to the midpoint rooting results, so midpoint rooting was chosen for consistency. To reconcile the trees, we first gathered species information of these proteins from UniProt release 2013‐04. We then mapped these species to iTOL version 2.2.2^84^ to get the species tree. Finally, we ran Forester v1.028^85^ to reconcile the trees (supporting information Table S1, Resources 22–25).

## RESULTS AND DISCUSSION

### Data sources and analysis

From an extensive review of the literature (192 papers as of July 2013, our collection cutoff), we catalogued 171 Nudix homology proteins that have been genetically or biochemically characterized for a total of 161 activities. The activity data were subclassified according to four categories: (1) genetic data, where activity was determined by phenotype observed from gene knockdown, knockout or complementation test; (2) kinetic data, where Michaelis‐Menten parameters were determined for at least one substrate; (3) relative activity data, where activity was determined for a number of substrates at a fixed concentration; and (4) binary biochemical data, where activity was determined by qualitative biochemical assays such as HPLC and electrophoresis.

We assigned confidence scores to every protein's function annotation collected from the literature (see “Materials and Methods” for details). We were motivated to develop these scores, so as to be able to compare functional characterizations made by disparate experimental studies. Our approach presented here is admittedly arbitrary given the fundamental challenges of comparing sparse and disparate data to make systematic conclusions regarding functional physiological activity. However, these metrics incorporate our judgment and experience to yield intuitively consistent descriptions of function assignment confidence. Briefly, high scores were assigned to annotations from reliable biochemical assays (that is, kinetic data with very high *k*_cat_/*K*_m_ values) or from strong genetic evidence, while low scores were assigned for those based only on qualitative biochemical assays (e.g., substrate screening). The confidence scores were also adjusted based on the distribution of such scores for a given protein. For example, if an enzyme has been tested on a large number of substrates with low activity, and a few substrates with markedly higher activity, the scores of the most active substrates would be tuned higher. In total, we assigned confidence scores for 932 protein function annotations (supporting information Table S1, Resource 5), in which 51 Nudix homology proteins have their best activities scoring >0.9, 82 Nudix homology proteins between 0.5 and 0.9, and the remaining 38 proteins only have scores <0.5. If the confidence score for a protein‐function annotation falls below 0.5, we interpreted this result as an unreliable function assignment. Specifically, we classified 586 annotations with scores below 0.2 as unlikely to be representative of physiological activity, and applied this criterion to curate the Gene Ontology Database (next paragraph). The confidence score assignments were visualized together with the proposed Nudix phylogeny (discussed later in this article).

### Reevaluation of the GO terms

The Gene Ontology (GO)^66^ is a systematic organization of descriptive terms that aids consistent functional classification. These terms are assigned to gene products in the associated Gene Ontology Annotation database (GOA).^86^, ^87^ Of the 161 Nudix functions described in the literature, only 23 can be described precisely by current GO terms (release 2014‐01‐01). Therefore, we propose a total of 123 new terms, including 111 terms to adequately describe all the experimentally verified functions, and 12 parent terms to reflect a more adequate hierarchy (Table 2 and supporting information Fig. S2). We further propose to change 47 current GO terms so that their name or definition is more precise, or their parent/child relationships are altered (Table 3 and supporting information Fig. S2). Finally, our manual curation and analysis of the Nudix hydrolase literature uncovered 97 Nudix function assignments by GOA (release 2013‐12‐11), out of which 53 are problematic; these include 27 inaccurate annotations, 14 annotations that were not sufficiently precise, and 23 annotations for which the experimental data were insufficient to be confident of physiological relevance of the assigned molecular function (Table 6 and supporting information Table S1, Resource 13; see the next section).

**Table 6:** Comparison between Gene Ontology Annotation (GOA) and Our Manual Annotation of Nudix Homology Proteins

Reason	UniProt name	GOA database annotation with evidence code^a^			Manual annotation with confidence score^b^			Reference
Activity not stated in reference	NUD12_MOUSE	GO:0000210	NAD+ diphosphatase activity	IDA	(NA)			54
	NUD12_MOUSE	GO:0035529	NADH pyrophosphatase activity	IDA	(NA)			54
Activity with wrong substrate assigned	NUD15_MOUSE	GO:0008413	8‐Oxo‐7,8‐dihydroguanosine triphosphate pyrophosphatase activity	IDA	GO:0035539	8‐Oxo‐dGTP pyrophosphatase activity (PPi yielding)	0.86	134
	PCD1_YEAST	GO:0008413	8‐Oxo‐7,8‐dihydroguanosine triphosphate pyrophosphatase activity	IDA	GO:0035539	8‐Oxo‐dGTP pyrophosphatase activity (PPi yielding)	0.16	135
	TNR3_SCHPO	GO:0044715	8‐Oxo‐dGDP phosphatase activity	IDA	GO:0035539	8‐Oxo‐dGTP pyrophosphatase activity (PPi yielding)	0.19	136
	YJ9J_YEAST	GO:0044715	8‐Oxo‐dGDP phosphatase activity	IDA	GO:0035539	8‐Oxo‐dGTP pyrophosphatase activity (PPi yielding)	0.07	136
	NUD11_ARATH	GO:0000210	NAD+ diphosphatase activity	IDA	GO:0035529	NADH pyrophosphatase activity	0.00	137
	NUDT5_MOUSE	GO:0017110	Nucleoside‐diphosphatase activity	IDA	GO:0080041	ADP‐ribose pyrophosphohydrolase activity	0.85	138
Activity with wrong products assigned	NDX8_CAEEL	GO:0003986	Acetyl‐CoA hydrolase activity	IDA	A125	Acetyl‐CoA diphosphatase activity	0.56	139
	NUDT7_MOUSE	GO:0003986	Acetyl‐CoA hydrolase activity	IDA	A125	Acetyl‐CoA diphosphatase activity	0.53	55
	NUD26_ARATH	GO:0034432	Bis(5′‐adenosyl)‐pentaphosphatase activity	IDA	A023	Bis(5′‐adenosyl)‐pentaphosphatase activity (ADP yielding)	0.95	88
	NUD27_ARATH	GO:0034432	Bis(5′‐adenosyl)‐pentaphosphatase activity	IDA	A023	Bis(5′‐adenosyl)‐pentaphosphatase activity (ADP yielding)	0.10	88
	AP4A_HUMAN	GO:0008803	Bis(5′‐nucleosyl)‐tetraphosphatase (symmetrical) activity	TAS	A135	Bis(5′‐adenosyl)‐tetraphosphate phosphatase activity (AMP yielding)	1.00	51, 140
	NUDI_ECOLI	GO:0047840	dCTP diphosphatase activity	IDA	A058	Deoxycytidine triphosphatase activity (stepwise)	0.40	141
	NUDI_ECOLI	GO:0004170	dUTP diphosphatase activity	IDA	A064	Deoxyuridine triphosphatase activity (stepwise)	0.76	141
	8ODP_HUMAN	GO:0003924	GTPase activity	TAS	GO:0036219	GTP diphosphatase activity (PPi yielding)	0.09	142
	NUD11_ARATH	GO:0008893	Guanosine‐3′,5′‐bis(diphosphate) 3′‐diphosphatase activity	IDA	A095	Guanosine 3′,5′‐bis(diphosphate) diphosphatase activity (stepwise)	0.08	143
	NUD15_ARATH	GO:0008893	Guanosine‐3′,5′‐bis(diphosphate) 3′‐diphosphatase activity	IDA	A095	Guanosine 3′,5′‐bis(diphosphate) diphosphatase activity (stepwise)	0.03	143
	NUD25_ARATH	GO:0008893	Guanosine‐3′,5′‐bis(diphosphate) 3′‐diphosphatase activity	IDA	A095	Guanosine 3′,5′‐bis(diphosphate) diphosphatase activity (stepwise)	0.07	143
	NUD26_ARATH	GO:0008893	Guanosine‐3′,5′‐bis(diphosphate) 3′‐diphosphatase activity	IDA	A095	Guanosine 3′,5′‐bis(diphosphate) diphosphatase activity (stepwise)	0.14	143
	NDX8_CAEEL	GO:0047994	Hydroxymethylglutaryl‐CoA hydrolase activity	IDA	A114	3‐Methyl‐3‐hydroxyglutaryl‐CoA diphosphatase activity	0.59	139
	DCP2_HUMAN	GO:0050072	m7G(5′)pppN diphosphatase activity	IDA	A065	m7G(5′)ppp‐mRNA diphosphatase activity (m7GDP yielding)	0.50	144
	DCP2_SCHPO	GO:0050072	m7G(5′)pppN diphosphatase activity	IDA	A065	m7G(5′)ppp‐mRNA diphosphatase activity (m7GDP yielding)	1.00	145
	DCP2_YEAST	GO:0050072	m7G(5′)pppN diphosphatase activity	IDA	A065	m7G(5′)ppp‐mRNA diphosphatase activity (m7GDP yielding)	1.00	23
	NUD16_HUMAN	GO:0050072	m7G(5′)pppN diphosphatase activity	IDA	A065	m7G(5′)ppp‐mRNA diphosphatase activity (m7GDP yielding)	0.50	48
	NUD16_XENLA	GO:0050072	m7G(5′)pppN diphosphatase activity	IDA	A081	m7G(5′)ppp‐snoRNA triphosphatase activity (m7GDP yielding)	1.00	48
	NDX8_CAEEL	GO:0004778	Succinyl‐CoA hydrolase activity	IDA	A128	Succinyl‐CoA diphosphatase activity	0.58	139
Activity not specific enough to reflect the data in reference	NUDE_ECOLI	GO:0019144	ADP‐sugar diphosphatase activity	IDA	GO:0080042	ADP‐glucose pyrophosphohydrolase activity	0.03	91
	NUDT5_HUMAN	GO:0019144	ADP‐sugar diphosphatase activity	IDA	GO:0080042	ADP‐glucose pyrophosphohydrolase activity	0.72	146
	AP4A_CAEEL	GO:0004081	Bis(5′‐nucleosyl)‐tetraphosphatase (asymmetrical) activity	IDA	A135	Bis(5′‐adenosyl)‐tetraphosphate phosphatase activity (AMP yielding)	0.98	59
	NUD25_ARATH	GO:0004081	Bis(5′‐nucleosyl)‐tetraphosphatase (asymmetrical) activity	IDA	A135	Bis(5′‐adenosyl)‐tetraphosphate phosphatase activity (AMP yielding)	0.56	147
	DDP1_YEAST	GO:0008486	Diphosphoinositol‐polyphosphate diphosphatase activity	IDA	GO:0052842	Inositol diphosphate pentakisphosphate diphosphatase activity	0.96	148
	NUDT3_HUMAN	GO:0008486	Diphosphoinositol‐polyphosphate diphosphatase activity	IDA	GO:0052842	Inositol diphosphate pentakisphosphate diphosphatase activity	1.00	149
	NUDT4_HUMAN	GO:0008486	Diphosphoinositol‐polyphosphate diphosphatase activity	IDA	GO:0052842	Inositol diphosphate pentakisphosphate diphosphatase activity	1.00	109
	Q7JVG2_DROME	GO:0008486	Diphosphoinositol‐polyphosphate diphosphatase activity	IDA	GO:0052842	Inositol diphosphate pentakisphosphate diphosphatase activity	0.98	150
	MUTY_ECOLI	GO:0016787	Hydrolase activity	IDA	GO:0034039	8‐Oxo‐7,8‐dihydroguanine DNA *N*‐glycosylase activity	1.00	41
	NUDG_ECOLI	GO:0016787	Hydrolase activity	IDA	A004	2‐Hydroxy‐deoxyadenosine triphosphate phosphatase activity (product undefined)	1.00	151
	Q5BE28_EMENI	GO:0016787	Hydrolase activity	IMP	GO:0000210	NAD+ diphosphatase activity	1.00	152
	Y079_DEIRA	GO:0016787	Hydrolase activity	IDA	GO:0047840	dCTP diphosphatase activity (PPi yielding)	0.63	49
	Q75UV1_THETH	GO:0016818	Hydrolase activity, acting on acid anhydrides, in phosphorus‐containing anhydrides	IDA	A137	Bis(5′‐adenosyl)‐hexaphosphatase activity (ATP yielding)	0.96	153
	NUD14_MOUSE	GO:0008768	UDP‐sugar diphosphatase activity	IDA	A105	UDP‐glucose diphosphatase activity	0.06	154
Activity not sufficiently supported by experimental study	AP4A_CAEEL	GO:0043135	5‐Phosphoribosyl 1‐pyrophosphate pyrophosphatase activity	IDA	GO:0043135	5‐Phosphoribosyl 1‐pyrophosphate pyrophosphatase activity	0.06	140
	NUD18_HUMAN	GO:0044717	8‐Hydroxy‐dADP phosphatase activity	IDA	GO:0044717	8‐Hydroxy‐dADP phosphatase activity	0.11	155
	NUD15_HUMAN	GO:0008413	8‐Oxo‐7,8‐dihydroguanosine triphosphate pyrophosphatase activity	IDA	GO:0008413	8‐Oxo‐GTP pyrophosphatase activity (PPi yielding)	0.01	155
	NUD10_ARATH	GO:0047631	ADP‐ribose diphosphatase activity	IDA	GO:0080041	ADP‐ribose pyrophosphohydrolase activity	0.13	156
	NUDT6_ARATH	GO:0047631	ADP‐ribose diphosphatase activity	IDA	GO:0080041	ADP‐ribose pyrophosphohydrolase activity	0.10	156
	8ODP_HUMAN	GO:0047693	ATP diphosphatase activity	IDA	GO:0047693	ATP diphosphatase activity (PPi yielding)	0.02	157
	DDP1_YEAST	GO:0034431	Bis(5′‐adenosyl)‐hexaphosphatase activity	IDA	GO:0034431	Bis(5′‐adenosyl)‐hexaphosphatase activity (AMP yielding)	0.12	158
	DDP1_YEAST	GO:0034432	Bis(5′‐adenosyl)‐pentaphosphatase activity	IDA	GO:0034432	Bis(5′‐adenosyl)‐pentaphosphatase activity (AMP yielding)	0.06	158
	NUD13_ARATH	GO:0034432	Bis(5′‐adenosyl)‐pentaphosphatase activity	IDA	GO:0034432	Bis(5′‐adenosyl)‐pentaphosphatase activity (AMP yielding)	0.08	88
	NUD11_ARATH	GO:0010945	CoA pyrophosphatase activity	IDA	GO:0010945	CoA pyrophosphatase activity	0.17	137
	NUD15_ARATH	GO:0010945	CoA pyrophosphatase activity	IDA	GO:0010945	CoA pyrophosphatase activity	0.11	88
	NUDT1_ARATH	GO:0019177	Dihydroneopterin triphosphate pyrophosphohydrolase activity	IDA	GO:0019177	Dihydroneopterin triphosphate pyrophosphohydrolase activity (PPi yielding)	0.02	159

a The GOA version is 2013.12.11. Only included are experimental GOA (evidence code: IDA, IMP, IGI, IPI, EXP, and TAS) that are not consistent with our manual annotation.

b The false positive annotations, whose confidence scores are <0.2, are indicated in gray.

Because the current set of Nudix‐related GO terms does not fully encompass all of the activities described in the literature, we propose a total of 111 new terms to describe experimentally verified Nudix functions. For example, despite the various types of reported nucleotide‐sugar diphosphatase activities, only ADP‐ribose pyrophosphohydrolase activity (GO:0080041), ADP‐glucose pyrophosphohydrolase activity (GO:0080042), GDP‐mannose diphosphatase activity (GO:0052751), and UDP‐sugar diphosphatase activity (GO:0008768) exist as GO terms. Therefore, we propose 23 new nucleotide‐sugar diphosphatase activities (e.g., UDP‐galactose diphosphatase activity (A102), CDP‐ribose diphosphatase activity (A032), and GDP‐fructose diphosphatase activity (A069)). Many other new terms are proposed in tandem with changes for current terms, as described below.

We changed the names of three and the definitions of two GO terms to resolve imprecise activity designations. First, nucleotide diphosphatase activity (GO:0004551) is defined in GO release 2013‐12‐07 as catalysis of the reaction: dinucleotide + H_2_O → 2 mononucleotides. To distinguish this reaction from a proposed (mono)nucleoside‐polyphosphate phosphatase activity (AP001), we renamed GO:0004551 to dinucleotide polyphosphate phosphatase activity. Second, GDP‐mannose hydrolase activity (GO:0052751) is currently defined as catalysis of the reaction: GDP‐mannose + H_2_O → GMP + mannose 1‐phosphate. We renamed this term to GDP‐mannose diphosphatase activity to highlight the fact that it cleaves the pyrophosphate bond and not the glycosidic bond. (The reaction GDP‐mannose + H_2_O →  GDP + mannose is defined in another GO term, GDP‐mannose mannosyl hydrolase activity (GO:0008727).) Third, m^7^G(5′)pppN diphosphatase activity (GO:0050072) was renamed to m^7^G(5′)ppp‐mRNA diphosphatase activity (m7GMP yielding) to reflect the update in the Enzyme Commission name linked to the GO term (EC 3.6.1.59). Fourth, thiamine‐pyrophosphatase activity (GO:0004787) is defined as catalysis of the reaction: TDP + H_2_O → TMP + phosphate. However, the abbreviation TDP is commonly used for thymidine diphosphate. To avoid confusion, we changed the definition to thiamin‐diphosphate + H_2_O → thiamin‐monophosphate + phosphate (a new term, thymidine‐diphosphatase activity (A0133) was created for the reaction TDP + H_2_O → TMP + phosphate). Finally, we proposed a more specific name and definition of GO:0044714 to describe only the pyrophosphatase activity on 2‐OH‐dATP, but not on 2‐OH‐ATP, as the latter has been described by another GO term (GO:0044713).

We added product descriptions to the names of 28 current terms to precisely describe the reactions. For example, 8‐oxo‐7,8‐dihydroguanosine triphosphate pyrophosphatase activity (GO:0008413) is defined as catalysis of the reaction: 8‐oxo‐GTP + H_2_O → 8‐oxo‐GMP + diphosphate. There are no other terms available in GO that describe the hydrolysis of 8‐oxo‐GTP that yields 8‐oxo‐GDP + phosphate, even though this activity is also reported in the literature^64^. This motivated us to rename the term to include the product in its name: 8‐oxo‐7,8‐dihydroguanosine triphosphate pyrophosphatase activity (PPi yielding). Accordingly, we introduced a new sibling term, 8‐oxo‐guanosine triphosphatase activity (Pi yielding) (A006), to account for the other verified 8‐oxo‐GTP hydrolysis reaction.

For 14 existing GO terms, we added secondary parent terms to describe more generic reactions. For example, a majority of the reported activities for 8‐oxo‐GTP do not provide information on the substrate cleavage pattern.^88^ To accommodate this lack of information, we proposed a new general term for 8‐oxo‐GTP hydrolysis: 8‐oxo‐guanosine triphosphate phosphatase activity (A005). This term is a parent for both reactions of 8‐oxo‐GTP hydrolysis to yield either PPi (GO:0008413) or Pi (A006). Thus, now the 8‐oxo‐7,8‐dihydroguanosine triphosphate pyrophosphatase activity (PPi yielding) (GO:0008413) has two parent terms: the original nucleoside‐triphosphate diphosphatase activity (PPi yielding) (GO:0047429), and 8‐oxo‐guanosine triphosphate phosphatase activity (A005).

We repositioned 26 existing GO terms by grouping structurally related substrates as sibling terms, usually under a new or existing general term, in order to better represent the closely related activities in the GO hierarchy. For example, m^7^G(5′)ppp‐mRNA diphosphatase activity (m^7^GMP yielding) (GO:0050072) and RNA pyrophosphohydrolase activity (GO:0034353) are currently children terms of pyrophosphatase activity (GO:0016462), which is a wide term that encompasses all the possible hydrolysis specificities (e.g., ADP‐ribose, PP‐InsP_5_, mutagenic NTPs, and so forth) exhibited by Nudix hydrolases. The substrates defined in these two terms are structurally more similar to each other than they are to those in other terms, as they differ only by the 5′ cap. Furthermore, the similarity of these two terms is underscored by the fact that RPPH_ECOLI exhibits both m^7^G(5′)ppp‐mRNA diphosphatase^89^ and RNA pyrophosphohydrolase^90^ activities simultaneously. Therefore, we grouped these two terms (and four additional proposed terms involved in RNA hydrolysis) together as children to a newly created parent term, mRNA decapping activity (AP013).

We removed one term, ADP‐ribose diphosphatase activity (GO:0047631; defined as catalysis of the reaction: ADP‐ribose + H_2_O → AMP + d‐ribose 5‐phosphate), because it is redundant with another term, ADP‐rbose pyrophosphohydrolase activity (GO:0080041), which has the exact same definition. We also removed the synonyms for one term, NAD^+^ diphosphatase activity (GO:0000210), so that it refers more precisely to a specific reaction, while providing new terms to describe the removed synonyms as they do not yet exist. NAD^+^ diphosphatase activity (GO:0000210) is defined in GO as catalysis of the reaction: NAD^+ ^+ H_2_O → AMP + oxidized nicotinamide mononucleotide, yet its synonyms also describe the corresponding reactions on NADH and NADP^+^. However, some enzymes display different specificities for these substrates; for example, NUD12_HUMAN shows nearly 100‐fold higher catalytic activity toward NADH over NAD^+^.^91^ To annotate these different substrate specificities precisely, the synonyms, NADH and NADP^+^ diphosphatase activities, were removed from GO:0000210, and a separate term was created to describe the hydrolysis of NADP^+^ (A134) (NADH (GO:0035529), and NADPH (GO:0010943) pyrophosphatase activities already exist). In addition, NAADP^+^ (A126), deamino‐NAD^+^ (A115), and deamino‐NADH (A129) hydrolases activities are also proposed under the same parent term general NADH activity (AP012).

### Comparison between GOA and nudix literature

Upon examining the 97 annotations of 65 enzymatically characterized Nudix homology proteins in the Gene Ontology Annotation (GOA) database (release 2013‐12‐11), there appear to be cases of incorrect and uninformatively generic functional assignment. Of the 171 experimentally characterized Nudix enzymes we catalogued from the literature, only 25 had correct molecular function GO annotations with experimental evidence codes (usually IDA—inferred from direct assay) in GOA when compared with published biochemical data. Furthermore, we found 53 erroneous, imprecise, or insufficiently supported annotations in GOA (with experimental evidence codes) for 40 Nudix homology proteins (Table 6). Additionally, 106 experimentally characterized Nudix homology proteins lack any experimental annotations in GOA.

For example, one protein, NUD12_MOUSE, has been assigned with two activities in GOA—NAD^+^ (GO:0000210) and NADH pyrophosphatase (GO:0035529) activities—for which the protein was not experimentally assayed (ironically, GOA cites this publication for exactly these two erroneous annotations).^54^

We found five proteins annotated as hydrolyzing the wrong substrate. For example, Yang *et al*. (2000)^138^ showed that NUD15_MOUSE hydrolyzes ADP‐ribose (GO:0080041), but GOA cites their work for nucleoside‐diphosphatase activity (GO:0017110).

Sixteen proteins were annotated as hydrolyzing the correct substrate but producing the wrong products. For example, Thorne *et al*. (1995)^51^ claim that AP4A_HUMAN cleaves Ap_4_A only asymmetrically (GO:0004081), but GOA cites their work and assigned Ap_4_A (symmetrical) pyrophosphatase activity (GO:0008803; TAS—traceable author statement).

Fourteen proteins were imprecisely annotated for a general function that does not reflect the specificities determined in the cited publications. For example, MUTY_ECOLI is annotated solely for hydrolase activity (GO:0016787) based on the work from Au *et al*. (1989).^41^ However, the same publication specifies that MUTY_ECOLI has 8‐oxo‐7,8‐dihydroguanine DNA N‐glycosylase activity (GO:0034039).

Seventeen proteins were annotated with functions that we believe are not sufficiently shown to be physiologically relevant in the current experimental literature because these functions have confidence scores below 0.2. For example, GOA cites the work of Fujikawa *et al*. (2001)^157^ to assign 8ODP_HUMAN with ATP diphosphatase activity (GO:0047693). In fact, the enzyme shows negligible activity on ATP (supporting information Fig. S2A in Fujikawa *et al*. (2001)),^157^ so we assigned a confidence score of 0.01 to this activity.

Finally, for 106 Nudix homology proteins that have experimental characterization data, GOA did not assign any experimental annotations. With the proposed GO terms (previous section), we assigned 837 protein‐function annotations, 275 of which have confidence scores above 0.2. As a result, 146 out of 171 Nudix homology proteins have at least one annotation with confidence scores above 0.2 in our collection.

### Structure alignment

**Figure 3:**
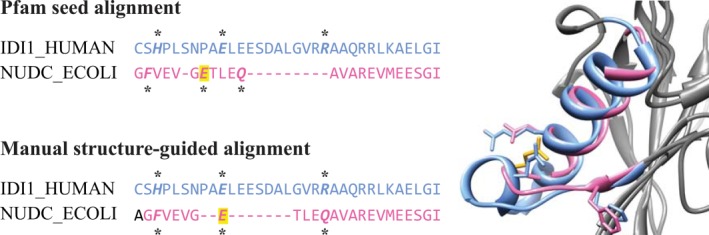
Misalignment of seed sequences in the Pfam database. Pairwise sequence alignment of human isopentenyl diphosphate isomerase 1 (IDI1—UniProt Entry Name: IDI1_HUMAN)^95^ and *E. coli* NADH pyrophosphatase (NudC—UniProt Entry Name: NUDC_ECOLI; JCSG PDB ID: 1VK6) extracted from the Pfam seed alignment for the Nudix family (v27.0—Pfam ID: PF00293) and from our manual structure‐guided sequence alignment. The Nudix boxes are colored in blue (IDI1) or pink (NudC). Residues denoted by asterisks are misaligned in the Pfam seed sequence alignment, but are positioned accurately in the manual structure‐guided alignment according to the side‐chain superimposition of these residues. The conserved glutamate of NudC is colored in yellow in both sequence alignments. The structural alignment was visualized with Chimera v1.6.2.^69^

Exploration of the evolution of function within the Nudix homology clan requires robust molecular phylogenetic analysis, which in turn generally depends upon accurate sequence alignment. Conventional methods of aligning Nudix homology protein sequences are imprecise because of the large degree of sequence divergence across the clan. One example is the manually curated Pfam seed alignment for the Nudix family (v27.0—Pfam ID: PF000293),^1^ where an insertion in human isopentenyl diphosphate isomerase 1 (UniProt Entry Name: IDI1_HUMAN) within the Nudix box misaligns a conserved catalytic glutamate residue with that of *E. coli* NADH pyrophosphatase (UniProt Entry Name: NUDC_ECOLI) (Fig. 3). This glutamate is notably conserved (boldface) in the Nudix box (GX_5_ **E**X_7_REUXEEXGU, where U is a hydrophobic residue and X is any amino acid)^12^ and potentially stabilizes the motif's loop‐helix‐loop structure, allowing for proper cation binding.^13^ This misalignment is potentially a result of the noncanonical spacing between conserved residues within IDI1_HUMAN's analogous Nudix box (SX_7_ **E**X_14_RRUXAEXGU). Such misalignments are easily detected and corrected when guiding the sequence alignment with an accurate structural alignment (Fig. 3). Structure‐templated alignments can lead to far more reliable results.^57^ For this reason, we developed a sequence alignment through a structural alignment of all determined Nudix structures.

Structural data for 78 Nudix homology proteins were gathered from the Protein Data Bank (PDB).^92^ Whenever there were multiple structures of the same protein, protein structures determined with bound substrate or product molecules were preferentially selected, as were those determined at higher resolutions (Table 5; also see Materials and Methods). The selected structures comprise a fairly diverse set of enzymes, including hydrolases for 17 different substrates, RNA decapping enzymes, isopentenyl diphosphate isomerases, A/G‐specific adenine glycosylases, and a transcription repressor. There are 34 structures for which there are no experimentally determined activities. *Escherichia coli* and human protein structures are highly represented (13 and 14 structures, respectively) in the dataset. There are two solved archaeal structures, but no viral structures. The final alignment of these 78 Nudix homology proteins, denoted as the 78‐PDB sequence alignment, revealed a number of notable features. We found that the overall length and the identity of functionally and structurally important amino acids within the Nudix box (GX_5_EX_7_REUXEEXGU) are conserved in 40 Nudix pyrophosphohydrolases. The other 38 Nudix homology domains, as considered by evolutionary methods, including those from A/G‐specific adenine glycosylases, isopentenyl diphosphate isomerases, and some hydrolases, lack the canonical Nudix box and show substitutions, deletions, and insertions within this region (supporting information Fig. S3).

The *Bacillus stearothermophilus* (PDB ID: 1RRS)^93^ and human (PDB ID: 1X51)^94^ A/G‐specific adenine glycosylases contain the Nudix box sequences CX_12_QMX_2_EQXGU and VX_2_EX_11_QEX_2_RWXG, respectively, while isopentenyl diphosphate isomerase (IDI) 1 (PDB ID: 2ICK)^95^ and IDI2 (PDB ID: 2PNY)^96^ from human and the *E. coli* IDI (PDB ID: 1NFS)^97^ exhibit GX_7_EX_14_RRX_2_AEXGU and GX_5_E_7_RRX_2_YEXGU, respectively. More intriguing are instances in which conserved residues within the Nudix hydrolase motif are absent or altered in other Nudix hydrolases: the *E. coli* GDP‐mannose diphosphatase *yffh* (PDB ID: 1VIU; Nudix box sequence: GX_4_DX_7_KEX_2_EEXGU)^98^ and the *Mycobacterium tuberculosis* ADP‐ribose diphosphatase *Rv1700* (PDB ID: 1MQW; Nudix box sequence: GX_6_EX_7_REX_2_EEXGU)^99^ show a shortening and lengthening, respectively, of the hydrolase motif, as well as specific substitutions in the case of *E. coli yffh*. The *E. coli* GDP‐sugar glycosyl hydrolases *nudD* (PDB ID: 1RYA)^38^ and *gmm* (PDB ID: 2I8T)^100^ both contain Nudix boxes that substitute two hydrophobic residues for two conserved glutamates (Nudix box sequence: GX_5_EX_7_RLX_2_AEXGU). Two snoRNA decapping enzymes (*nudt16* from *Xenopus laevis*—PDB ID: 2A8T;^101^ and *NUDT16* from human —PDB ID: 3COU^102^) exhibit a slight elongation of the motif and substitution of an aspartate for a conserved glutamate: GX_5_DX_8_REX_2_EEXGX. The pyrimidine nucleoside triphosphate diphosphatase DR_0079 from *Deinococcus radiodurans* (PDB ID: 2O5F)^49^ contains a substitution: in place of the final glycine there is an asparagine (Nudix box sequence: GX_5_EX_7_REX_2_EEXNU). Finally, the human cleavage and polyadenylation specificity factor NUDT21 (PDB ID: 2J8Q)^103^ contains multiple substitutions within the motif (Nudix box sequence: GX_5_EX_7_RLX_2_EIXGR).

### Proposed specificity determinants

**Figure 4:**
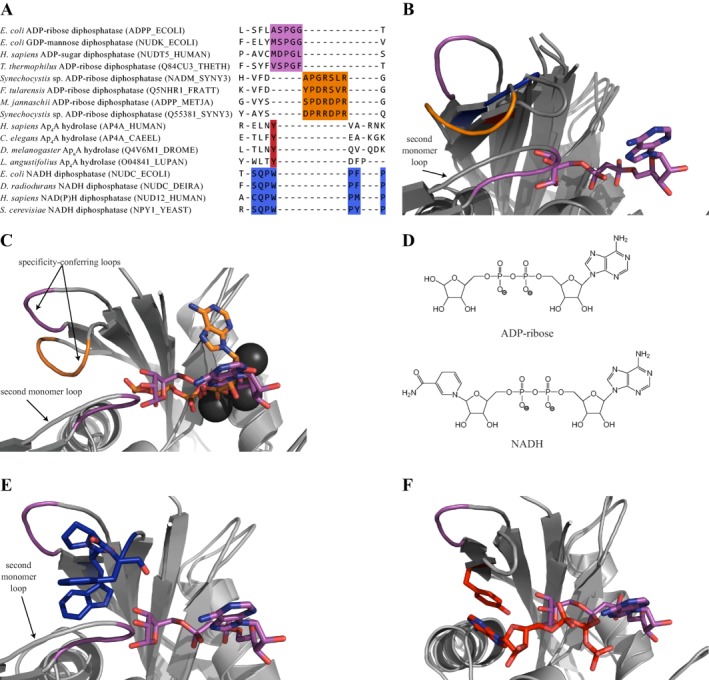
Specificity motifs reside in a loop (the X‐loop) that contacts the X‐moiety. (**A**) UniProt Entry Names of selected enzymes from the structure‐guided sequence alignment that exhibit specificity motifs in analogous loop regions downstream from the Nudix box motif. Motifs, either described by other investigators or presented in this study, are colored according to function, where purple and orange refer to two distinct types of NDP‐sugar diphosphatases, red refers to bis(5′‐nucleosyl) polyphosphate hydrolase activity, and blue refers to NADH diphosphatase activity. UniProt Entry Names follow each enzyme in parentheses and four enzymes are annotated with a PDB ID corresponding to the structure aligned in panel **B**. Enzymes under consideration include *E. coli* ADP‐ribose diphosphatase, *E. coli* GDP‐mannose diphosphatase, *Homo sapiens* ADP‐sugar diphosphatase, *Thermus thermophilus* ADP‐ribose diphosphatase, *Synechocystis* spp. ADP‐ribose diphosphatase, *Francisella tularensis* subsp. *tularensis* ADP‐ribose diphosphatase, *Methanococcus jannaschii* ADP‐ribose diphosphatase, *Synechocystis* spp. ADP‐ribose diphosphatase, *H. sapiens* Ap_4_A hydrolase, *Caenorhabditis elegans* Ap_4_A hydrolase, *Drosophila melanogaster* bis(5′‐adenosyl) polyphosphate (Ap_n_A) hydrolase, *Lupinus angustifolius* Ap_4_A hydrolase, *E. coli* NADH diphosphatase, *Deinococcus radiodurans* NADH diphosphatase, *H. sapiens* NAD(P)H diphosphatase, and *Saccharomyces cerevisiae* NADH diphosphatase. B. MultiProt^73^ multiple structural alignment of *Escherichia coli* ADP‐ribose diphosphatase^105^, *Synchocystis* spp. ADP‐ribose diphosphatase,^108^ *E. coli* NADH diphosphatase (JCSG PDB ID: 2GB5), and *Homo sapiens* Ap_4_A hydrolase.^115^ Specificity motifs associated with each enzyme's function are colored according to panel A. These motifs all reside within the same structural region that is in close proximity to the X‐moiety of the Nudix hydrolase substrate. The co‐crystallized substrate (ADP‐ribose) of *E. coli* ADP‐ribose diphosphatase is shown in purple to provide orientation. Two analogous loops, one from each *E. coli* ADP‐ribose diphosphatase monomer in the functional dimer, participate in contacting the terminal ribose moiety in the ADP‐ribose substrate. (**C**) DaliLite^72^ pairwise structural alignment of *E. coli* (PDB ID: 1KHZ) *Synechocystis* spp. (PDB ID: 2QJO) ADP‐ribose diphosphatases reveals general conservation with regard to the Nudix homology domain, but specific conformations with respect to a loop that contacts the substrate's ribose moiety (the “X‐loop”). The bound substrate analogue, α/β‐methylene‐ADP‐ribose (purple; with *E. coli* ADP‐ribose diphosphatase), three magnesium cations (black; with *E. coli* ADP‐ribose diphosphatase), and ADP‐ribose (orange; with *Synechocystis* spp. ADP‐ribose diphosphatase) are present. Highlighted in either purple or orange is a specificity and structural motif unique to *E. coli* ADP‐ribose diphosphatase or *Synechocystis* spp. ADP‐ribose diphosphatase, respectively. This region interacts with the X‐moiety of the substrate molecule (in this case, the terminal ribose component of ADP‐ribose). This region is referred to as the X‐loop. Two X‐loops, one from each *E. coli* ADP‐ribose diphosphatase monomer in the functional dimer, contact the terminal ribose moiety in the ADP‐ribose substrate. (**D**) Structures of ADP‐ribose and NADH are identical except at the terminal ribose group, where NADH bears a reduced nicotinamide moiety that potentially contacts conserved residues present in the NADH diphosphatase specificity motif. **E.** DaliLite^72^ pairwise structural alignment of *Escherichia coli* NADH diphosphatase (PDB ID: 2GB5) (blue) and *E. coli* ADP‐ribose diphosphatase (PDB ID: 1KHZ) (purple). A portion of the NADH diphosphatase specificity motif, which most likely contacts the nicotinamide moiety, is in the stick representation. The cocrystallized substrate (ADP‐ribose) of *E. coli* ADP‐ribose diphosphatase is shown in purple. Specific residues within the NADH diphosphatase specificity motif are positioned so as to interact with the nicotinamide moiety missing from the terminal ribose portion of ADP‐ribose. (**F**) DaliLite pairwise structural alignment of *E. coli* ADP‐ribose diphosphatase (purple; PDB ID: 1KHZ) and *Homo sapiens* Ap_4_A hydrolase (red; PDB ID: 1XSC) demonstrating that the bis(5′nucleosyl) polyphosphate hydrolase specificity motif (one conserved tyrosine) interacts with the adenine ring of the ATP product (red; bound to *H. sapiens* Ap_4_A hydrolase). The cocrystallized substrate (ADP‐ribose) of *E. coli* ADP‐ribose diphosphatase is shown in purple to demonstrate that the terminal ribose X‐moiety of ADP‐ribose and the ATP moiety of Ap_4_A occupy similar environments and contact the same X‐loop region of their respective catalysts. Thus, the X‐moiety for human Ap_4_A hydrolase would be ADP, which interacts with the specificity motif (conserved tyrosine). The sequence alignment was visualized with Jalview v2.8,^70^ all structural alignments were visualized with PyMOL v0.99,^67^ and graphics processed with Adobe Illustrator CS4.^114^

Given that the Nudix homology domain is an effective structural scaffold for many catalytic activities, it is of particular interest to understand the evolution of substrate specificity in the clan. Within all solved Nudix structures to date, the Nudix box adopts a loop‐helix‐loop motif, but in the case of Nudix hydrolases this feature only recognizes the pyrophosphate moiety common to all substrates. Currently, there is a limited understanding of how other structural elements may generate substrate specificity.^13^, ^23^, ^36^, ^104^ Dunn *et al*. (1999)^52^ identified conserved protein sequence motifs that are unique to some ADP‐ribose diphosphatases, NADH diphosphatases, and Ap_n_A hydrolases. These motifs are all downstream of the Nudix motif and include the amino acid sequence SQPWPFPQS [blue box, Fig. 4(a)] that correlates with NADH diphosphatase activity; a proline residue [within pink box, Fig. 4(a)] common to ADP‐ribose diphosphatases; and a tyrosine residue [red box, Fig. 4(a)] in a similar position as the above described motifs that coincides with activity on diadenosine polyphosphates.

Our structural alignment and analysis of Nudix homology proteins revealed that these functional motifs all localize to a specific structural region [Fig. 4(b)]. Specifically, this portion of the Nudix homology domain forms a loop (typically 5–10 amino acids, but can be as short as 2 and as long as 19 in the 78‐PDB sequence alignment) that is in the active site and makes specific contacts with the “X” moiety of Nudix hydrolase substrates [Fig. 4(b)]. This suggests that modifications within this region could alter substrate specificity, thus allowing for neofunctionalization. It seems likely that this loop selects for the identity of the “X” moiety, and we therefore designate it as the “X‐loop.”

The substrate in the *E. coli* ADP‐ribose diphosphatase dimer structure is oriented in the active site such that the terminal ribose points toward the X‐loop, containing the conserved proline residue [purple, Fig. 4(c)]. The X‐loop from each monomer participates in two separate active sites and contacts the substrate's ribose moiety within each.^105^ While a substrate‐bound NADH diphosphatase structure has not been solved, the structural similarity between ADP‐ribose and NADH [Fig. 4(d)] allows for an extrapolation of our understanding of the ADP‐ribose diphosphatase active site to the *E. coli* NADH diphosphatase. The previously identified NADH diphosphatase motif, SQPWPFPQS,^52^ resides in a loop analogous to that containing the conserved ADP‐ribose diphosphatase motif (single proline) in a structural alignment of *E. coli* ADP‐ribose diphosphatase with *E. coli* NADH diphosphatase [Fig. 4(e)]. Given that the sugar moiety of ADP‐ribose contacts this loop in *E. coli* ADP‐ribose diphosphatase, it is likely that NADH associates similarly with NADH diphosphatase such that its pyridine moiety would contact the NADH diphosphatase motif. Thus, ADP‐ribose and NADH diphosphatases would achieve specificity by distinguishing the chemical entities on the terminal ribose (the pyridine versus hydroxyl moieties). Structural alignment of *E. coli* ADP‐ribose diphosphatase with human Ap_4_A hydrolase further demonstrates analogous roles that these X‐loops play in recognizing the “X” moiety; the conserved tyrosine (residue 87 in human Ap_4_A hydrolase) in Ap_n_A hydrolases localizes to this region and plays a direct role in contacting the substrate [Fig. 4(f)].

Similarly located “X‐loop” regions in Nudix enzymes potentially or demonstrably contact the substrate in the same manner in all cases. The polyphosphate chain, which is common to all Nudix hydrolase substrates, is bound by the Nudix motif, which constrains the bound substrate to orient the “nucleoside” and “X” moieties in specific locations within the active site. As substrate specificity can be sharpened via interactions with either end of the polyphosphate linkage, it is reasonable to expect structural regions of the protein that contact those ends of the molecule to display sequence conservation. Furthermore, new substrate specificities may be achieved by modifying the amino acid sequence in these regions. For example, the specificities of some ADP‐ribose diphosphatases corroborate this model of functional radiation as they exhibit significant activity for substrates that possess a common ADP core, but have varying X moieties. *Escherichia coli* nudE Nudix hydrolase is active on ADP‐ribose, NADH (ADP plus a pyridine nucleoside moiety), Ap_2_A (ADP plus an adenine nucleoside moiety), and Ap_3_A (ADP plus an AMP moiety).

This postulated mode of substrate recognition could explain the occurrence of likely homoplasy in the proposed evolutionary history of the Nudix homology clan (see next section). Separate evolutionary lineages may converge on the same function as a result of similar selective pressures on the same active site region. Because multiple regions within the active site determine substrate specificity, the accumulation of amino acid substitutions in the X‐loop could broaden the specificity for one end of the Nudix substrate molecule, while sequence conservation at other locations would maintain specificity for the other substrate end. This postulated mutation pattern would thus constitute an evolutionary mechanism for gradual functional differentiation while preserving catalytic activity, as carried out by residues in the Nudix box.

### Phylogeny of select nudix homology proteins

We began the phylogenic analysis of the Nudix homology clan with a select set of 347 Nudix homology domains, the members of which match at least one of the following criteria: have solved structures, have experimentally assigned functions, or are included in the seed alignment of the Pfam Nudix family (v27.0; Pfam ID: PF00293). Overall, this collection of Nudix homology proteins covers broad organismal diversity. Members belong to all three domains of life as well as to 11 viral sources. Later we performed a phylogenetic analysis of the complete Nudix homology clan using an approach motivated by our analysis of this set of 347 Nudix homology domains (see next section).

Neither X‐ray nor NMR structures are available for most of the collected Nudix homology domains (269 out of 347); therefore, we attempted to guide the alignment of these sequences with the aforementioned 78‐PDB sequence alignment. We used HMMER^79^ and MAFFT^80^ to expand the alignment from 78 to 324 sequences, manually inspected the results from these methods, and curated the alignment. We denote this alignment as the 324‐core alignment. The remaining 23 (= 347 – 324) Nudix homology domains, all of which had been found to have experimental annotations after the 324‐core alignment was constructed, were aligned to the 324‐core alignment in the same way as the other domains in the Nudix homology clan (see the next section) (supporting information Table S1, Resource 19). This yielded the 347‐select alignment (supporting information Table S1, Resource 20). A phylogenetic tree was constructed from this structure‐guided sequence alignment using RAxML,^78^ with 100 random starting trees and 1000 bootstrapping interactions, and reconciled using Forester^85^ with a species tree from iTOL^84^ (see Materials and Methods).

**Figure 5:**
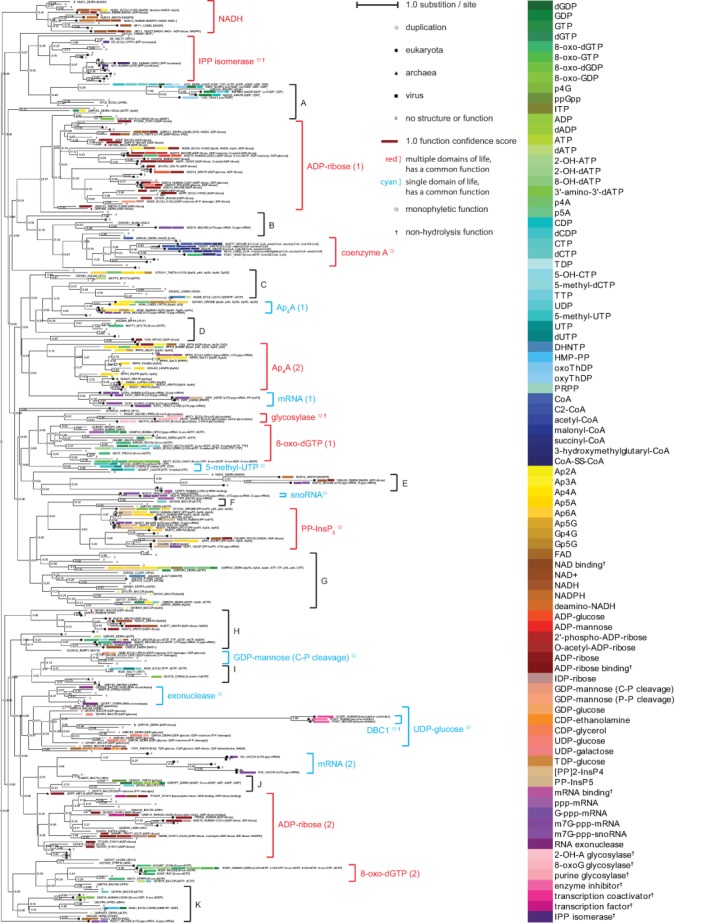
Phylogeny of 347 structurally determined, functionally characterized, or phylogenetically important Nudix homology protein domains. The phylogenetic tree, constructed with RaxML^78^ with 1,000 bootstraps, was midpoint rooted. The bootstrap value of an internal node is placed next to it. Inferred duplication events are represented by open diamonds on the corresponding internal nodes. Every leaf has domain of life, function, and ID notations. The domain of life of a leaf is indicated by a circle (eukaryota), a triangle (archaea), a square (virus), or is left blank (bacteria). The characterized functions, where known, are indicated with colored bars, whose lengths are proportional to the confidence scores of function assignments. The colors of the bars are chosen to indicate the biochemical similarity (e.g., substrate structure, position of hydrolysis) of the functions. Abbreviations for annotated functions (usually substrate names) are listed in a pair of square brackets following the color bars. The UniProt Entry Name of the protein and, if available, the associated PDB ID are noted in parentheses. If neither function nor structure is available for a leaf (as is the case for protein domains from the seed alignment of the Pfam Nudix family (PF00293)), its UniProt Entry Name is omitted, and a cross is used as a placeholder. Red and cyan brackets with shorthand notation of functions indicate clades within which most characterized proteins share at least one common function. Red brackets have proteins from at least two domains of life, while blue brackets mean that all proteins within the clades are from the same domain of life. Functionally monophyletic clades are noted by “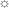,” and nonhydrolase functions are noted by “†,” both next to the names of the clades. Protein clades that separate these designated clades are indicated by black brackets with letters. The asterisks in the text of the color bar legend indicate nonhydrolase functions. A high‐resolution version is available in supporting information Table S1, Resource 26. The phylogeny was visualized with Dendroscope v3.2.8^83^ and graphics processed with Adobe Illustrator CS4.^114^

For convenience, we attempted to root the tree using midpoint rooting (with Dendroscope^83^). The majority (*ca*. 90%) of the Nudix homology domains belong to the Nudix pyrophosphatase Pfam family (NUDIX; Pfam ID: PF00293). Therefore, we also attempted to root the tree using outgroup rooting with either A/G‐specific adenine glycosylases (NUDIX_4; Pfam ID: PF14815) or DBC1 proteins (DBC1; Pfam ID: PF14443), as both belong to different Pfam families but fall under the same Nudix clan (Pfam ID: CL0261) as the Nudix pyrophosphatase Pfam family. However, we found that midpoint rooting^106^ generated the same or fewer number of duplication events compared to the outgroup rooting. Therefore, for convenience, midpoint rooting was chosen for reconciliation and subsequent analysis. The overall distribution of bootstrap values of clades across the tree provides moderate support for most clades; more “ancient” nodes typically have less support, likely reflecting the large degree of sequence divergence within the Nudix homology clan that is difficult to resolve even with manual structural and sequence alignment. The function assignments of Nudix homology proteins were annotated using color bars and were mapped to the above tree, with confidence scores of the assignments (mentioned briefly in Data Sources and Analysis above; see Materials and Methods for details) proportional to the lengths of the bars (Fig. 5).

The resulting phylogeny of the select Nudix homology domains allowed us to generate hypotheses and make some inferences regarding functions in ancestral Nudix homology proteins, which are resilient to the challenges of tree rooting. We argue that multiple ancestral Nudix homology proteins with at least seven different functions (see the paragraphs entitled “Ancient functions” below) existed in the last universal common ancestor of life (LUCA). We found that these seven functions are in clades whose characterized proteins all share a common function. Each clade contains proteins from more than one domain of life. These functions likely existed in the LUCA, and have been retained in subsequent speciation into different domains of life and beyond. We also identified eight functions (see the paragraphs entitled “Monophyletic functions” below) that are likely to have evolved later, as these functions are only present within one clade that contains proteins from only one domain of life. It is still possible that some of these eight functions existed in the LUCA, but have not been discovered in other domains of life so far, or such functions might have been lost in other domains of life during evolution. Finally, we noticed that many functions are shared in multiple distinct small clades widespread in the tree, which often suggests homoplasy or frequent gene loss (see the paragraphs entitled “Polyphyletic functions” below). However, some of these widespread functions are commonly tested in the literature and are often assigned activities despite weak evidence, thus having low confidence scores. Therefore, some of those functions may not actually be physiologically relevant. Several are annotated in clades with long branch‐lengths and low bootstrapping support, and thus could be due to tree reconciliation artifacts and errors.

#### Ancient functions

Ten clades whose characterized proteins all share a common function contain proteins from more than one domain of life (Fig. 5, red brackets, viral proteins ignored): NADH pyrophosphatase, IPP isomerase, ADP‐ribose pyrophosphatase (in two clades), coenzyme‐A pyrophosphatase, Ap_4_A hydrolase, A/G‐specific adenine glycosylase, 8‐oxo‐dGTP pyrophosphatase (in two clades), and PP‐InsP_5_ pyrophosphatase. It is likely that except for PP‐InsP_5_ (see below), the other seven functions existed in LUCA, and have been retained in subsequent speciation into different domains of life and beyond (for a review of functional versatility of proteins in LUCA, see Ranea *et al*. (2006)).^107^ However, it is also possible that some leaves were misplaced during the phylogeny construction process.

The distinct and separate phylogenetic clustering of these protein clades, and the fact that they all contain members from at least two domains of life, suggests their associated functions were present in the LUCA. This implies that the LUCA had multiple paralogs of Nudix homology proteins with varied function. For example, there are two major clades of ADP‐ribose pyrophosphatases, each of which contains proteins from all three domains of life. Structural comparison of proteins from these two clades (ADPP_ECOLI in ADP‐ribose (1) and NADM_SYNY3 in ADP‐ribose (2))^108^ demonstrated different modes of dimerization and domain swapping among the enzymes. The structural alignment between these two proteins [Fig. 4(c)] shows that while the major secondary structural elements of each are similar, the conformation of a loop region situated downstream of the Nudix box differs significantly. This conformational divergence affects the binding of the terminal ribose of the ADP‐ribose substrate, and may be considered a factor that differentiates these two groups of ADP‐ribose diphosphatases. Outside these two clades, several other ADP‐ribose pyrophosphatases (within brackets NADH, Ap_4_A (2), PP‐InsP_5_, E and H) are also found widely spread in the tree.

Of these 10 aforementioned multiple‐domains‐of‐life clades (Fig. 5, red brackets, viral proteins ignored), two have just one leaf from a domain of life different from the other members of the clades (8‐oxo‐dGTP (1): triangle above Q9RXN6_DEIRA; PP‐InsP_5_: cross below DDP1_YEAST). Neither of these two leaves was included in the structure‐guided sequence alignment, nor do they have any degree of experimental characterization. Additionally, both leaves have long branch‐lengths and low bootstrap support. These observations raise a possibility that these two leaves might be misaligned or misplaced in the phylogeny construction. Diphosphoinositol polyphosphates like PP‐InsP_5_ may contribute to regulating intracellular trafficking, and may participate in the regulation of mRNA export from the nucleus.^109^, ^110^ We are not aware of evidence supporting PP‐InsP_5_ utilization in bacteria or archaea. This consideration, in combination with the possible mispositioning of the PP‐InsP_5_ clades, lead us to believe that PP‐InsP_5_ pyrophosphatase activity likely would have arisen later in evolution.

#### Monophyletic functions (functions present only within a clade but nowhere else in the tree)

There are 10 major monophyletic clades of molecular function in the phylogeny (Fig. 5, brackets with “
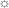
”): IPP isomerase, coenzyme‐A pyrophosphatase, A/G‐specific adenine glycosylase, 5‐methyl‐UTP pyrophosphatase, snoRNA decapping enzyme, PP‐InsP_5_ pyrophosphatase, GDP‐mannose mannosyl hydrolase (carbon‐phosphorus cleavage), RNA exonuclease, DBC1 protein family, and UDP‐glucose pyrophosphatase. Except for IPP isomerase, coenzyme‐A pyrophosphatase, and PP‐InsP_5_ pyrophosphatase, the other seven monophyletic clades contain members from only one domain of life. These seven functions and PP‐InsP_5_ pyrophosphatase activity (see above) are likely to have evolved later, following divergence of the major domains of life. However, it is possible that proteins from other domains of life have not yet been discovered to perform these function, or that such functions were present in other domains of life and have been lost during evolution. Therefore, we cannot exclude the possibility that these functions were present in the LUCA. Note that PP‐InsP_5_ pyrophosphatase activity is also shown as a secondary function for a viral protein (DIPP_ASFB7, within bracket mRNA (1)), which might suggest a horizontal gene transfer event between eukaryota and virus.

The DBC1 protein family, which belongs to the Nudix homology clan but a different Pfam family (Pfam ID: PF14443), is placed inside the UDP‐glucose/UDP‐galactose clade. This might be due to a misplacement of this clade in the phylogeny construction for several reasons. First, the branch length at the root of this clade is very long, indicating a clear separation of DBC1 from the rest of the UDP‐glucose/UDP‐galactose clade. Second, the branch length between the parent and grandparent of the DBC1 is zero, indicating an ambiguous separation. Third, the bootstrapping support for such placement is zero, implying that the placement of DBC1 is effectively not reproducible in the bootstrapping iterations. Finally, this placement was not reproduced in the phylogeny of the complete Nudix homology clan (next section), which also implies the unreliability of the placement.

#### Polyphyletic functions (functions present in multiple clades of the tree)

The most widely spread function throughout the tree is guanosine polyphosphate pyrophosphatase, which is found in eleven clades (Fig. 5, brackets A, ADP‐ribose (1), coenzyme A, D, 8‐oxo‐dGTP (1), PP‐InsP_5_, G, H, J, 8‐oxo‐dGTP (2), K). High confidence values (>0.8) were found in two single function clades (8‐oxo‐dGTP (1) and 8‐oxo‐dGTP (2)) as well as for five other proteins in the tree. The structural and sequence similarities are poor between *E. coli* 8‐oxo‐dGTP diphosphatase (within bracket 8‐oxo‐dGTP (1); UniProt Entry Name: MUTT_ECOLI; PDB ID: 1PPX^111^) and human 8‐oxo‐dGTP diphosphatase (within bracket 8‐oxo‐dGTP (2); UniProt Entry Name: 8ODP_HUMAN; PDB ID: 1IRY^112^). These data suggest that this function might have arisen multiple times independently in the course of evolution, or that ancestral genes carrying this function have been lost frequently. Also, some of the disparity may be due to frequent assays for 8‐oxo‐dGTP hydrolysis that are sensitive to nonphysiological levels of activity.

The second most widespread function throughout the tree is mRNA decapping activity, which is found in nine clades (brackets B, Ap_4_A (1), Ap4A (2), mRNA (1), 8‐oxo‐dGTP (1), snoRNA, PP‐InsP_5_, F, H, mRNA (2), K). Three of these clades have high confidence scores (> 0.8) for the mRNA function (F, mRNA (1), mRNA (2)), while the others have moderate scores (∼0.5). All high confidence scores of mRNA decapping activity were assigned to eukaryotic or viral proteins. The only bacterial protein capable of decapping mRNA *in vitro*, RPPH_ECOLI, has a moderate confidence score for such activity, as the activity was characterized only by electrophoresis imaging in one paper.^89^ We therefore believe that mRNA decapping activity among Nudix homology proteins evolved after eukaryotes and bacteria separated, and that the mRNA decapping activity of RPPH_ECOLI demonstrates the functional versatility of this enzyme.

The presence of mRNA decapping enzymes (bracket mRNA (1)) adjacent to the Ap_n_A clade (bracket Ap_4_A (2)) suggests a shared evolutionary origin and hints at a common substrate‐recognition mechanism for long polyphosphate chains that bridge nucleosides. mRNA decapping enzymes cleave 7‐methyl‐GDP from capped mRNA transcripts, allowing for the eventual degradation of mRNA. It is notable that the general mRNA cap structure resembles that of a dinucleoside polyphosphate, especially with respect to the 5′‐5′‐polyphosphate linkage between two nucleosides. Biochemical evidence from *Lupinus angustifolius* Ap_4_A hydrolase (UniProt Entry Name: O04841_LUPAN) corroborates the notion that an Ap_n_A hydrolase may be able to recognize an mRNA cap structure due to the structural similarities of the substrates. This enzyme catalyzes the hydrolysis of dinucleoside polyphosphate mRNA cap analogues, in particular 7‐methylguanosine‐5′‐triphosphonucleosides.^113^

Further evidence for a shared evolutionary origin for Ap_n_A and general RNA activities can be found in a publication demonstrating RNA polyphosphohydrolase activity (removal of pyrophosphate from the 5′‐triphosphate end of RNA), distinct from mRNA decapping activity (hydrolysis of the m^7^G cap from m^7^G‐ppp‐RNA), for a previously described diadenosine polyphosphate hydrolase (*E. coli* Ap_5_A hydrolase *rppH*)^90^. Given the relationship between *rppH* and other characterized diadenosine polyphosphate hydrolases in our dataset (Fig. 5, bracket Ap_4_A (2)), it may be possible that Ap_n_A hydrolases present in the tree are capable of RNA polyphosphohydrolase activity.

#### Loss of conserved nudix box residues

The GDP‐mannose mannosyl hydrolases in our dataset (Fig. 5, within bracket GDP‐mannose (carbon‐phosphorus cleavage)) are separated from most other NDP‐sugar hydrolases (e.g., brackets ADP‐ribose (1)/(2), UDP‐glucose). This may reflect the experimentally verified distinct catalytic mechanisms employed by GDP‐sugar glycosyl hydrolases and NDP‐sugar diphosphatases: GDP‐sugar glycosyl hydrolases catalyze the formation of GDP and sugar products through nucleophilic attack at the nucleoside 5′‐carbon atom, whereas all other investigated NDP‐sugar diphosphatases yield NMP and phosphorylated sugar products through nucleophilic substitution at a phosphorus atom within the diphosphate moiety.^38^ The loss of key Nudix motif residues among the GDP‐sugar glycosyl hydrolases also highlights their mechanistic divergence from other Nudix enzymes.^38^ More broadly, the separate clustering of GDP‐sugar glycosyl hydrolases, A/G‐specific adenine glycosylases, isopentenyl diphosphate isomerases, and the cleavage and polyadenylation specificity factor (UniProt Entry Name: CPSF5_HUMAN; within bracket F), all proteins that display changes in conserved residues in the Nudix motif, indicates that they do not share a recent common ancestor and that the loss of conserved Nudix box residues occurred more than once.

#### Challenges in nudix phylogenetic function analysis

The limited extent of experimental characterization of Nudix homology clan members has a significant impact upon interpreting functions in the context of phylogeny. One example is NUDB_ECOLI (Fig. 5, within bracket C), an enzyme initially characterized as a dATP diphosphatase, but was later found to have a much higher activity on dihydroneopterin triphosphate (DHNTP).^63^ This protein in the current analysis clusters with Ap_n_A hydrolases (bracket Ap_4_A (1)). NUDB_ECOLI was not assayed on Ap_n_As, so its placement in this clade may reflect a functional divergence, or it may represent a continuing incomplete characterization (that is, undiscovered Ap_n_A hydrolase activity) of this protein. There is another protein currently annotated for DHNTP diphosphatase activity (Q4U4W6_9LACT; within bracket G), so our phylogenetic analysis suggests that DHNTP diphosphatase activity is either ancestral or homoplastic. If more Nudix homology proteins were assayed with DHNTP, it would have been clearer if DHNTP diphosphatase activity is ancestral or not.

#### Phylogeny of the complete nudix homology clan

Pfam v27.0 defines the Nudix clan (CL0261) as containing five member families: NUDIX (PF00293), DBC1 (PF14443), NUDIX‐like (PF09296), NUDIX_2 (PF14815), and NUDIX_4 (PF13869). We retrieved all protein domains in UniProt release 2013‐04 that match any of these five families, resulting in a collection of 80,616 domain sequences. By removing identical sequences and 119 proteins without clearly defined species, we identified 38,950 unique sequences. We used the structure‐guided sequence alignment mentioned above as a seed for MAFFT^80^ to align these 38,950 sequences. We then used FastTree^81^ to construct the phylogeny of these sequences with midpoint tree rooting (see Materials and Methods for detail).

The resulting tree (supporting information Fig. S4) preserves most of the monophyly and polyphyly present in the phylogeny of the selected Nudix homology proteins (Fig. 5). For example, within this larger phylogeny, ADP‐ribose diphosphatases predominantly segregate into two large distinct clades, each with eukaryotic and prokaryotic members, further corroborating the results of the phylogeny of assayed proteins and the hypothesis that the LUCA contained more than one ADP‐ribose diphosphatase. CoA diphosphatases remain clustered in one clade in the superfamily phylogeny in agreement with the select Nudix phylogeny. The polyphyletic distribution of NTP diphosphatases in the phylogeny of the 347‐select Nudix homology domains is also observed in the phylogeny of the Nudix homology clan.

The major change when comparing the two phylogenies is the relative positioning among clades. For example, DBC1 now is positioned as a clade distinct from UDP‐glucose, which is consistent with our hypothesis that it is mis‐positioned in the phylogeny of the select Nudix homology proteins. A minor change is the distribution of domains of life in two clades: DBC1 and UDP‐glucose. In the phylogeny of the whole Nudix homology clan, the DBC1 clade has one bacterial sequence within all the 141 eukaryotic ones. The branch length of this sequence, however, is abnormally long (data not shown), indicating that this might be an error introduced in the phylogeny construction process. The UDP‐glucose clade contains 574 leaves, all from bacteria except one from a virus. The branch length for the viral sequence is similar to that of its neighbors (data not shown.), so it is unclear whether this is also an error from the phylogeny construction process. Despite these two outliers, the other clades are very similar in function and domain of life distributions. The conclusion that the common ancestor of the Nudix homology clan is likely to be functionally promiscuous remains supported.

## CONCLUSIONS

We have manually curated the literature and documented 171 experimental and 78 structural characterizations for a total of 205 Nudix homology proteins. Our subsequent manual structure‐guided sequence alignment of these 205 proteins plus 135 seed proteins from the Pfam Nudix family led to a phylogenetic reconstruction of the Nudix homology clan that demonstrates homoplasy for some functions, but general monophyly for most. Further analysis of the evolution of Nudix function revealed that the last universal common ancestor to all life most likely possessed NADH pyrophosphatase, IPP isomerase, ADP‐ribose pyrophosphatase, coenzyme‐A pyrophosphatase, Ap_4_A hydrolase, A/G‐specific adenine glycosylase, 8‐oxo‐dGTP pyrophosphatase, and PP‐InsP_5_ pyrophosphatase activities. Our literature search stimulated a reevaluation of the Gene Ontology hierarchy with respect to hydrolase activities relevant to Nudix hydrolases, resulting in the finding that Gene Ontology Annotation classifications of Nudix gene products are broadly lacking in specificity and in some cases accuracy. Finally, we identified a loop region (termed the “X‐loop”) approximately 17 amino acids downstream of the Nudix motif in hydrolases that plays a role in providing substrate specificity in the active site, and provides a basis through which functional diversification may evolve. Due to its proximity to the “X” moiety of a Nudix hydrolase substrate, amino acid substitutions within the X loop would directly affect the enzyme's specificity for the “X” moiety and thus the substrate. This suggests that protein neofunctionalization may readily be achieved through a small number of sequence changes localized to this area of the protein.

## Supporting information

Table S1. Electronic resources related to the structural, sequence, and functional analysis of the Nudix superfamily (DOI:10.6078/D1CC74).
Figure S1. Pipelines to build sequence alignments of the Nudix superfamily. The detailed steps are described in the Materials and Methods section. (**A**) The pipeline to build the 78‐PDB structure‐guided sequence alignment. (**B**) The pipeline to build the 324‐core sequence alignment guided by the 78‐PDB sequence alignment. (C) The pipeline to build the alignment of the complete Nudix clan (38,950 sequences). (**D**) Illustration of how to combine two alignment into one guided by a scaffold alignment.
Figure S2. Proposed Gene Ontology hierarchy. This region of the classification is a strict hierarchy, not a DAG, except as indicated below. New terms are begun with either A (terms with experimental support) or AP (parent terms proposed only for the structure of the hierarchy). The relationships between parent and child terms are represented with indents. The proposed changes, when applicable, are indicated by superscripts in front of GO term IDs: a—change name; b—change definition; c—change parent; d—add parent; e—remove; f—newly proposed. When a term has two parents, the term is shown in black under one parent and gray in the other. The removed term is indicated with strikethrough.
Figure S3. Nudix box sequences in the structure‐induced sequence alignment. UniProt Entry Names and PDB IDs of 78 Nudix enzymes and their Nudix box sequences are shown in the alignment. The most conserved positions are indicated in bold on top of the alignment. The top 38 enzymes have at least one substitution in one of the most conserved positions, or one insertion or deletion between the conserved positions. The Clustal X color scheme was applied to all residues.
Figure S4. Phylogeny of the complete Nudix clan, with 38,950 unique Nudix domains that match the HMM of one of the Pfam families in Pfam v27.0 (Mar 2013). The phylogeny was constructed with FastTree 64 and midpoint rooted. The branch lengths of the tree are omitted for simplicity. The protein domains with experimental annotations are the same as those in Fig. 5. The domains of life of leaves are indicated on the side of the phylogeny as follows: solid diamonds (bacteria), circles (eukaryota), triangles (archaea), or squares (virus). The clades, functions, and IDs of leaves are indicated as in Fig. 5 for protein domains with experimental annotations. When the text labels of multiple leaves overlapped, one leaf is shown in black, and others are shown in light gray. A high‐resolution version is available in Table S1, Resource 2.
