# Supplementary material for: The evolution of function within the Nudix homology clan
Source: Proteins. 2017 Mar 16;85(5):775–811. doi: 10.1002/prot.25223 (PMC5389931; doi:10.1002/prot.25223)
Supplement: Supplementary file 1 — Table S1. Electronic resources related to the structural, sequence, and functional analysis of the Nudix superfamily (DOI:10.6078/D1CC74). Figure S1. Pipelines to build sequence alignments of the Nudix superfamily. The detailed steps are described in the Materials and Methods section. (A) The pipeline to build the 78‐PDB structure‐guided sequence alignment. (B) The pipeline to build the 324‐core sequence alignment guided by the 78‐PDB sequence alignment. (C) The pipeline to build the alignment of the complete Nudix clan (38,950 sequences). (D) Illustration of how to combine two alignment into one guided by a scaffold alignment. Figure S2. Proposed Gene Ontology hierarchy. This region of the classification is a strict hierarchy, not a DAG, except as indicated below. New terms are begun with either A (terms with experimental support) or AP (parent terms proposed only for the structure of the hierarchy). The relationships between parent and child terms are represented with indents. The proposed changes, when applicable, are indicated by superscripts in front of GO term IDs: a—change name; b—change definition; c—change parent; d—add parent; e—remove; f—newly proposed. When a term has two parents, the term is shown in black under one parent and gray in the other. The removed term is indicated with strikethrough. Figure S3. Nudix box sequences in the structure‐induced sequence alignment. UniProt Entry Names and PDB IDs of 78 Nudix enzymes and their Nudix box sequences are shown in the alignment. The most conserved positions are indicated in bold on top of the alignment. The top 38 enzymes have at least one substitution in one of the most conserved positions, or one insertion or deletion between the conserved positions. The Clustal X color scheme was applied to all residues. Figure S4. Phylogeny of the complete Nudix clan, with 38,950 unique Nudix domains that match the HMM of one of the Pfam families in Pfam v27.0 (Mar 2013). The phylogeny was constructed with FastTree 64 [file PROT-85-775-s001.pdf]

Table S1. Electronic resources related to the structural, sequence, and functional analysis of the Nudix superfamily (DOI:10.6078/D1CC74).

Resource Number	Title	Type	Explanation	Digital Object Identifier (DOI)
1	mysql_dump_char+nudix_04062014.sql	SQL	MySQL database dump file. The database contains experimental data on Nudix proteins with journal references. Refer to Resource 2 for detailed information of its structure.	10.6078/d17p4v
2	The layout of the Nudix MySQL database.pdf	PDF	Schematic schema of MySQL database containing Nudix protein names, relevant experimental data and their source references. Each box indicates a MySQL table. The arrows represent the foreign key linkages between tables. The data entries are stored as kinetic, screening or genetic data, each with distinct table organizations. Each data is linked with a GO term. The references are stored as either PubMed IDs or DOI addresses.	10.6078/d13w2x
3	Nudix data collection.xlsx	Excel	Summary of Nudix enzyme's functions collected from the literature. All experimental data for Nudix proteins were categorized into six different evidence types (represented by six sheets of this file): 1) kcat/Km, 2) kcat, 3) Km, 4) relative activity, 5) other biochemical activity (*e.g.* gel electrophoresis, HPLC, X-ray crystal structure), and 6) genetic evidence (*e.g.* knockouts, complementation). All data are compiled in a master sheet "all data" where each row corresponds to one protein activity pair. Additional rows are included where a given activity was investigated more than once. This file also has a sheet "GO terms" for all the Gene Ontology terms associated with at least one activity.	10.6078/d1059d
4	Initial score assignment.xlsx	Excel	To describe confidence in function assignment of Nudix proteins, six evidence types were first classified as either biochemical or genetic, and biochemical evidence were further categorized into four subcategories: 1) kcat/Km, 2) pseudo kcat/Km (explained below), 3) relative activity, and 4) others (represented by five sheets of this file). All data points were assigned a score based on our scoring scheme (Table 4). In each sheets, the data and their corresponding scores are shown side-by-side. There is an additional sheet "kk used for pseudo kk", which contains a set of protein activities that had both their kcat/Km values and relative activities determined. In such cases, the kcat/Km value was used to approximate pseudo kcat/Km values for other relative activities in the same screening group.	10.6078/d1vc7g
5	Final score assignment.xlsx	Excel	To describe confidence in function assignment of Nudix proteins, the two sheets "biochemical scores" and "genetic scores" combine all initial score assignments that belong to the two categories. Within the category, the maximum score of a given protein function was designated as $S_{biochem}$ and $S_{genetic}$ in the sheet "overall scores". The overall score was calculated by the equation: $S_{overall} = 1 - (1 - S_{genetic}) \times (1 - S_{biochem})$. This score was adjusted to	10.6078/d1qp46

			take into account the abundance of annotations for a given enzyme using an R script (explained below). The sheet "final scores" shows the score before ($S_{overall}$) and after the adjustment (S_{final}).	
6	generate_score_biochem_genetic.R	R script	This script combines biochemical and genetic confidence scores to yield and integrated score. Two sheets, "score_biochem.csv" (Resource 7) and "score_genetic.csv" (Resource 8) are used as the input files of the R script "generate_score_biochem_genetic.R", which yields an output file, "score_biochem_genetic.csv" (Resource 9). $S_{overall}$ is calculated as an additional column in the sheet, and the result is saved as "score_biochem_genetic_soverall.csv" (Resource 10).	10.6078/d1kw28
7	score_biochem.csv	csv	Input file for Resource 6; derived from Resource 5.	10.6078/d1g59r
8	score_genetic.csv	csv	Input file for Resource 6; derived from Resource 5.	10.6078/d1bc7t
9	score_biochem_genetic.csv	csv	Output file for Resource 6	10.6078/d16p4j
10	score_biochem_genetic_soverall.csv	csv	Output file for Resource 6 and input file for Resource 11	10.6078/d1301k
11	generate_score_final.R	R script	The script "generate_score_final.R" is run to compute the final adjusted scores saved as "score_final.csv" from "score_biochem_genetic_soverall.csv"	10.6078/d1z593
12	score_final.csv	csv	Output file for Resource 11, with score S[final]	10.6078/d1tg6t
13	GOA analysis results.xlsx	Excel	Comparison between our literature-based study of Nudix functions and those in GOA. The sheet "GOA_Comparison" shows the final comparison result between our Nudix database and UniProt GOA (release 2013-12-11). The main content in this sheet is reprinted as Table 5 in the main text of the paper. The sheet "Statistics" contains information to derive the GOA-related statistics mentioned in the abstract, introduction, and result part of the main text. The sheet "MySQL_040614" contains the same protein-function-score tuples as in the "Final Score Assignment" Excel file, and in addition provides curated function assignments (Columns D and E) resulting from the GOA comparison. The three sheets "UniProt_GOA_121113-EXP/NONEXP/NONF" contains all GO assignments to Nudix proteins in GOA release 2013-12-11. The sheet "GO_Terms" contains the complete proposed and current GO terms used in this paper to describe the Nudix protein collection; the main content in this sheet is reprinted as Tables 1 and 2. The sheet "GO_Hierarchy_proposed" contains the proposed GO hierarchy, and is reprinted as Figure S2. Finally, the sheet "GO_Hierarchy_010114" is an archive of the existed GO hierarchy for Nudix-related terms as of 2014-01-01.	10.6078/d1pp4w
14	Pipelines to build sequence alignments of the Nudix	PDF	Graphical representation of the steps used to reconstruct sequence alignments of the Nudix superfamily, as described in the Materials and	10.6078/D1K01X

	superfamily.pdf		Methods section. (A) The pipeline to build the 78-PDB structure guided sequence alignment. (B) The pipeline to build the 324-core sequence alignment guided by the 78-PDB sequence alignment. (C) The pipeline to build the alignment of the complete Nudix clan (38,950 sequences). (D) Illustration of how to combine two alignment into one guided by a scaffold alignment.	
15	3DCOMB alignment.py	Python script	The file "3DCOMB alignment.py" is a structural alignment of 78 proteins with PDB entires; can be opened with Chimera v1.6.2 [52].	10.6078/D1F59F
16	46-PDB alignment.fa	FASTA	46-PDB sequence alignment in FASTA format; can be opened by most sequence alignment visualization software such as Jalview [53].	10.6078/D19G65
17	78-PDB alignment.fa	FASTA	76-PDB sequence alignment in FASTA format; can be opened by most sequence alignment visualization software such as Jalview [53].	10.6078/D15P47
18	340 select nudix proteins.xlsx	Excel	The Excel file "340 select nudix proteins.xlsx" contains information of 340 select Nudix proteins, the subsets of which comprise of all alignments discussed in the paper except the "Nudix-clan alignment". The information related to the PDB structures is reprinted in Table 3. Columns K, L, M and N indicates the starting and ending residues of domains used in the alignments. For each protein, the best-characterized activity with the highest confidence score, when available, is recorded in Columns O, P and Q; such information was obtained from Resource 4.	10.6078/D12018
19	324-core alignment.fa	FASTA	324-core sequence alignment in FASTA format; can be opened by most sequence alignment visualization software such as Jalview [53].	10.6078/D1X59S
20	347-select alignment.fa	FASTA	347-select sequence alignment in FASTA format can be opened by most sequence alignment visualization software such as Jalview [53].	10.6078/D1SG6H
21	Nudix-clan alignment.fa	FASTA	Full Nudix clan alignment in FASTA format; can be opened by most sequence alignment visualization software such as Jalview [53].	10.6078/D1NP4K
22	347-select tree.newick	Newick	Phylogenetic tree of the 347-select proteins. It can be opened by most phylogeny visualization software such as Dendroscope v3.2.8 [66].	10.6078/D1J01M
23	347-select tree reconciled.dendro	Dendroscope	Reconciled tree of proteins in the 347-select set. The files in Dendroscope format have the same tree topologies as the Newick format, and additionally contain rich information such as reconciliation, bootstrap and so on; these files can be opened by earlier version of Dendroscope (v3.2.8 or earlier) [66].	10.6078/D1D594
24	Nudix-clan tree.newick	Newick	Phylogenetic tree of the entire Nudix clan. It can be opened by most phylogeny visualization software such as Dendroscope v3.2.8 [66].	10.6078/D18G6V
25	Nudix-clan tree reconciled.dendro	Dendroscope	Reconciled tree of the entire Nudix clan. The files in Dendroscope format have the same tree topologies as the Newick format, and additionally contain rich information such as reconciliation, bootstrap and so on; these files can be opened by earlier version of Dendroscope (v3.2.8 or earlier) [66].	10.6078/D14S3K
26	Figure 5 347-select tree high resolution.pdf	PDF	Original high-resolution PDF used to generate Figure 5 in the main text of the paper, with the 347-select tree. It can be zoomed-in to a high degree to	10.6078/D1101Z

			visualize the details of the trees.	
27	Figure S4 Nudix-clan tree high resolution.pdf	PDF	Original high-resolution PDF used to generate Figure S4 in the main text of the paper, with the entire Nudix clan. It can be zoomed-in to a high degree to visualize the details of the trees.	10.6078/D1W884

Figure S1

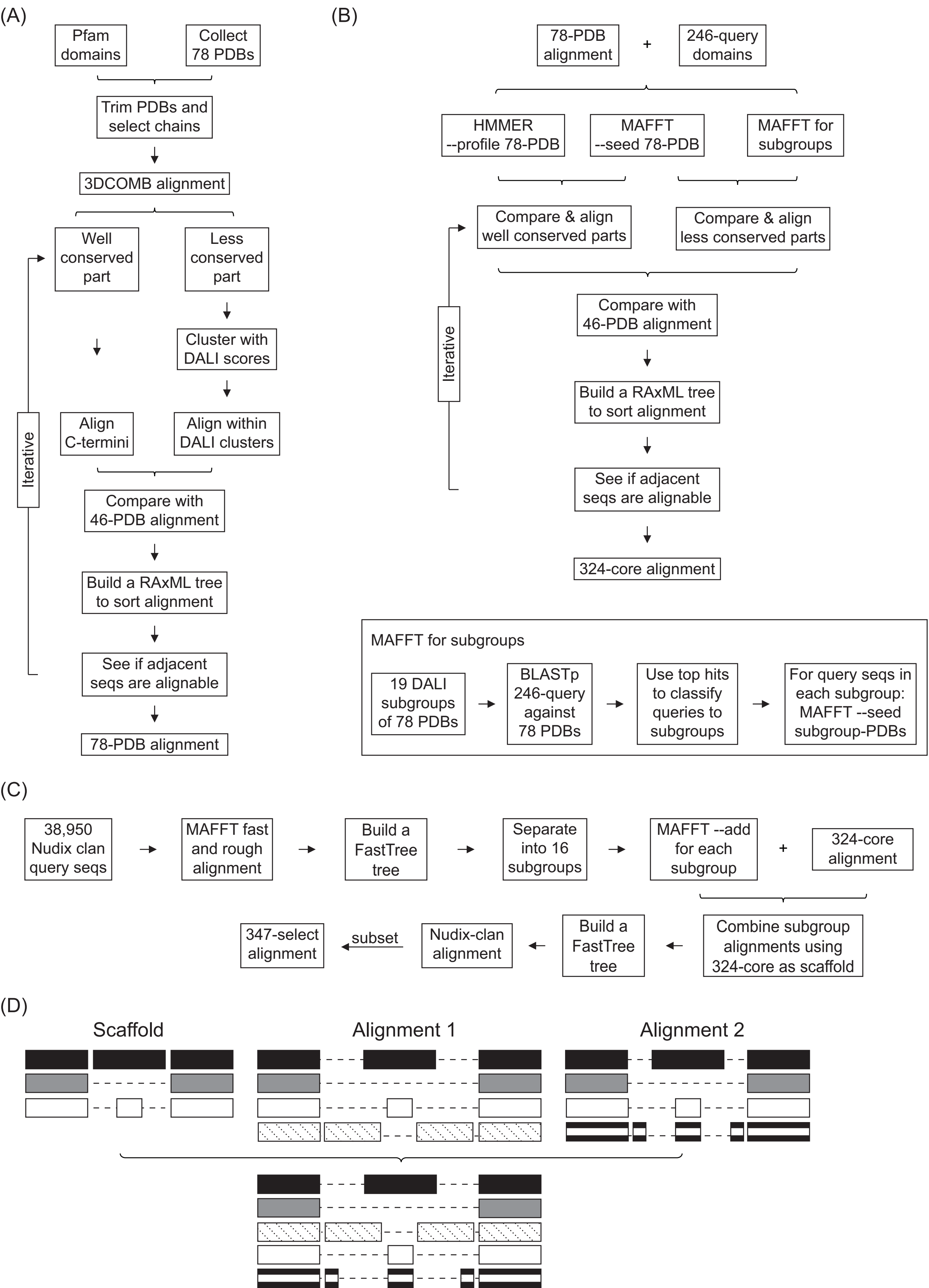
(A)
Pfam domains
Collect 78 PDBs
Trim PDBs and select chains
3DCOMB alignment
Well conserved part
Less conserved part
Cluster with DALI scores
Align C-termini
Align within DALI clusters
Iterative
Compare with 46-PDB alignment
Build a RAxML tree to sort alignment
See if adjacent seqs are alignable
78-PDB alignment
(B)
78-PDB alignment
+
246-query domains
HMMER --profile 78-PDB
MAFFT --seed 78-PDB
MAFFT for subgroups
Compare & align well conserved parts
Compare & align less conserved parts
Iterative
Compare with 46-PDB alignment
Build a RAxML tree to sort alignment
See if adjacent seqs are alignable
324-core alignment
MAFFT for subgroups
19 DALI subgroups of 78 PDBs
BLASTp 246-query against 78 PDBs
Use top hits to classify queries to subgroups
For query seqs in each subgroup: MAFFT --seed subgroup-PDBs
(C)
38,950 Nudix clan query seqs
MAFFT fast and rough alignment
Build a FastTree tree
Separate into 16 subgroups
MAFFT --add for each subgroup
+
324-core alignment
Combine subgroup alignments using 324-core as scaffold
Build a FastTree tree
Nudix-clan alignment
subset
347-select alignment
(D)
Scaffold
Alignment 1
Alignment 2

Figure S1. Pipelines to build sequence alignments of the Nudix superfamily. The detailed steps are described in the Materials and Methods section. (A) The pipeline to build the 78-PDB structure guided sequence alignment. (B) The pipeline to build the 324-core sequence alignment guided by the 78-PDB sequence alignment. (C) The pipeline to build the alignment of the complete Nudix clan (38,950 sequences). (D) Illustration of how to combine two alignment into one guided by a scaffold alignment.

Figure S2

[a] name changed
[b] definition changed
[c] parent changed
[d] parent added
[e] merged
[f] proposed

- GO:0008534 oxidized purine nucleobase lesion DNA N-glycosylase activity
  - [f] A001 2-hydroxy-adenine DNA N-glycoslyase activity
- GO:0016462 pyrophosphatase activity
  - [f] A085 oxothiamine-diphosphatase activity
  - [f] A086 oxythiamine-diphosphatase activity
  - [f] A087 thiamine triphosphate phosphatase activity (product undefined)
    - [ac] GO:0050333 thiamine-triphosphatase activity (Pi yielding)
  - [f] A107 UDP-hexanolamine diphosphatase activity
  - [f] A112 UDP-N-acetyl-muramoyl-L-alanine diphosphatase activity
  - [f] A117 CDP-ethanolamine diphosphatase activity
  - [f] A127 CDP-choline diphosphatase activity
  - GO:0000298 endopolyphosphatase activity
  - GO:0000810 diacylglycerol diphosphate phosphatase activity
  - GO:0002145 4-amino-5-hydroxymethyl-2-methylpyrimidine diphosphatase activity
  - GO:0004309 exopolyphosphatase activity
  - GO:0004427 inorganic diphosphatase activity
  - [bc] GO:0004787 thiamine-pyrophosphatase activity
  - GO:0008715 CDP-diacylglycerol diphosphatase activity
  - GO:0009678 hydrogen-translocating pyrophosphatase activity
  - [a] GO:0019177 dihydroneopterin triphosphate pyrophosphohydrolase activity (PPi yielding)
  - GO:0043135 5-phosphoribosyl 1-pyrophosphate pyrophosphatase activity
  - GO:0047430 oligosaccharide-diphosphodolichol diphosphatase activity
  - GO:0047734 CDP-glycerol diphosphatase activity
  - GO:0047874 dolichyldiphosphatase activity
  - GO:0050355 triphosphatase activity
  - GO:0050380 undecaprenyl-diphosphatase activity
  - [f] AP001 nucleoside-polyphosphate phosphatase activity
    - [f] A004 2-hydroxy-deoxyadenosine triphosphate phosphatase activity (product undefined)
    - [f] A005 8-oxo-guanosine triphosphate phosphatase activity (product undefined)
      - [df] A006 8-oxo-guanosine triphosphatase activity (Pi yielding)
      - [ad] GO:0008413 8-oxo-GTP pyrophosphatase activity (PPi yielding)
    - [f] A008 5-methyl-deoxycytidine triphosphate phosphatase activity (product undefined)
      - [df] A007 5-methyl-deoxycytidine triphosphate pyrophosphatase activity (PPi yielding)
    - [f] A009 5-methyl-uridine triphosphate phosphatase activity (product undefined)
    - [f] A010 5-hydroxy-cytidine triphosphate phosphatase activity (product undefined) (product undefined)
    - [f] A011 5-hydroxy-deoxycytidine triphosphate phosphatase activity (product undefined)
    - [f] A014 8-hydroxy-deoxyadenosine triphosphate phosphatase activity (product undefined)
      - [df] A013 8-hydroxy-deoxyadenosine triphosphate pyrophosphatase activity (PPi yielding)
    - [f] A016 8-oxo-deoxyguanosine triphosphate phosphatase activity (product undefined)
      - [df] A015 8-oxo-deoxyguanosine triphosphatase activity (Pi yielding)
      - [ad] GO:0035539 8-oxo-dGTP pyrophosphatase activity (PPi yielding)
    - [f] A029 arabinofuranosylcytosine triphosphate phosphatase activity (product undefined)
    - [f] A030 adenosine triphosphate phosphatase activity (product undefined)
      - [ad] GO:0016887 ATPase activity (Pi yielding)
      - [ad] GO:0047693 ATP diphosphatase activity (PPi yielding)
    - [f] A034 cytidine triphosphate phosphatase activity (product undefined)
      - [df] A061 CTP pyrophosphatase activity (PPi yielding)
      - [ad] GO:0043273 CTPase activity (Pi yielding)
    - [f] A055 deoxyadenosine triphosphate phosphatase activity (product undefined)
      - [ad] GO:0008828 dATP pyrophosphohydrolase activity (PPi yielding)
    - [f] A057 deoxycytidine triphosphate phosphatase activity (product undefined)
      - [df] A058 deoxycytidine triphosphatase activity (stepwise)
      - [ad] GO:0047840 dCTP diphosphatase activity (PPi yielding)
    - [f] A060 deoxyguanosine triphosphate phosphatase activity (product undefined)
      - [ad] GO:0036217 dGTP diphosphatase activity (PPi yielding)
    - [f] A062 uridine triphosphate phosphatase activity (product undefined)
      - [ad] GO:0036221 UTP diphosphatase activity (PPi yielding)
    - [f] A063 deoxyuridine triphosphate phosphatase activity (product undefined)
      - [df] A064 deoxyuridine triphosphatase activity (stepwise)
    - [f] A076 guanosine triphosphate phosphatase activity (product undefined)
      - [df] A077 guanosine triphosphatase activity (stepwise)
      - [ad] GO:0003924 GTPase activity (Pi yielding)
      - [ad] GO:0036219 GTP diphosphatase activity (PPi yielding)
    - [f] A080 inosine triphosphate phosphatase activity (product undefined)
      - [ad] GO:0036220 ITP diphosphatase activity (PPi yielding)
    - [f] A082 N4-methyl-deoxycytidine triphosphate phosphatase activity (product undefined)
    - [f] A088 adenosine tetraphosphate phosphatase activity (product undefined)
      - [f] A089 adenosine tetraphosphatase activity (AMP yielding)
      - [ac] GO:0047624 adenosine tetraphosphatase activity (ATP yielding)
    - [f] A090 guanosine tetraphosphate phosphatase activity (product undefined)
    - [f] A091 adenosine pentaphosphate phosphatase activity (product undefined)
      - [f] A136 adenosine pentaphosphate phosphatase activity (ATP yielding)
    - [f] A099 thymidine triphosphate phosphatase activity (product undefined)
      - [df] A100 thymidine triphosphatase activity (stepwise)
      - [ad] GO:0050339 TTPase activity (Pi yielding)
      - [ad] GO:0036218 TTP diphosphatase activity (PPi yielding)
    - [c] GO:0017110 nucleoside-diphosphatase activity
      - [f] A094 guanosine 3',5'-bis(diphosphate) diphosphate phosphatase activity (product undefined)
        - [f] A095 guanosine 3',5'-bis(diphosphate) diphosphatase activity (stepwise)
      - [f] A133 thymidine-diphosphatase activity
      - GO:0004382 guanosine-diphosphatase activity
      - GO:0036384 cytidine diphosphatase activity
      - GO:0043262 adenosine-diphosphatase activity
      - GO:0044716 8-oxo-GDP phosphatase activity
      - GO:0045134 uridine-diphosphatase activity
      - GO:0090450 inosine-diphosphatase activity
    - [ac] GO:0017111 nucleoside-triphosphatase activity (Pi yielding)
      - [df] A006 8-oxo-guanosine triphosphatase activity (Pi yielding)
      - [df] A015 8-oxo-deoxyguanosine triphosphatase activity (Pi yielding)
      - [df] A058 deoxycytidine triphosphatase activity (stepwise)
      - [df] A064 deoxyuridine triphosphatase activity (stepwise)
      - [df] A077 guanosine triphosphatase activity (stepwise)
      - [df] A100 thymidine triphosphatase activity (stepwise)
      - [df] A113 uridine triphosphatase activity (stepwise)
      - GO:0003774 motor activity
      - [ad] GO:0003924 GTPase activity (Pi yielding)
      - GO:0004386 helicase activity
      - [ac] GO:0008894 guanosine-5'-triphosphate,3'-diphosphate diphosphatase activity (Pi yielding)
      - [ad] GO:0016887 ATPase activity (Pi yielding)
      - GO:0036310 annealing helicase activity
      - [ad] GO:0043273 CTPase activity (Pi yielding)
      - [ad] GO:0050339 TTPase activity (Pi yielding)
      - GO:0060589 nucleoside-triphosphatase regulator activity
    - [ac] GO:0047429 nucleoside-triphosphate diphosphatase activity (PPi yielding)
      - [df] A007 5-methyl-deoxycytidine triphosphate pyrophosphatase activity (PPi yielding)
      - [f] A012 8-hydroxy-adenosine triphosphate pyrophophatase activity (PPi yielding)
      - [df] A013 8-hydroxy-deoxyadenosine triphosphate pyrophosphatase activity (PPi yielding)
      - [df] A061 CTP pyrophosphatase activity (PPi yielding)
      - [f] A131 3'-amino-3'-dATP pyrophosphatase activity (PPi yielding)
      - [f] A132 3'-amino-3'-TTP pyrophosphatase activity (PPi yielding)
      - [a] GO:0004170 dUTP diphosphatase activity (PPi yielding)
      - [ac] GO:0004636 phosphoribosyl-ATP diphosphatase activity (PPi yielding)
      - [ad] GO:0008413 8-oxo-GTP pyrophosphatase activity (PPi yielding)
      - [ad] GO:0008828 dATP pyrophosphohydrolase activity (PPi yielding)
      - [ad] GO:0035539 8-oxo-dGTP pyrophosphatase activity (PPi yielding)
      - [a] GO:0035870 dITP diphosphatase activity (PPi yielding)
      - [ad] GO:0036217 dGTP diphosphatase activity (PPi yielding)
      - [ad] GO:0036218 TTP diphosphatase activity (PPi yielding)
      - [ad] GO:0036219 GTP diphosphatase activity (PPi yielding)
      - [ad] GO:0036220 ITP diphosphatase activity (PPi yielding)
      - [ad] GO:0036221 UTP diphosphatase activity (PPi yielding)
      - [a] GO:0036222 XTP diphosphatase activity (PPi yielding)
      - [a] GO:0044713 2-OH-ATP pyrophosphatase activity (PPi yielding)
      - [ab] GO:0044714 2-OH-dATP pyrophosphatase activity (PPi yielding)
      - [ad] GO:0047693 ATP diphosphatase activity (PPi yielding)
      - [ad] GO:0047840 dCTP diphosphatase activity (PPi yielding)
    - [c] GO:0097382 deoxynucleoside-diphosphatase activity
      - [f] A002 2-hydroxy-deoxyadenosine diphosphatase activity
      - [f] A035 deoxyadenosine diphosphatase activity
      - [f] A056 deoxycytidine diphosphatase activity
      - [f] A059 deoxyguanosine diphosphatase activity
      - [c] GO:0044715 8-oxo-dGDP phosphatase activity
      - [c] GO:0044717 8-hydroxy-dADP phosphatase activity
      - GO:0097383 dIDP diphosphatase activity
  - [f] AP007 CDP-sugar diphosphatase activity
    - [f] A031 CDP-glucose diphosphatase activity
    - [f] A032 CDP-ribose diphosphatase activity
  - [f] AP008 GDP-sugar diphosphatase activity
    - [f] A068 GDP-beta-fucose diphosphatase activity
    - [f] A069 GDP-fructose diphosphatase activity
    - [f] A070 GDP-glucose diphosphatase activity
    - [f] A071 GDP-ribose diphosphatase activity
    - [ac] GO:0052751 GDP-mannose diphosphatase activity
  - [f] AP009 TDP-sugar diphosphatase activity
    - [f] A116 TDP-glucose diphosphatase activity
  - [f] AP011 general coenzyme A diphosphatase activity
    - [f] A036 fatty-acid-acyl-CoA diphosphatase activity
    - [f] A054 choloyl-CoA diphosphatase activity
    - [f] A096 malonyl-CoA diphosphatase activity
    - [f] A098 trihydroxycoprostanoyl-CoA diphosphatase activity
    - [f] A114 3-methyl-3-hydroxyglutaryl-CoA diphosphatase activity
    - [f] A118 CoA-disulfide diphosphatase activity
    - [f] A120 3'-dephospho-CoA diphosphatase activity
    - [f] A122 CoA-glutathione diphosphatase activity
    - [f] A125 acetyl-CoA diphosphatase activity
    - [f] A128 succinyl-CoA diphosphatase activity
    - [c] GO:0010945 CoA pyrophosphatase activity
  - [f] AP013 RNA decapping activity
    - [f] A065 m7G(5')ppp-mRNA diphosphatase activity (m7GDP yielding)
    - [f] A066 G(5')ppp-mRNA triphosphatase activity (GMP yielding)
    - [f] A067 G(5')ppp-mRNA triphosphatase activity (GDP yielding)
    - [f] A081 m7G(5')ppp-snoRNA triphosphatase activity (m7GDP yielding)
    - [ac] GO:0050072 m7G(5')ppp-mRNA diphosphatase activity (m7GMP yielding)
    - [c] GO:0034353 RNA pyrophosphohydrolase activity
  - [f] AP014 IDP-sugar diphosphatase activity
    - [f] A079 IDP-ribose diphosphatase activity
  - [a] GO:0004551 dinucleotide-polyphosphate phosphatase activity
    - [f] AP002 dinucleoside-diphosphate phosphatase activity
      - [f] A017 bis(5'-adenosyl)-diphosphatase activity
      - [f] A072 bis(5'-guanosyl)-diphosphatase activity
      - [f] AP012 general NAD diphosphatase activity
        - [f] A115 deamino-NAD+ diphosphatase activity
        - [f] A126 NAADP+ diphosphatase activity
        - [f] A129 deamino-NADH diphosphatase activity
        - [f] A134 NADP+ diphosphatase activity
        - [c] GO:0000210 NAD+ diphosphatase activity
        - [c] GO:0010943 NADPH pyrophosphatase activity
        - [c] GO:0035529 NADH pyrophosphatase activity
      - [c] GO:0047884 FAD diphosphatase activity
    - [f] AP003 dinucleoside-triphosphate phosphatase activity
      - [f] A073 bis(5'-guanosyl)-triphosphatase activity
      - [f] A130 P1-(5'-adenosyl)P3-(5'-guanosyl) triphosphate phosphatase activity (product undefined)
    - [f] AP005 dinucleoside-pentaphosphate phosphatase activity
      - [f] A024 bis(5'-adenosyl)-pentaphosphate phosphatase activity (product undefined)
        - [f] A023 bis(5'-adenosyl)-pentaphosphatase activity (ADP yielding)
        - [ac] GO:0034432 bis(5'-adenosyl)-pentaphosphatase activity (AMP yielding)
      - [f] A025 P1-(5'-adenosyl)P5-(5'-guanosyl) pentaphosphate phosphatase activity (product undefined)
      - [f] A075 bis(5'-guanosyl)-pentaphosphate phosphatase activity (product undefined)
    - [f] AP006 dinucleoside-hexaphosphate phosphatase activity
      - [f] A026 bis(5'-adenosyl)-hexaphosphate phosphatase activity (product undefined)
        - [f] A027 bis(5'-adenosyl)-hexaphosphatase activity (ADP yielding)
        - [f] A137 bis(5'-adenosyl)-hexaphosphatase activity (ATP yielding)
        - [ac] GO:0034431 bis(5'-adenosyl)-hexaphosphatase activity (AMP yielding)
      - [f] A028 P1-(5'-adenosyl)P6-(5'-guanosyl) hexaphosphate phosphatase activity (product undefined)
    - GO:0008796 bis(5'-nucleosyl)-tetraphosphatase activity
      - [f] A018 bis(5'-adenosyl)-tetraphosphate phosphatase activity (product undefined)
        - [f] A135 bis(5'-adenosyl)-tetraphosphate phosphatase activity (AMP yielding)
      - [f] A019 P1-(5'-adenosyl)P4-(5'-cytidyl) tetraphosphate phosphatase activity (product undefined)
      - [f] A021 P1-(5'-adenosyl)P4-(5'-guanosyl) tetraphosphate phosphatase activity (product undefined)
        - [f] A020 P1-(5'-adenosyl)P4-(5'-guanosyl) tetraphosphatase activity (ADP yielding)
      - [f] A022 P1-(5'-adenosyl)P4-(5'-uridyl) tetraphosphate phosphatase activity (product undefined)
      - [f] A074 bis(5'-guanosyl)-tetraphosphate phosphatase activity (product undefined)
      - GO:0004081 bis(5'-nucleosyl)-tetraphosphatase (asymmetrical) activity
      - GO:0008803 bis(5'-nucleosyl)-tetraphosphatase (symmetrical) activity
    - GO:0047710 bis(5'-adenosyl)-triphosphatase activity
  - GO:0008486 diphosphoinositol-polyphosphate diphosphatase activity
    - [c] GO:0052840 inositol diphosphate tetrakisphosphate diphosphatase activity
    - [c] GO:0052841 inositol bisdiphosphate tetrakisphosphate diphosphatase activity
      - GO:0052846 inositol-1,5-bisdiphosphate-2,3,4,6-tetrakisphosphate 1-diphosphatase activity
      - GO:0052847 inositol-1,5-bisdiphosphate-2,3,4,6-tetrakisphosphate 5-diphosphatase activity
      - GO:0052848 inositol-3,5-bisdiphosphate-2,3,4,6-tetrakisphosphate 5-diphosphatase activity
    - [c] GO:0052842 inositol diphosphate pentakisphosphate diphosphatase activity
      - GO:0052843 inositol-1-diphosphate-2,3,4,5,6-pentakisphosphate diphosphatase activity
      - GO:0052844 inositol-3-diphosphate-1,2,4,5,6-pentakisphosphate diphosphatase activity
      - GO:0052845 inositol-5-diphosphate-1,2,3,4,6-pentakisphosphate diphosphatase activity
  - GO:0008768 UDP-sugar diphosphatase activity
    - [f] A101 UDP-galactosamine diphosphatase activity
    - [f] A102 UDP-galactose diphosphatase activity
    - [f] A103 UDP-galacturonic acid diphosphatase activity
    - [f] A104 UDP-glucosamine diphosphatase activity
    - [f] A105 UDP-glucose diphosphatase activity
    - [f] A106 UDP-glucuronic acid diphosphatase activity
    - [f] A108 UDP-mannose diphosphatase activity
    - [f] A109 UDP-N-acetyl-galactosamine diphosphatase activity
    - [f] A110 UDP-N-acetyl-glucosamine diphosphatase activity
    - [f] A111 UDP-N-acetyl-muramic acid diphosphatase activity
    - [c] GO:0008758 UDP-2,3-diacylglucosamine hydrolase activity
  - GO:0019144 ADP-sugar diphosphatase activity
    - [f] A084 O-acetyl-ADP-ribose diphosphatase activity
    - [f] A119 2'-phospho-ADP-ribose diphosphatase activity
    - [f] A121 ADP-mannose diphosphatase activity
    - [ce] ~~GO:0047631~~ ADP-ribose diphosphatase activity
    - GO:0080041 ADP-ribose pyrophosphohydrolase activity
    - GO:0080042 ADP-glucose pyrophosphohydrolase activity

Figure S2. Proposed Gene Ontology hierarchy. This region of the classification is a strict hierarchy, not a DAG, except as indicated below. New terms are begun with either A (terms with experimental support) or AP (parent terms proposed only for the structure of the hierarchy). The relationships between parent and child terms are represented with indents. The proposed changes, when applicable, are indicated by superscripts in front of GO term IDs: a – change name; b – change definition; c – change parent; d – add parent; e – remove; f – newly proposed. When a term has two parents, the term is shown in black under one parent and grey in the other. The removed term is indicated with strikethrough.

Figure S3

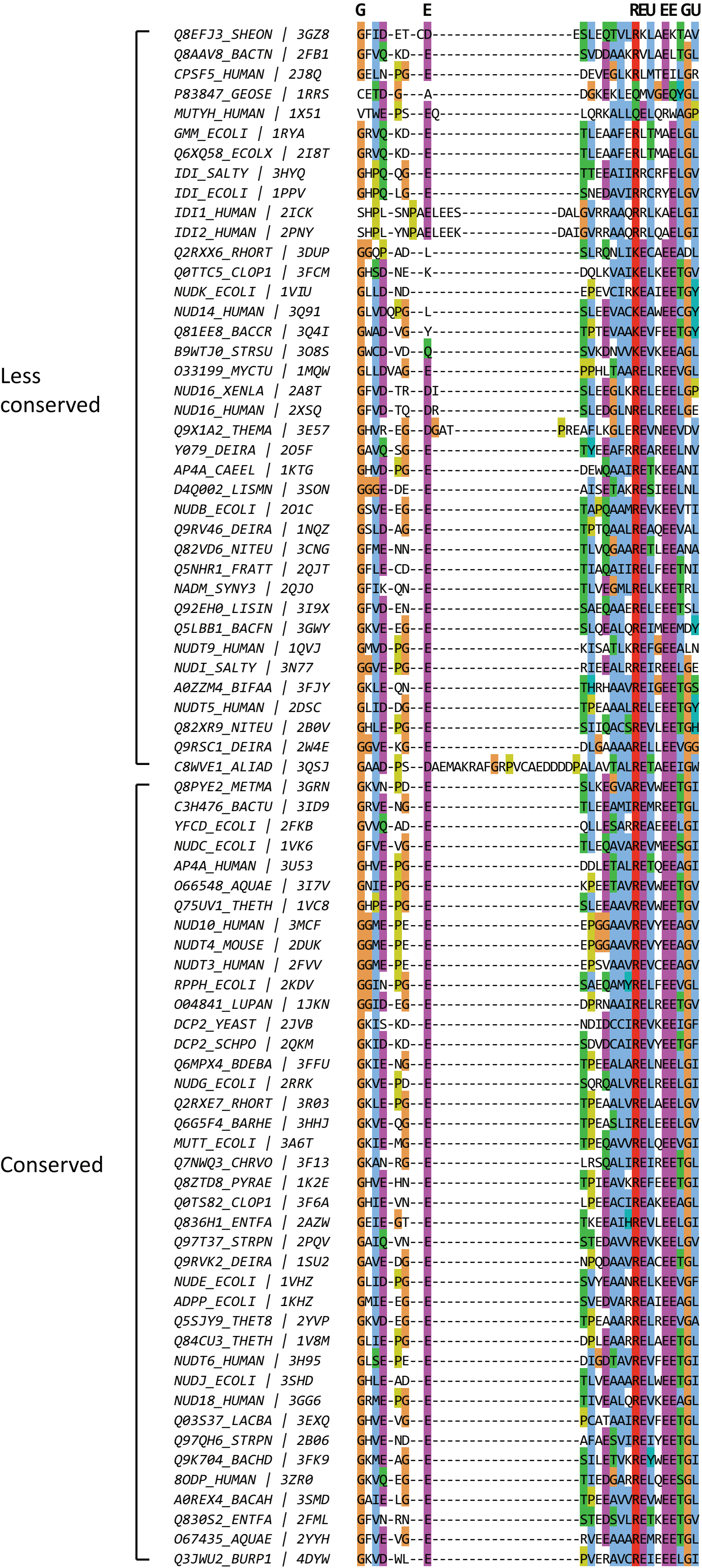
G E REU EE GU
Less conserved
Q8EFJ3_SHEON | 3GZ8 GFID-ET-CD-------------------ESLEQTVLRKLAEKTAV
Q8AAV8_BACTN | 2FB1 GFVQ-KD--E--------------------SVDDAAKRVLAELTGL
CPSF5_HUMAN | 2J8Q GELN-PG--E--------------------DEVEGLKRLMTEILGR
P83847_GEOSE | 1RRS CETD-G---A--------------------DGKEKLEQMVGEQYGL
MUTYH_HUMAN | 1X51 VTWE-PS--EQ-------------------LQRKALLQELQRWAGP
GMM_ECOLI | 1RYA GRVQ-KD--E--------------------TLEAAFERLTMAELGL
Q6XQ58_ECOLX | 2I8T GRVQ-KD--E--------------------TLEAAFERLTMAELGL
IDI_SALTY | 3HYQ GHPQ-QG--E--------------------TTEEAIIRRCRFELGV
IDI_ECOLI | 1PPV GHPQ-LG--E--------------------SNEDAVIRRCRYELGV
IDI1_HUMAN | 2ICK SHPL-SNPAELEES-------------DALGVRRAAQRRLKAELGI
IDI2_HUMAN | 2PNY SHPL-YNPAELEEK-------------DAIGVRRAAQRRLQAELGI
Q2RXX6_RHORT | 3DUP GGQP-AD--L--------------------SLRQNLIKECAEEADL
Q0TTC5_CLOP1 | 3FCM GHSD-NE--K--------------------DQLKVAIKELKEETGV
NUDK_ECOLI | 1VIU GLLD-ND--E--------------------EPEVCIRKEAIEETGY
NUD14_HUMAN | 3Q91 GLVDQPG--L--------------------SLEEVACKEAWEECGY
Q81EE8_BACCR | 3Q4I GWAD-VG--Y--------------------TPTEVAAKEVFEETGY
B9WTJ0_STRSU | 3O8S GWCD-VD--Q--------------------SVKDNVVKEVKEEAGL
O33199_MYCTU | 1MQW GLLDVAG--E--------------------PPHLTAARELREEVGL
NUD16_XENLA | 2A8T GFVD-TR--DI-------------------SLEEGLKRELEEELGP
NUD16_HUMAN | 2XSQ GFVD-TQ--DR-------------------SLEDGLNRELREELGE
Q9X1A2_THEMA | 3E57 GHVR-EG--DGAT--------------PREAFLKGLEREVNEEVDV
Y079_DEIRA | 2O5F GAVQ-SG--E--------------------TYEEAFRREAREELNV
AP4A_CAEEL | 1KTG GHVD-PG--E--------------------DEWQAAIRETKEEANI
D4Q002_LISMN | 3SON GGGE-DE--E--------------------AISETAKRESIEELNL
NUDB_ECOLI | 2O1C GSVE-EG--E--------------------TAPQAAMREVKEEVTI
Q9RV46_DEIRA | 1NQZ GSLD-AG--E--------------------TPTQAALREAQEEVAL
Q82VD6_NITEU | 3CNG GFME-NN--E--------------------TLVQGAARETLEEANA
Q5NHR1_FRATT | 2QJT GFLE-CD--E--------------------TIAQAIIRELFEETNI
NADM_SYNY3 | 2QJO GFIK-QN--E--------------------TLVEGMLRELKEETRL
Q92EH0_LISIN | 3I9X GFVD-EN--E--------------------SAEQAAERELEEETSL
Q5LBB1_BACFN | 3GWY GKVE-EG--E--------------------SLQEALQREIMEEMDY
NUDT9_HUMAN | 1QVJ GMVD-PG--E--------------------KISATLKREFGEEALN
NUDI_SALTY | 3N77 GGVE-PG--E--------------------RIEEALRREIREELGE
A0ZZM4_BIFAA | 3FJY GKLE-QN--E--------------------THRHAAVREIGEETGS
NUDT5_HUMAN | 2DSC GLID-DG--E--------------------TPEAAALRELEEETGY
Q82XR9_NITEU | 2B0V GHLE-PG--E--------------------SIIQACSREVLEETGH
Q9RSC1_DEIRA | 2W4E GGVE-KG--E--------------------DLGAAAAARELLEEVGG
C8WVE1_ALIAD | 3QSJ GAAD-PS--DAEMAKRAFGRPVCAEDDDDPALAVTALRETAEEIGW
Conserved
Q8PYE2_METMA | 3GRN GKVN-PD--E--------------------SLKEGVAREVWEETGI
C3H476_BACTU | 3ID9 GRVE-NG--E--------------------TLEEAMIREMREETGL
YFCD_ECOLI | 2FKB GVVQ-AD--E--------------------QLLESARREAEEELGI
NUDC_ECOLI | 1VK6 GFVE-VG--E--------------------TLEQAVAREVMEESGI
AP4A_HUMAN | 3U53 GHVE-PG--E--------------------DDLETALRETQEEAGI
O66548_AQUAE | 3I7V GNIE-PG--E--------------------KPEETAVREVWEETGV
Q75UV1_THETH | 1VC8 GHPE-PG--E--------------------SLEEAAVREVWEETGV
NUD10_HUMAN | 3MCF GGME-PE--E--------------------EPGGAAVREVYEEAGV
NUDT4_MOUSE | 2DUK GGME-PE--E--------------------EPGGAAVREVYEEAGV
NUDT3_HUMAN | 2FVV GGME-PE--E--------------------EPSVAAVREVCEEAGV
RPPH_ECOLI | 2KDV GGIN-PG--E--------------------SAEQAMYRELFEEVGL
O04841_LUPAN | 1JKN GGID-EG--E--------------------DPRNAAIRELREETGV
DCP2_YEAST | 2JVB GKIS-KD--E--------------------NDIDCCIREVKEEIGF
DCP2_SCHPO | 2QKM GKID-KD--E--------------------SDVDCAIREVYEETGF
Q6MPX4_BDEBA | 3FFU GKIE-NG--E--------------------TPEEALARELNEELGI
NUDG_ECOLI | 2RRK GKVE-PD--E--------------------SQRQALVRELREELGI
Q2RXE7_RHORT | 3R03 GKLE-PG--E--------------------TPEAALVRELAEELGV
Q6G5F4_BARHE | 3HHJ GKVE-QG--E--------------------TPEASLIRELEEELGV
MUTT_ECOLI | 3A6T GKIE-MG--E--------------------TPEQAVVRELQEEVGI
Q7NWQ3_CHRVO | 3F13 GKAN-RG--E--------------------LRSQALIREIREETGL
Q8ZTD8_PYRAE | 1K2E GHVE-HN--E--------------------TPIEAVKREFEEETGI
Q0TS82_CLOP1 | 3F6A GHIE-VN--E--------------------LPEEACIREAKEEAGL
Q836H1_ENTFA | 2AZW GEIE-GT--E--------------------TKEEAIHREVLEELGI
Q97T37_STRPN | 2PQV GAIQ-VN--E--------------------STEDAVVREVKEELGV
Q9RVK2_DEIRA | 1SU2 GAVE-DG--E--------------------NPQDAAVREACEETGL
NUDE_ECOLI | 1VHZ GLID-PG--E--------------------SVYEAANRELKEEVGF
ADPP_ECOLI | 1KHZ GMIE-EG--E--------------------SVEDVARREAIEEAGL
Q5SJY9_THET8 | 2YVP GKVD-EG--E--------------------TPEAAARRELREEVGA
Q84CU3_THETH | 1V8M GLIE-PG--E--------------------DPLEAARRELAEETGL
NUDT6_HUMAN | 3H95 GLSE-PE--E--------------------DIGDTAVREVFEETGI
NUDJ_ECOLI | 3SHD GHLE-AD--E--------------------TLVEAAAARELWEETGI
NUD18_HUMAN | 3GG6 GRME-PG--E--------------------TIVEALQREVKEEAGL
Q03S37_LACBA | 3EXQ GHVE-VG--E--------------------PCATAAIREVFEETGL
Q97QH6_STRPN | 2B06 GHVE-ND--E--------------------AFAESVIREIYEETGL
Q9K704_BACHD | 3FK9 GKME-AG--E--------------------SILETVKREYWEETGI
8ODP_HUMAN | 3ZR0 GKVQ-EG--E--------------------TIEDGARRELQEESGL
A0REX4_BACAH | 3SMD GAIE-LG--E--------------------TPEEAVVREVWEETGL
Q830S2_ENTFA | 2FML GFVN-RN--E--------------------STEDSVLRETKEETGV
O67435_AQUAE | 2YYH GFVE-VG--E--------------------RVEEAAAREMREETGL
Q3JWU2_BURP1 | 4DYW GKVD-WL--E--------------------PVERAVCREIEEELGI

Figure S3. Nudix box sequences in the structure-induced sequence alignment. UniProt Entry Names and PDB IDs of 78 Nudix enzymes and their Nudix box sequences are shown in the alignment. The most conserved positions are indicated in bold on top of the alignment. The top 38 enzymes have at least one substitution in one of the most conserved positions, or one insertion or deletion between the conserved positions. The Clustal X color scheme was applied to all residues.

Figure S4

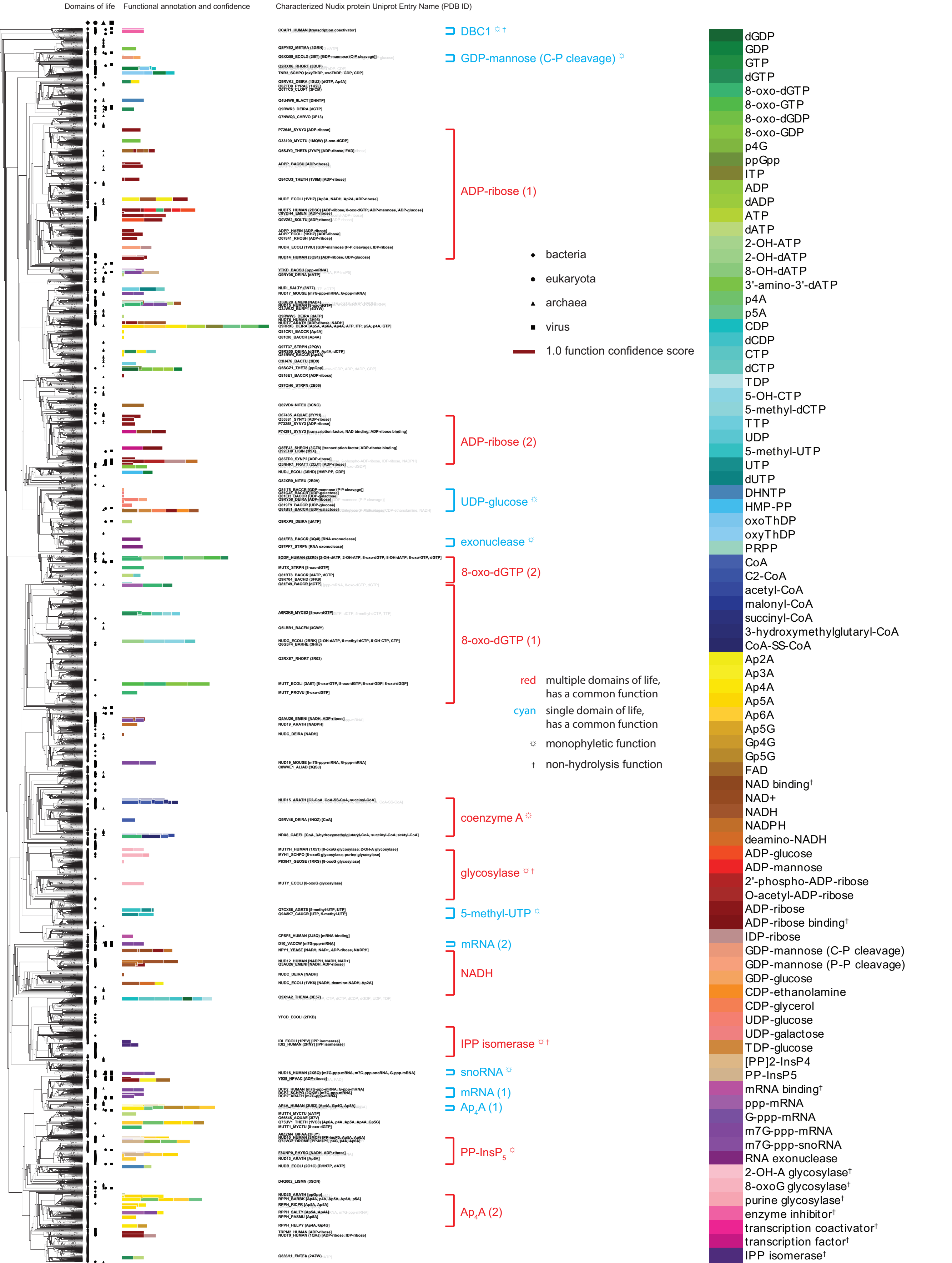
Domains of life
Functional annotation and confidence
Characterized Nudix protein Uniprot Entry Name (PDB ID)
DBC1 ☼†
GDP-mannose (C-P cleavage) ☼
ADP-ribose (1)
ADP-ribose (2)
UDP-glucose ☼
exonuclease ☼
8-oxo-dGTP (2)
8-oxo-dGTP (1)
coenzyme A ☼
glycosylase ☼†
5-methyl-UTP ☼
mRNA (2)
NADH
IPP isomerase ☼†
snoRNA ☼
mRNA (1)
Ap_4A (1)
$PP\text{-}InsP_5$ ☼
Ap_4A (2)
bacteria
eukaryota
archaea
virus
1.0 function confidence score
red multiple domains of life, has a common function
cyan single domain of life, has a common function
☼ monophyletic function
† non-hydrolysis function
dGDP
GDP
GTP
dGTP
8-oxo-dGTP
8-oxo-GTP
8-oxo-dGDP
8-oxo-GDP
p4G
ppGpp
ITP
ADP
dADP
ATP
dATP
2-OH-ATP
2-OH-dATP
8-OH-dATP
3'-amino-3'-dATP
p4A
p5A
CDP
dCDP
CTP
dCTP
TDP
5-OH-CTP
5-methyl-dCTP
TTP
UDP
5-methyl-UTP
UTP
dUTP
DHNTP
HMP-PP
oxoThDP
oxyThDP
PRPP
CoA
C2-CoA
acetyl-CoA
malonyl-CoA
succinyl-CoA
3-hydroxymethylglutaryl-CoA
CoA-SS-CoA
Ap2A
Ap3A
Ap4A
Ap5A
Ap6A
Ap5G
Gp4G
Gp5G
FAD
NAD binding†
NAD+
NADH
NADPH
deamino-NADH
ADP-glucose
ADP-mannose
2'-phospho-ADP-ribose
O-acetyl-ADP-ribose
ADP-ribose
ADP-ribose binding†
IDP-ribose
GDP-mannose (C-P cleavage)
GDP-mannose (P-P cleavage)
GDP-glucose
CDP-ethanolamine
CDP-glycerol
UDP-glucose
UDP-galactose
TDP-glucose
[PP]2-InsP4
PP-InsP5
mRNA binding†
ppp-mRNA
G-ppp-mRNA
m7G-ppp-mRNA
m7G-ppp-snoRNA
RNA exonuclease
2-OH-A glycosylase†
8-oxoG glycosylase†
purine glycosylase†
enzyme inhibitor†
transcription coactivator†
transcription factor†
IPP isomerase†

Figure S4. Phylogeny of the complete Nudix clan, with 38,950 unique Nudix domains that match the HMM of one of the Pfam families in Pfam v27.0 (Mar 2013). The phylogeny was constructed with FastTree [64] and mid-point rooted. The branch lengths of the tree are omitted for simplicity. The protein domains with experimental annotations are the same as those in **Fig. 5**. The domains of life of leaves are indicated on the side of the phylogeny as follows: solid diamonds (bacteria), circles (eukaryota), triangles (archaea), or squares (virus). The clades, functions, and IDs of leaves are indicated as in **Fig. 5** for protein domains with experimental annotations. When the text labels of multiple leaves overlapped, one leaf is shown in black, and others are shown in light grey. A high-resolution version is available in **Table S1, Resource 2.**
